# 18S rRNA gene sequences of leptocephalus gut contents, particulate organic matter, and biological oceanographic conditions in the western North Pacific

**DOI:** 10.1038/s41598-021-84532-y

**Published:** 2021-03-09

**Authors:** Tsuyoshi Watanabe, Satoshi Nagai, Yoko Kawakami, Taiga Asakura, Jun Kikuchi, Nobuharu Inaba, Yukiko Taniuchi, Hiroaki Kurogi, Seinen Chow, Tsutomu Tomoda, Daisuke Ambe, Daisuke Hasegawa

**Affiliations:** 1 Tohoku National Fisheries Research Institute, Japan Fisheries Research and Education Agency, 3-27-5 Shinhama-cho, Shiogama, Miyagi 985-0001 Japan; 2 National Research Institute of Fisheries Science, Japan Fisheries Research and Education Agency, 2-12-4 Fukuura, Kanazawa-ku, Kanagawa 236-8648 Japan; 3 AXIOHELIX Co. Ltd, 1-12-17 Izumi-cho, Kanda, Chiyoda-ku, Tokyo 101-0024 Japan; 4 RIKEN Center for Sustainable Resource Science, 1-7-22 Suehiro-cho, Tsurumi-ku, Yokohama, Kanagawa 230-0045 Japan; 5 Civil Engineering Research Institute for Cold Region, Public Works Research Institute, 1-34 Hiragishi 1-jo 3-chome, Toyohira-ku, Sapporo, Hokkaido 062-8602 Japan; 6 Hokkaido National Fisheries Technology Institute, Japan Fisheries Research and Education Agency, 116 Katsurakoi, Kushiro, Hokkaido 085-0802 Japan; 7 Shibushi Station, National Research Institute of Aquaculture, Japan Fisheries Research and Education Agency, 205 Natsui Shibushi-cho, Shibushi, Kagoshima 899-7101 Japan; 8 Present Address: Fisheries Technology Institute, Japan Fisheries Research and Education Agency, 116 Katsurakoi, Kushiro, Hokkaido 085-0802 Japan

**Keywords:** Animal behaviour, Animal migration, Behavioural ecology, Biooceanography, Marine biology

## Abstract

Eel larvae apparently feed on marine snow, but many aspects of their feeding ecology remain unknown. The eukaryotic 18S rRNA gene sequence compositions in the gut contents of four taxa of anguilliform eel larvae were compared with the sequence compositions of vertically sampled seawater particulate organic matter (POM) in the oligotrophic western North Pacific Ocean. Both gut contents and POM were mainly composed of dinoflagellates as well as other phytoplankton (cryptophytes and diatoms) and zooplankton (ciliophoran and copepod) sequences. Gut contents also contained cryptophyte and ciliophoran genera and a few other taxa. Dinoflagellates (family Gymnodiniaceae) may be an important food source and these phytoplankton were predominant in gut contents and POM as evidenced by DNA analysis and phytoplankton cell counting. The compositions of the gut contents were not specific to the species of eel larvae or the different sampling areas, and they were most similar to POM at the chlorophyll maximum in the upper part of the thermocline (mean depth: 112 m). Our results are consistent with eel larvae feeding on marine snow at a low trophic level, and feeding may frequently occur in the chlorophyll maximum in the western North Pacific.

## Introduction

The Japanese eel, *Anguilla japonica*, is a catadromous fish species with a spawning area at the West Mariana Ridge in the western North Pacific^1–4^. The eel larvae exhibit a peculiar transparent leaf-like body form and are called leptocephali^5^. Leptocephali drift from their spawning area via the North Equatorial Current, inhabit the Kuroshio to a period of 4–6 months, and are transported to the coast of Northeast Asia^6^. Japanese eels are an economically important aquaculture species in East Asia. However, since the 1970s, its population has demonstrated a dramatic decline due to reasons such as overfishing^7,8^, and the species is currently listed as critically endangered by the International Union for the Conservation of Nature^9^. As of 2010, the annual recruitment had decreased by as much as 90% compared with eel catches in the 1960s^9^.

Many eel larvae grow in the tropical and subtropical western North Pacific Ocean, where oligotrophic conditions and low biological productivity are encountered in the surface layer^10^. However, at a depth of 65–150 m, corresponding to 1% of the surface light intensity, a significantly high-concentration chlorophyll layer (namely, the subsurface chlorophyll maximum, SCM) is formed^11,12^. Ocean waters with high vertical stability due to thermal stratification encourage accumulation of phytoplankton and particulate organic matter (POM) at a thermocline owing to a high water density and a slow sedimentation rate. Phytoplankton abundance thus increases at the SCM due to reduced rates of descent and less turbulent mixing. These phenomena lead to biomass accumulation, as reflected in the maximum carbon storage potential. The phytoplankton community in the SCM is mainly composed of small phytoplankton, such as nano- and picoplankton, and community structure differs from that in the surface layer^12^.

The natural diets of eel larvae have not been identified because their guts usually contain a considerably indistinguishable amorphous material. Gut contents observed in different anguillid leptocephali in the North Pacific provide visible clues regarding dietary preferences. Zooplankton fecal pellets, larvacean houses, appendicularians, and aloricate protozoa were observed in a small number of individuals^13–16^. Further, analyses of fatty acids and lipids^17,18^ and stable isotopes^20–22^, indicate that leptocephali feed on POM originating from organisms at lower trophic levels^13–16,19–22^. These observations suggest that marine snow detrital-type particles in the POM are a food source^23^.

Next-generation sequencing (NGS) is increasingly being used to analyze stomach contents for a range of different organisms, as use of conventional visual methods poses unique challenges^24–26^. In the western North Pacific, high proportions of hydrozoans, copepods, coccidian parasites, tunicates, and fungi were detected by NGS analysis of gut contents from leptocephali belonging to five Anguilliform families, including the Japanese eel, in a study designed to evaluate the level of contamination from the external surface of the larval intestines^27^. In the Sargasso Sea, DNA sequence analysis of marine snow particles and the gut contents of European eel, *Anguilla anguilla*, larvae indicated a high abundance of hydrozoans, radiolaria, copepods, and dinoflagellates in both compositions^28,29^. Previous NGS studies in the Atlantic and Pacific oceans showed abundant hydrozoan sequences and included relatively similar eukaryotic compositions in the leptocephali gut contents.

To help understand the nature of materials consumed as food constituting the natural diet of Pacific eel larvae, the eukaryotic composition of gut contents was compared with POM, including the marine snow detrital-type particles in environmental seawater, using metagenomic analysis. The differences in the gut content compositions of eel larvae were examined by comparing eel taxa, groups with different body lengths, and eels from different geographical regions. Oceanographic observations were conducted in the oligotrophic tropical–subtropical western North Pacific areas, where feeding habits of eel larvae have been reported. The feeding environment of leptocephali was determined from the relationship observed between the compositions (gut content and POM of seawater) and marine environments. We surveyed a similar but wider area than that investigated in a previous study^27^ because leptocephali feed on marine snow materials that vary in composition, both spatially and temporally, depending on the plankton composition across locations and feeding times. Oceanographic observations were recorded at 63 stations, and water samples were collected from more than half of the stations. POM samples for DNA analysis were obtained from five water depths above 200 m at nine stations, and phytoplankton were observed under a microscope from the same stations and depths where POM was sampled. This study provides a new insight into the food sources and the feeding ecology of eel larvae in the oligotrophic tropical–subtropical western North Pacific Ocean.

## Results

### Anguilliformes leptocephali observed in this study

**Figure 1:**
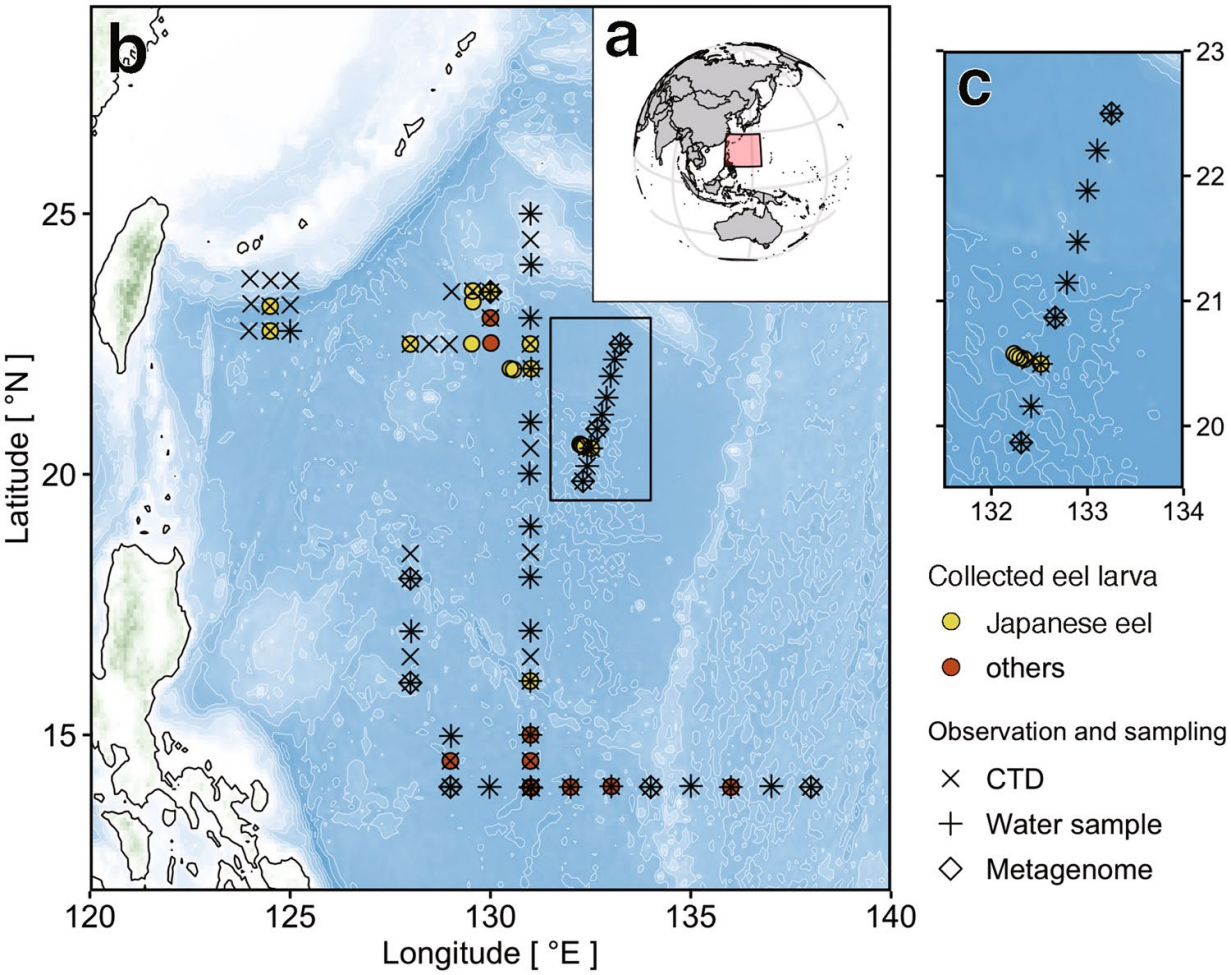
Sampling map. Overview map of the sampling and survey stations in the tropical and subtropical western North Pacific. The pink square indicates our research area on the global map (**a**). Enlarged views of the research area (**b**, **c**). Circles denote stations where eel larvae were collected (yellow circles: larvae of Japanese eel, red circles: other larvae). Cross (×), plus (+), and diamond (◇) indicate CTD observation, water sample collection points for analysis of marine environments and phytoplankton, and water sample collection points for metagenome analysis, respectively. Location data of all stations are available in Supplementary Table 1. The map was generated using the marmap package (version 1.0.3) in R with data imported from the NOAA server (accessed on 5 December 2019).

During a cruise to the western North Pacific between 28 September and 6 November 2016, 79 stations were visited across a latitude range of 14.0°N–26.6°N and a longitude range of 124.0°E–142.1°E (Fig. 1). Leptocephali were caught in net towing made to maximum depths of 80–221 m (Supplementary Table 2). Overall, 75 eel larvae belonging to four taxa were caught (Table 1; 36 Japanese eels, *A. japonica*; 13 giant mottled eels, *Anguilla marmorata*; 16 marine congrid eels, *Gnathophis* spp.; and 10 sawtooth eels, Serrivomeridae). Japanese eel larvae were found at 17 sampling stations and other Anguilliformes were found at 20 stations. Leptocephali were obtained from 29 stations (Fig. 1b,c). The length of all leptocephali was between 13.0–59.6 mm, with a mean length of 42.0 ± 11.8 mm. For Japanese eels, the length was between 41.9–59.6 mm, with a mean length of 50.4 ± 4.0 mm (Table 1).

**Table 1:** Total length of leptocephalus in four taxa. s.d. indicates standard deviation.

Taxon name	n	Min	Max	Mean	SD
*Anguilla japonica*	36	41.9	59.6	50.4	4.0
*Anguilla marmorata*	13	13.0	39.8	24.7	8.6
*Gnathophis* spp.	16	32.1	58.9	43.1	8.9
Serrivomeridae	10	22.2	41.0	32.6	6.8
Total	75	13.0	59.6	42.0	11.8

### Hydrographic measurements in subtropical/tropical western North Pacific

**Figure 2:**
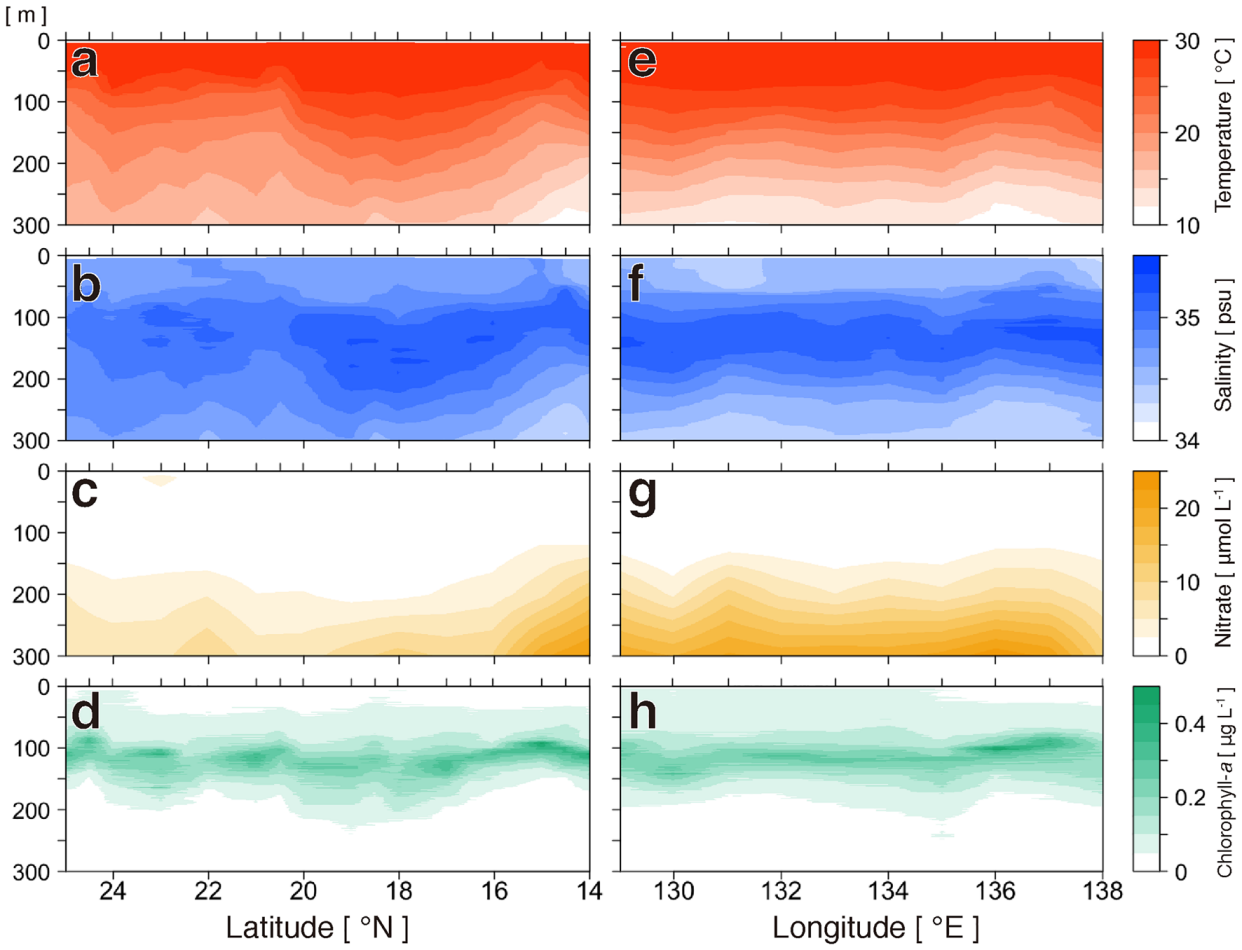
Oceanographic observation. Contour maps of four marine environments at a latitudinal line from 26.6°N to 14.0°N at 131°E (**a–d**) and at a longitudinal line from 129.0°E to 138.0°E at 14.0°N (**e–h**): temperature (**a**, **e**), salinity (**b**, **f**), nitrate concentration (**c**, **g**), and chlorophyll-*a* concentration (**d**, **h**).

**Figure 3:**
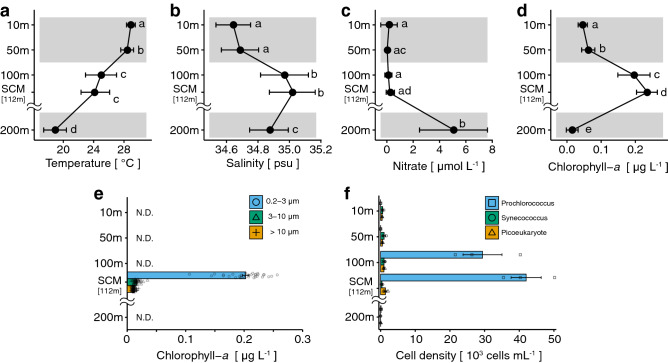
Vertical profiles of environmental factors. Line plots (**a–d**) generated using mean values (circles) of all data with standard deviations (horizontal lines) at five depth layers from all 63 (temperature and salinity) or 38 stations (nitrate and total chlorophyll-*a*). SCM refers to subsurface chlorophyll maximum. Gray shading in **a–d** shows three layers (shallow, 10 and 50 m; middle, 100 m and the SCM; and deep, 200 m) with different characteristics. Letters indicate significant differences in mean values (*p* < 0.01) (**a–d**). Chlorophyll-*a* concentrations of three filter size fractions (0.2–3, 3–10, and > 10 μm) at SCM depth from all 37 stations of total chlorophyll-*a* stations (**e**). Average cell densities of *Prochlorococcus*, *Synechococcus*, and picoeukaryotic phytoplankton (**f**) at five depth layers from all three stations. *Prochlorococcus*, *Synechococcus*, and picoeukaryotic phytoplankton did not show significant differences between the two layers (**f**). 112 m is the mean depth of the SCM. Error bars in **e** and **f** indicate standard errors of the mean. Data are also available in Supplementary Table 1.

Marine environments exhibited similar longitudinal (north and south line on 131.0°E, Fig. 2a–d) and latitudinal (east and west line on 14.0°N, Fig. 2e–h) patterns in the study area. The temperature decreased with depth (Fig. 2a,e). Maximum salinity (> 35 psu) was observed between 70 and 200 m in depth (Fig. 2b,f). The concentration of nutrients (nitrate) was depleted at depths shallower than 100 m (Fig. 2c,g). Vertical marine environments were compared using the mean values of the standard discrete depths in the survey area (10, 50, 100, and 200 m, and SCM; Fig. 3a–d) to describe the differences among three layers, namely 10–50 m, 100 m–SCM, and 200 m. The mean temperature was > 28 °C at 10–50 m, between 24 °C and 28 °C at 100 m–SCM, and < 20 °C at 200 m (Fig. 3a). The mean salinity was approximately 34.7 at 10–50 m, approximately 35.0 at 100 m–SCM, and approximately 34.8 at 200 m (Fig. 3b). Nitrate concentration was almost 0 μmol L^−1^ from 10 m to SCM and 5 μmol L^−1^ at 200 m (Fig. 3c). The concentration of Chl-*a* was < 0.1 μg L^−1^ at 10–50 m, > 0.2 μg L^−1^ at 100 m–SCM, and almost 0 μg L^−1^ at 200 m (Fig. 3d). Chlorophyll concentration per phytoplankton size in the SCM (three sizes: > 10 μm, large; 3–10 μm, medium; and 0.2–3 μm, small) was predominantly attributed to the presence of small phytoplankton (Fig. 3e), which almost exclusively consisted of *Prochlorococcus* (Fig. 3f). Thus, the 100 m–SCM layer was the boundary or the upper part of the thermocline and the pycnocline because marine environments changed rapidly upon descension from this layer. The marine environments in the area, which indicate the feeding ecology of leptocephali, generally showed similarities across different geographical areas (horizontally) but differed vertically among the three layers.

### NGS sequence composition of larval gut contents and POM

**Figure 4:**
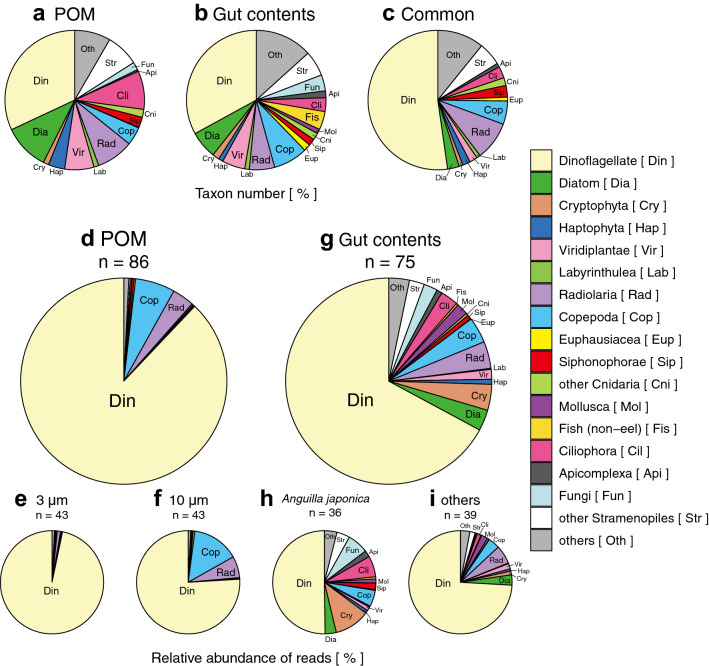
Eukaryotic composition of larval eel gut contents and POM of seawater. Overview of the number of eukaryotic taxa in POM (particulate organic matter) of 86 samples (**a**), eel larva gut contents (**b**), and common taxon number between POM and gut contents (**c**). A complete overview of the eukaryotic composition in 86 POM samples (**d**) and eel larva gut contents of 75 samples (**g**). Complete overview of the eukaryote composition in two size-filtered fractions of POM (**e**: 3–10 μm; **f**: > 10 μm; 43 samples each) and eel larva gut contents of 36 Japanese eel samples (**h**) and the other 39 anguillid eel larva samples (**i**).

The 18S rRNA gene sequences identified from the guts of 75 eel larvae from four taxa (Table 1) and the 86 filtered seawater samples (43 of > 10 μm and 43 of 3–10 μm) passed the quality controls. They encompassed 2,706,207 reads, representing 784 taxa across all samples, 707 taxa from POM of seawater, and 188 taxa from eel gut contents. There were 111 taxa that overlapped with those observed in the POM and gut contents. In POM, dinoflagellates were the most diverse (226 taxa) followed by diatoms (75 taxa), Ciliophora (63 taxa), radiolaria (61 taxa), Viridiplantae (48 taxa), copepods (30 taxa), and haptophytes (27 taxa). In Fig. 4, “other stramenopiles” (54 taxa) represents a small number of reads obtained from 15 classes, such as Chrysophyceae and Dictyochophyceae, and “others” (25 taxa) represents a small number of reads obtained from 13 taxonomic groups, such as Crustacea and Thaliacea (Fig. 4a). In the gut contents, dinoflagellates showed the highest number of taxa (62 taxa). Copepods and diatoms were represented by 15 and 12 taxa, respectively. However, other stramenopile and other taxa were not represented (Fig. 4b). Common taxa between the POM and gut contents were dominated by dinoflagellates (58 taxa), with contributions from radiolaria (10 taxa) and copepods (6 taxa). Additionally, other stramenopile and other taxa were not represented (Fig. 4c). Labyrinthulea and Siphonophorae taxa were detected in POM (8 and 17 taxa, respectively), gut contents (1 taxon and 3 taxa, respectively), and commonly observed taxa (2 and 3 taxa, respectively; Fig. 4a–c).

Next, we determined the percentage compositions of sequence reads. The primary component of POM of seawater (Fig. 4d) was dinoflagellates (89.3%) followed by copepods (6.2%) and radiolaria (3.5%). In small POM (3–10 μm, Fig. 4e), dinoflagellates (96.7%) dominated. Copepods (14.7%, chi-squared test: *p* < 0.01) and radiolaria (6.7%, *p* < 0.01) were relatively more abundant in large POM (> 10 μm in size, Fig. 4f) than in small POM (Fig. 4e). The primary components of the gut contents of leptocephali (Fig. 4g) were dinoflagellates (67.1%, *p* < 0.01), but their abundance in the gut contents was lower than that in POM (Fig. 4d). Additionally, phytoplankton excluding dinoflagellates [9.7%, comprising cryptophyta (4.1%), diatoms (3.3%), Viridiplantae (1.5%), and haptophyta (0.8%)] were more abundant than copepods (4.1%) and radiolaria (4.2%) in the gut contents. Moreover, Ciliophora (2.8%, *p* < 0.01), fungi (2.2%, *p* < 0.01), and cnidaria (1.1%, *p* < 0.01) found in the gut contents (Fig. 4d) were markedly rare in POM (Fig. 4d). Labyrinthulea and Siphonophorae were rare at < 1% in both POM and gut contents (Fig. 4d,g). At lower taxonomic levels, no prominent abundant taxa were observed (Table 2).

**Table 2:** Top 10 most abundant genera in NGS reads in gut contents and POM. Bold number indicates high abundance in POM or gut contents. “–” indicates no detection.

Taxonomic group	Family	Genus	Reads percent (%)	
			POM	Gut content
Dinoflagellate	Amoebophryaceae	*Amoebophrya*	**7.3**	**3.5**
	Blastodinidae	*Blastodinium*	**3.5**	1.1
	Brachidiniaceae	*Karenia*	**3.7**	1.1
	Dinophysiaceae	*Ichthyodinium*	1.7	**5.6**
	Duboscquellaceae	*Euduboscquella*	**6.5**	**6.6**
	Gymnodiniaceae	*Gymnodinium*	**4.5**	1.2
		*Gyrodinium*	**10.1**	**12.1**
		*Lepidodinium*	**9.4**	**1.6**
		*Nusuttodinium*	0.6	**5.4**
	Heterocapsaceae	*Heterocapsa*	0.8	**7.7**
	Kareniaceae	*Karlodinium*	**4.2**	**3.3**
	Prorocentraceae	*Prorocentrum*	**2.8**	1.4
	Warnowiaceae	*Warnowia*	**13.2**	1.2
Cryptophyta	Geminigeraceae	*Urgorri*	–	**2.2**
Ciliophora	Strobilidiidae	*Pelagostrobilidium*	0.004	**2.2**

The highest abundance of genera was exhibited by the dinoflagellate family. Gymnodiniaceae, and *Gyrodinium* spp. were commonly and predominantly observed in both POM and gut contents (Table 2). Moreover, almost all dinoflagellate genera were abundant in POM and gut contents. However, three genera (*Ichthyodinium*, *Nusuttodinium*, and *Heterocapsa*) were more abundant in gut contents than in POM. *Urgorri* (cryptophyte) and *Pelagostrobilidium* (ciliophoran) showed high abundance only in gut contents. The gut contents of Japanese eel larvae (Fig. 4g) exhibited a lower abundance of dinoflagellates (50.2%, *p* < 0.01) and radiolaria (0.4%, *p* < 0.01), but showed higher abundances of Cryptophyta (11.1%, *p* < 0.01), Ciliophora (6.6%, *p* < 0.01), fungi (6.6%, *p* < 0.01), and copepods (5.1%, *p* < 0.01) compared with other species of leptocephali (Fig. 4h). The components of the gut contents in Japanese eels (Fig. 4g) differed from those of other leptocephali, which consisted of dinoflagellates (74.1%), Cryptophyta (1.2%), radiolaria (5.8%), and copepods (3.6%) (Fig. 4f). However, the composition of the gut contents of leptocephali seemed to vary on an individual basis even within the same eel species (Supplementary Fig. 1).

**Figure 5:**
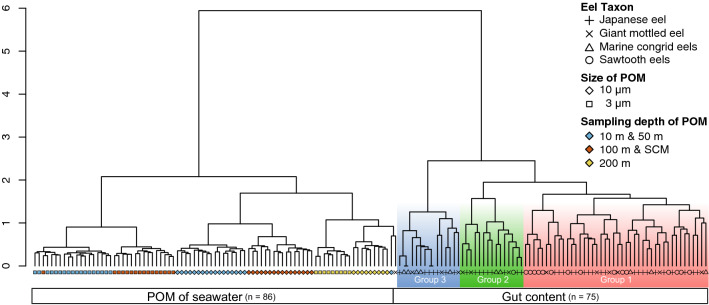
Cluster dendrogram based on eukaryotic compositions in eel gut contents and POM of seawater. POM samples classified into two sizes (10 μm, diamond ◇ and 3 μm, square □) and three sampling depths (10 m & 50 m: blue; 100 m & the SCM: red; 200 m: yellow). Based on the gut content samples, three well-defined cluster groups (Group 1: red, Group 2: green, Group 3: blue) were observed.

**Figure 6:**
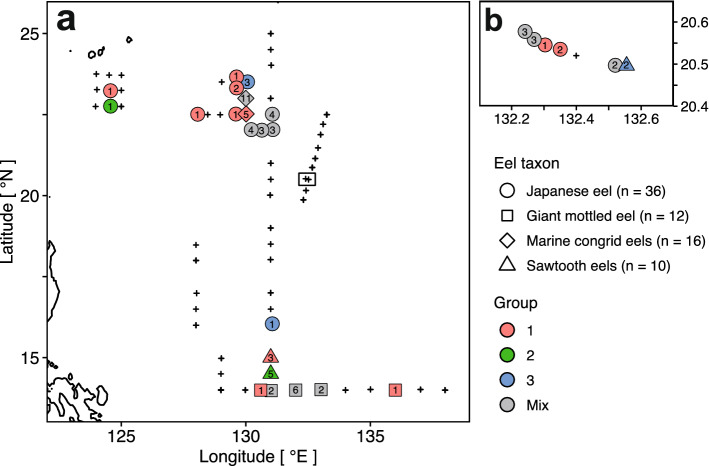
Distribution of four eel taxa within three cluster groups based on eukaryotic composition in gut content samples. Distribution of four eel taxa [circle ○: Japanese eel, square □: giant mottled eel (*Anguilla marmorata*), diamond ◇: marine congrid eels (*Gnathopis* spp.), and triangle △: sawtooth eels (*Serrivomeridae* spp.)]. Colors denote three cluster groups based on eukaryotic composition in the gut content samples in Fig. 5; gray indicates a mixture of multiple groups. Numbers on markers indicate the number of eel larvae. Note that a giant mottled eel was excluded from this map because it was not classified in the gut content cluster in Fig. 5.

**Figure 7:**
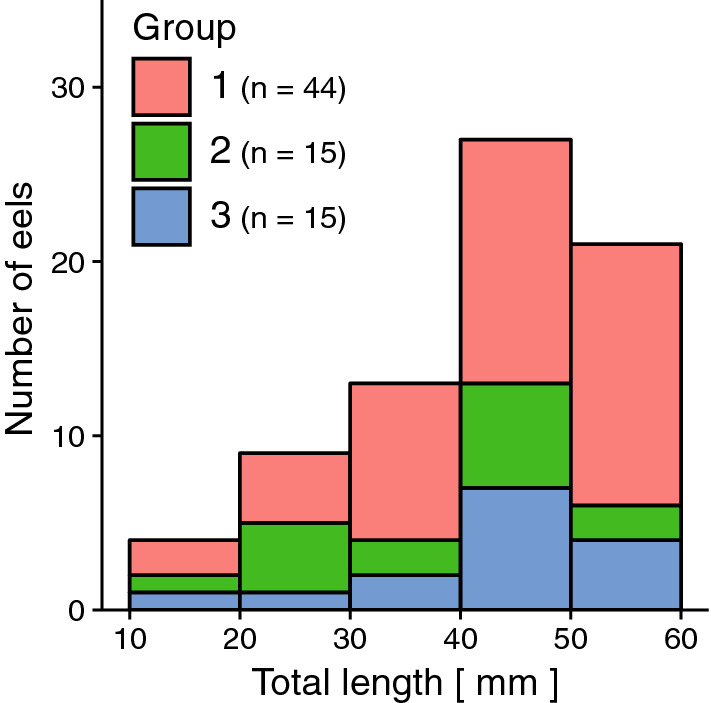
Histogram of eel larvae numbers at different size classes. Colors denote three cluster groups based on eukaryotic composition in the gut content samples in Fig. 5. Data are also available in Supplementary Table 1.

Based on genome components, gut contents clustered into one group and POM of seawater into another (Fig. 5). The gut contents could also be classified into three significantly different groups [Permutational multivariate analysis of variance (PERMANOVA): *r*^2^ = 0.23, *p* < 0.01]; however, these groups did not correspond to eel taxa. There was also no significant correlation between gut content composition and geographical distribution (Fig. 6a,b) (Mantel test: *r* =  − 0.013, *p* > 0.01). Gut content composition was not related to body length as the mean total length of leptocephali did not differ significantly among the groups (Fig. 7) (Wilcoxon rank-sum test: *p* > 0.01). The mean composition appeared to differ among the eel species (Fig. 4b,d); however, the differences among eel larvae individuals were greater than those observed among eel species (Fig. 5, Supplementary Fig. 1). The diet of eel larvae did not vary in relation to their growth level or distribution.

POM was characterized by the pore size of the filter and water depth (Fig. 5) as follows: (1) small (3–10 μm), 10–50 m and 100 m–SCM; (2) large (> 10 μm), 10–50 m and 100 m–SCM; and (3) small and large, 200 m. There was a significant difference in genome components among these groups (PERMANOVA: *r*^2^ = 0.23, *p* < 0.01) and the difference in composition corresponded to the findings obtained from oceanographic observations, which also changed across the same three layers (Fig. 3a–e). Regarding the composition of POM, it was considered that the vertical difference was greater than the horizontal difference because the clustered groups were not classified based on the sampling stations and the correlation between the composition and geographical distribution was not significant (Mantel test: *r* = 0.0057, *p* > 0.01).

### Vertical variation of POM and phytoplankton compositions at different depths

**Figure 8:**
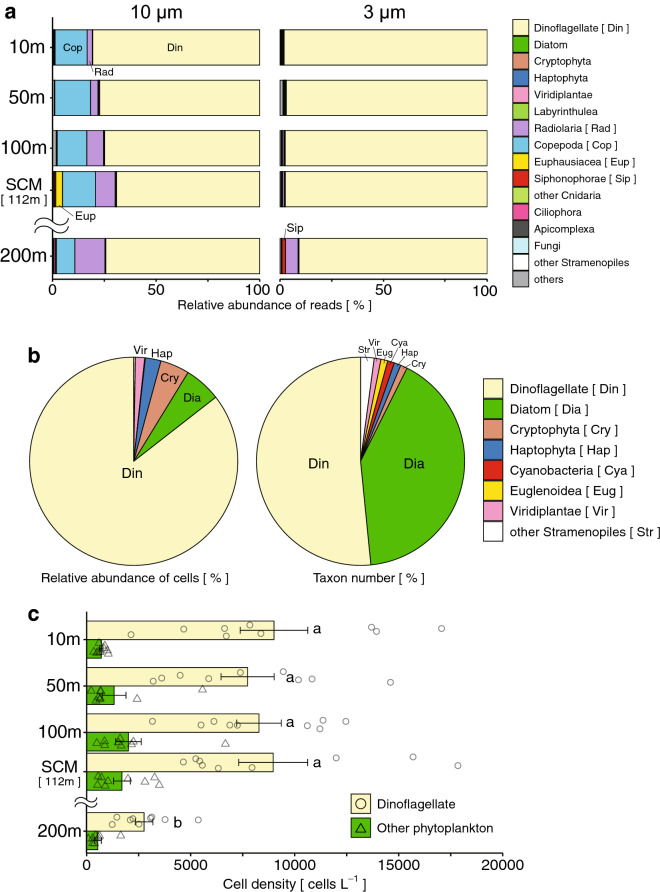
Eukaryotic composition in POM of seawater. Average relative abundances of eukaryotic reads from NGS analysis with two filter size groups (10 and 3 μm, respectively; 43 samples each) at five depth layers from nine stations (**a**). Overview of phytoplankton composition and numbers of taxa (> 2 µm) obtained by cell counting of 45 samples at the same five depth layers and same nine stations for NGS analysis of POM (**b**). Average phytoplankton composition at five depth layers in nine stations (**c**). Letters indicate significantly different mean values (*p* < 0.01) (**c**). Other phytoplankton composing of seven taxonomic groups excluding dinoflagellates did not show significant differences among the layers (**c**). The mean depth of the SCM is 112 m. Error bars in **c** indicate standard errors of the mean. Data are available in Supplementary Table 1.

The vertical distribution and feeding depth of the eel larvae in the present study are unknown. The feeding depth of leptocephali was estimated from the gut contents by comparing the gut contents to the composition of POM based on NGS analysis. POM can provide estimates of the water depth (Fig. 8a). First, the composition of POM was categorized by size and vertical differences. The community structure of five layers (10, 50, 100, and 200 m, and the SCM) showed a significant difference in both small (3–10 μm) and large POM (> 10 μm) (PERMANOVA: *r*^2^ > 0.2, *p* < 0.01). In small POM (3–10 μm), dinoflagellates constituted > 96% of the total and exhibited similar compositions at all five depths. The primary component of large POM (> 10 μm) was dinoflagellates at all five depths (74%–94%). However, the compositions of copepods and radiolaria, the next most predominant taxa, changed between the three layers. Specifically, the levels of these two taxa decreased in shallow layers (5.2% at 10 m, 4.7% at 50 m), increased in middle layers (16.3% at 100 m, 21.2% at SCM), and slightly decreased in the deep layer (15.4% at 200 m), especially for copepods, which showed a high abundance (> 10%) at 100 m and in the SCM. The composition of seawater in the middle layer resembled eel gut contents because of a relatively high abundance of taxa other than dinoflagellates (Fig. 4g).

Phytoplankton observed in POM by NGS was compared to the vertical profile of phytoplankton identified by light microscopy (Fig. 8b,c). The composition of phytoplankton (> 2 μm) estimated by cell counting showed a predominance of dinoflagellates (85.5%), with small contributions from diatoms (5.7%), cryptophytes (4.5%), and haptophytes (2.4%; Fig. 8b). Both dinoflagellates (49 taxa) and diatoms (39 taxa) displayed high diversity (Fig. 8b). The dominant taxon was represented by the family Gymnodiniaceae (Table 3), and the results were consistent with those of the NGS analysis (Table 2). However, Gymnodiniaceae may include many unidentified genera. This family comprises small members that may be classified at the genus level using light microscopy-based analysis. The major phytoplankton component in every depth layer was represented by dinoflagellates (Fig. 8c). The abundance of other phytoplankton peaked in the middle layer (100 m and the SCM; Fig. 8c). The compositions of POM and the cell count in the middle layer (100 m and SCM) corresponded to the composition of eel gut contents (Fig. 4g).

**Table 3:** Top 10 dominant phytoplankton taxa determined by light microscopy cell counting.

Taxinomic group	Class	Order	Family	Genus	Cell density (cells L^-1^)	(% )
Dinoflagellate	Dinophyceae	Gymnodiniales	Gymnodiniaceae	–	209,644	53.9
		Peridiniales	–	–	85,634	22.0
			Oxytoxaceae	*Oxytoxum*	19,573	5.0
			Peridiniaceae	*Scrippsiella*	4,421	1.1
		Prorocentrales	Prorocentraceae	*Prorocentrum*	5,793	1.5
Diatom	Bacillariophyceae	Naviculales	Naviculaceae	*Navicula*	3,531	0.9
		Bacillariales	Bacillariaceae	*Fragilariopsis*	3,295	0.8
Cryptophyta	Cryptophyceae	–	–	–	17,577	4.5
Haptophyta	Prymnesiophyceae	Phaeocystales	Phaeocystaceae	*Phaeocystis*	9,463	2.4
Viridiplantae	Pyramimonadophyceae	Pyramimonadales	Pyramimonadaceae	*Pyramimonas*	5,611	1.4

“–” Indicates an unidentified taxonomic level.

## Discussion

The gut contents of 75 eel leptocephali, as analyzed by 18S rRNA gene sequencing, showed that dinoflagellates were the most abundant genera in the study area region of the western North Pacific Ocean during the research cruise, with lesser contributions from other phytoplankton (cryptophyta and diatoms) and zooplankton (ciliophorans and copepods). Dinoflagellates were represented by many taxa, predominantly the family Gymnodiniaceae, as evidenced by DNA-based analysis of the gut contents and POM and light microscopy. Across tropical and subtropical regions of oceans including the Pacific Ocean, dinoflagellate blooms are reportedly sporadic^30^. Small dinoflagellate (< 20 μm) represented the most highly abundant phytoplankton as per the microscopic observations, and *Gyrodinium* spp. (belonging to Gymnodiniaceae and showing high abundance in the DNA analysis of our study) showed predominance among the dinoflagellate taxa^30^. Previous NGS studies in the Sargasso Sea and the western North Pacific Ocean also detected dinoflagellates in both the leptocephalus gut contents^27,29,31^ and the marine snow particles^31,32^. Therefore, it appears that dinoflagellates may have been an important food source for leptocephali in the survey area when our study was conducted.

Although there is currently no significant evidence indicating that Siphonophorae (belonging to the phylum Cnidaria) are an important food source for leptocephali, they were detected as one of the dominant taxa in the eukaryotic composition of the gut contents of leptocephali and the marine snow particles using DNA analysis in both the western North Pacific Ocean^27^ and the Sargasso Sea^29^ and have also been observed to be abundant in the marine environment^29^. However, the results of the present study showed a low abundance of siphonophores in both the gut contents and the POM of seawater, despite being in almost the same sea area and survey period reported by Chow et al^27^. The seasonal distribution and abundance of siphonophores are primarily attributed to fluctuations in environmental factors that control the sexual cycle^33,34^, and in a favorable environment, siphonophores can rapidly reproduce asexually^35–37^. In the western North Pacific Ocean, spatiotemporal variation in siphonophore assemblages is closely related to temperature, salinity, chlorophyll concentration, and zooplankton abundance, which are strongly influenced by ocean currents^38^ and seasonal monsoons^39^. For instance, in summer, the southwestern monsoon increases the species number and abundance of siphonophores, whereas in winter the northeast monsoon decreases species number and abundance^40^. Moreover, typhoons have an impact on the abundance of siphonophores, with a decrease observed immediately after the passage of a typhoon^38^; however, an increase has been observed one month later^41^.

In the western North Pacific Ocean, phytoplankton increase in abundance following typhoons because the cyclonic eddies generated by typhoons induce considerable ocean mixing and supply nutrients from the lower layers to the oligotrophic upper layers^42^. High abundances of phytoplankton following typhoons have been reported in tropical-subtropical areas^43–45^, and the strength and speed of typhoons determine the scale of phytoplankton blooms^46^. Increased phytoplankton abundances are maintained for up to 10 days after the passage of a typhoon^45,47,48^. Both this study (28 September—11 November 2016) and that reported by Chow et al.^27^ (29 September—13 October 2017) were conducted in autumn and seem to be weakly influenced by the monsoon. In 2017, typhoons did not occur during the survey period reported by Chow et al.^27^, with the last occurrence reported between 9 and 18 September at the survey area^49^. It is estimated that these meteorological conditions provide a favorable environment for siphonophores. In contrast, in 2016, six typhoons occurred in the survey area during the survey period, including two remarkably strong typhoons (typhoons Chaba and Haima)^49^. Therefore, this study presumes that the variation of marine environments caused by the disturbance of typhoons leads to a decrease in siphonophore abundance and an increase in phytoplankton population, and that this result would be reflected in the DNA compositions of the marine snow particles and gut contents of leptocephali.

The microscopic studies of leptocephalus gut contents that found the apparent presence of Labyrinthulomycetes and suggested that they might be a possible food source for the European eel and other eel larvae in the Sargasso Sea^50^ did not verify their presence in the gut contents genetically or by quantitative observations. Presently, there is also no evidence available on the dietary importance of protists. This study may not have accurately detected Labyrinthulomycetes because such studies require specific PCR primers for amplification of their 18S rDNA sequences^51^. Although they were detected in small numbers in POM and gut contents in this study, we may not have accurately detected species of importance of Labyrinthulomycetes as a food source for eel leptocephali in the western North Pacific.

Observations of gut contents and stable isotope studies have suggested that marine snow detrital-type particles are a food source, but this requires further validation. Data confirming the digestibility and absorbability of unprocessed zooplankton and/or phytoplankton (alive or shortly after death) by eel leptocephali are not available^52–54^. Artificially cultured Japanese eel survived by eating small POM (53 and 25 µm) from seawater^22^; however, they died upon eating large POM (> 350 µm), strongly suggesting that relatively large zooplankton and/or phytoplankton, specifically those with hard or sharp bodies, seriously damaged the digestive organs of leptocephali. Smaller particles may therefore be of importance for these larvae^16^, and this corresponds with the food source known as marine snow detrital-type particles^23^. Small spherical particles (2–40 µm in diameter) of marine snow comprising polysaccharides and proteins within the aggregate is a common finding in both the gut contents of anguilliform leptocephali^15,16,23,55^ and environmental water^16^, suggesting that marine snow particles originating from phytoplankton and cyanobacteria are a food source for the leptocephali^16^. Thus, food sources of eel larvae in the marine environment are assumed to be small or soft marine snow particles in POM^56^. In this study, the comparison of gut contents and POM indicated several differences, especially in dominant genera. The existence of highly abundant genera only in gut contents strongly suggests that leptocephalus feeds selectively on specific marine snow detrital-type particles, which can, such as in the present study, predominantly contain dinoflagellates and soft organisms (e.g. cryptophyte and ciliophoran). The forward-pointing teeth are well-designed to help squeeze particles into the oral cavity^14,57^ and also facilitate the expulsion of excess material away from the mouth to avoid fouling of the teeth^58^. The mouth structure of eel larvae, which is suitable for the capture of small particles, supports the hypothesis of a selective marine snow diet^16^. Current findings suggest the importance of marine snow particles that are a mixture of principal dinoflagellates and other supplementary phytoplankton and zooplankton materials^13,14^. Additionally, *Prochlorococcus* may be important as a food source because they are present at high levels in the SCM. The cell membranes of *Prochlorococcus* are covered with proteins^59^ and release monosaccharides^60^, the extracellular exudates are composed of low-molecular-weight compounds^61^, and the extracellular vesicles contain rich nutrients such as lipids, proteins, and nucleic acids^62,63^. In seawater, it is known that transparent exopolymer particles (TEPs) exist and are mixed with a substantial amount of protein-rich amorphous particles^64–66^. Thus, leptocephali likely feed on the nutrient-rich soft marine snow particles composed of substances derived from various organisms, which have been decomposed by bacteria^67^. Our results are consistent with the low trophic position of the natural food of leptocephali estimated by stable isotopic analysis of the nitrogen located between primary producers and primary consumers^23^.

The compositions of eel gut contents did not completely match that of the POM of seawater. The organismal composition of POM in the marine environment differed from that of marine snow detrital-type particles that were assumed to be the true food source for leptocephali. This may be explained by the difference that is caused by selective feeding on soft and small particles of marine snow. It may also be explained by the eukaryotic phytoplankton composition. Eukaryotic phytoplankton, excluding dinoflagellates, are mainly composed of diatoms, cryptophytes, and haptophytes. This second most abundant group of organisms in the gut contents was rarely observed in POM, although it was present at a relatively high abundance (ca. 15%) in total cell density at the middle layer (100 m and SCM), as revealed by microscopic observation. These phytoplankton might be underestimated in the metagenomic analysis due to relatively low copy numbers of ribosomal RNA compared to dinoflagellates^68–70^. Nevertheless, dinoflagellates exhibited the highest abundance in both the POM and gut contents of leptocephali. Dinoflagellates may be one of the most important food sources for eel larvae in the western North Pacific Ocean.

Oceanographic observations showed that the marine environments in which the leptocephali were distributed exhibited similar patterns horizontally but differed vertically among the following three layers: 10–50 m, 100 m–SCM, and 200 m. SCM was present at a depth of 82–140 m throughout our study area and it was observed at the upper part of the thermocline corresponding to about 24 °C. These oceanological features were also supported by previous studies in the tropical and subtropical western Pacific Ocean^12,71^. Under rearing conditions, the optimal temperature for the early development of larvae^72,73^ and the most suitable temperature to ensure a lack of deformities^74^ in Japanese eel was reported to be 24–28 °C. The relationship between water temperature and otolith diameter for cultured Japanese eel larvae suggests that the mean ambient water temperature experienced by natural larvae during their first month after hatching is approximately 23°C^72^. The depth at which Japanese eel preleptocephali (eel larvae at an early developmental stage) were distributed was estimated to be at the SCM (~ 150 m in depth) and the upper part of the thermocline (temperatures of 25.5–27.3 °C), based on the collection data and the oxygen stable isotope ratios in otolith aragonite of the preleptocephali^75,76^. The specific gravities of Japanese eel larvae during their early feeding period were close to those of seawater at a subsurface depth of 130 m in the North Equatorial Current region, such that their buoyancy would enable them to easily adjust their depth to remain in the layer with the maximum food availability^5^.

POM, including the marine snow detrital-type particles hypothesized to be the food source of leptocephali, is produced in the upper few hundred meters, including within the SCM^77,78^; it may often accumulate around the pycnocline^79^. A high abundance of POM has been identified in the subsurface layer (100–200 m) in the subtropical western North Pacific Ocean^16,22^. A comparison of leptocephalus gut contents and the marine snow particles in seawater using microscopic observations supports the hypothesis that leptocephali may efficiently feed on marine snow particles within the SCM^16^. The results of the metagenomic analysis in this study showed that the composition of seawater differed with water depth, and the composition of the SCM was similar to that of the gut contents of eel larvae. Our results strongly suggest that eel larvae feed on food within the SCM. Conversely, no clear geographical differences were observed in the composition of either leptocephalus gut contents or POM of the seawater samples. The marine snow particles in POM at the SCM that are the diet of leptocephali may be extensively distributed in a uniform manner across the entirety of the tropical and subtropical western North Pacific. The SCM contains abundant dinoflagellates, and zooplankton and phytoplankton are major components of the marine snow particles; therefore, marine snow particles may sufficiently support the growth of eel larvae. Thus, the chlorophyll maximum at the thermocline may be advantageous for both the development and diet of eel larvae.

We estimated the dietary components of several eel larvae species and the marine environment in the western North Pacific. These results provide novel information that may aid in protecting and improving the natural resources of the critically endangered Japanese eel and may lead to an improvement in its early survival during aquaculture. In future research, we hope to clarify the actual food material consumed by eel larvae in the marine environment by biogeochemical analyses and to determine the manner in which the diet of leptocephali is formed through bio-chemical-physical processes in the oligotrophic ocean.

## Methods

### Ethics statement

The larval samples captured with plankton nets deployed from the research vessels were dead on retrieval and sampled at this juncture. All plankton net operations were conducted in high seas outside the exclusive economic zone. Therefore, the approval of coastal states was not required under the United Nations Convention on the Law of the Sea (UNCLOS).

### Oceanographic observations and collection of water samples

Measurements of conductivity-temperature-depth (CTD) and chlorophyll fluorescence were conducted at 63 stations, in which a CTD instrument attached to a sampling rosette containing Niskin bottles was lowered to 1,000 m. We used data from temperature and salinity measured at 1-m intervals by CTD observation. SCM depth was determined on the ship by noting the vertical profile of the chlorophyll fluorescence. Water samples for the analysis of nutrient concentrations were collected at 38 stations using Niskin bottles at 12 standard discrete depths from 0 to 1,000 m (Supplementary Table 1). The total chlorophyll concentrations were analyzed at the same 38 stations as nutrients; however, samples were collected at six water depths shallower than 200 m (0, 10, 50, 100, and 200 m, and the SCM), and the size-fractionated chlorophyll concentration (10, 3 and 0.2 μm) was measured only at the SCM depth at 37 stations (Supplementary Table 1). Seawater samples of POM for metagenomic analysis were obtained at nine stations at five standard discrete depths (10, 50, 100, and 200 m, and the SCM). In total, 1 L of seawater was fractioned using 10-µm and 3-µm pore size nucleopore filters of 42 mm in diameter. After filtration, these filters were immediately stored in 1.5-mL Eppendorf tubes at − 60 °C and transferred to the laboratory. For evaluating large phytoplankton, 1-L seawater samples from nine stations at five depths (same as those used for metagenomic analysis) were immediately fixed with acid Lugol solution (final concentration of 4%) and stored at 4 °C until analysis. Samples were concentrated by reverse filtration through a 2-μm nucleopore filter. In the concentrated samples (> 2 μm), phytoplankton were identified at the species level where possible following the methods prescribed by Tomas and Hasle^80^, and the phytoplankton cell densities (cells L^−1^) were estimated under a light microscope. Samples used for estimation of small phytoplankton were obtained at the same standard discrete depths from three stations where large phytoplankton analysis was performed (Supplementary Table 1). For small phytoplankton, water samples (1.5 mL) from three stations were fixed with paraformaldehyde (final concentration of 0.2%) for 5–10 min, frozen in liquid nitrogen, and counted on a flow cytometer (FCM) equipped with a laser (excitation: 405 and 488 nm, standard filter set, NoyoCyte; ACEA Biosciences) following the reported protocol^81^. The cells that possessed Chl-*a* without phycoerythrin (PE) were counted as picoeukaryotes, those that possessed both Chl-*a* and PE < 2 μm in size were counted as *Synechococcus*, and those that possessed Divinyl Chl-*a* without PE were counted as *Prochlorococcus*.

### Sampling and identification procedures for eel larva

An Isaacs–Kidd Midwater Trawl (IKMT) net (8.7 m^2^ opening, 13 m long, 0.5 mm mesh, and canvas-made cod-end) was used to collect eel leptocephali. Oblique tows from a depth of 200 m were performed at night in the western North Pacific on 29 September and 6 November 2016. Leptocephali were sorted and placed on a chilled Petri dish. A total of 75 leptocephali (Japanese eel, *A. japonica*, 36; giant mottled eel, *A. marmorata*, 13; marine congrid eels, *Gnathophis* spp., 16; sawtooth eels, Serrivomeridae, 10) gut contents were selected for analysis (Table 1 and details in Supplementary Table 2). The body surface was rinsed several times using sterilized and refrigerated seawater and the rinsed leptocephali were then placed on a sterilized Petri dish. The leptocephali were then immersed in sterilized and refrigerated seawater, and their gut contents were squeezed out using an inoculating loop and pipette. The gut contents were placed in separate 1.5-mL Eppendorf tubes, kept at − 60 °C, and transferred to the laboratory.

### DNA extraction, library preparation, and MiSeq sequencing

DNA was extracted from the guts of eel larvae and the filter samples of POM using the QuickGene DNA Tissue Kit S (KURABO, Osaka, Japan) and the 5% Chelex buffer method^82^, respectively. To perform metagenomic analysis using the MiSeq 300PE platform (Illumina, San Diego, California, USA), a set of universal primers to amplify the V7–9 hypervariable regions of the 18S-rRNA gene were used^83,84^. Parallel paired-end sequencing on the MiSeq platform requires PCR amplicons to be flanked by the following: (i) primer-binding sites for sequencing; (ii) dual-index (i.e., barcode) sequences; and (iii) adapter sequences for binding to the flow cells of the MiSeq.

We employed a two-step PCR approach to construct the paired-end libraries^85^. First and second-round PCRs were conducted following Dzhembekova et al.^84,85^. A PhiX DNA spike-in control was mixed with the pooled DNA library to improve the data quality of low-diversity samples, such as single PCR amplicons^85^. DNA concentrations of the pooled library and the PhiX DNA were adjusted to 4 nM using the buffer EB (10 mM Tris–HCl, pH 8.5) mixed at a ratio of 7:3.5 μL^85^. The 4-nM library was denatured with 5 μL of fresh 0.1 N NaOH^85^. Using the HT1 buffer (provided with the Illumina MiSeq v. 2 Reagent kit for 2 × 150 bp PE), the denatured library (10 μL; 2 nM) was diluted to a final concentration of 12 pM for sequencing on the MiSeq platform^85^.

### Treatment processes and operational taxonomic unit picking

Nucleotide sequences were demultiplexed depending on the 5′-multiplex identifier (MID) tag and primer sequences using the default format in MiSeq. The sequences containing palindromic clips longer than 30 bp and homopolymers longer than 9 bp were trimmed from the sequences at both ends. The 3′ tails with an average quality score of less than 30 at the end of the last 25-bp window were also trimmed from each sequence. The 5′ and 3′ tails with an average quality score of less than 20 at the end of the last window were also trimmed from each sequence. Sequences longer than 250 bp were truncated to 250 bp by trimming the 3′ tails. Trimmed sequences shorter than 200 bp were filtered out. Demultiplexing and trimming were performed using Trimmomatic version 0.35^86^ (http://www.usadellab.org/cms/?page=trimmomatic). The remaining sequences were merged into paired reads using Usearch version 8.0.1517 (http://www.drive5.com/usearch/). Further, singletons were removed and sequences were then aligned using Clustal Omega v 1.2.0. (http://www.clustal.org/omega/). Multiple sequences were aligned with each other, and data of the sequences that showed > 75% similarity in read positions were extracted. Filtering and a part of the multiple alignment process were performed using the screen.seqs and filter.seqs commands in Mothur, as described in the Miseq SOP^87^ (http://www.mothur.org.). Erroneous and chimeric sequences were detected and removed using the pre.cluster (diffs = 4) and chimera.uchime (minh = 0.1^88^; http://drive5.com/usearch/manual/uchime_algo.html) commands in Mothur, respectively. Using the unique.seqs command of Mothur, the same sequences were collected into operational taxonomic units (OTUs). The contig sequences were counted as OTUs by count.seqs and used for the subsequent taxonomic identification analysis. Demultiplexed, filtered, but untrimmed sequence data were deposited in the DDBJ Sequence Read Archive under accession no. PRJDB8891.

### Taxonomic identification of the OTUs

Selected OTUs were then taxonomically identified. A subset of nucleotide databases comprising sequences that satisfied the chosen conditions (described below) were prepared for a BLAST search using the nucleotide (nt) database. One keyword was selected from among “ribosomal,” “rrna,” and “rdna,” but “protein” was not included in the title. For the taxonomy search, keywords such as “metagenome,” “uncultured,” and “environmental” were not included. Sequences from GenBank IDs retrieved from the nt database and downloaded from the NCBI FTP server were extracted on 22 March 2019 and used to construct a template sequence database. Subsequently, taxonomic identification of each OTU was performed using a BLAST search^89^. The BLAST search was conducted using NCBI BLAST + 2.2.30 + ^90^ with the default parameters, the same nucleotide subset as described above for the database, and all OTU-representative sequences as the query. Taxonomic information was obtained from the BLAST hit with top bitscores for each query sequence, and then the OTUs of the same top hit were merged. The removal of sequences containing errors was imperfect after the successive MPS data treatment processes. Sequences containing different types of errors derived from the original ones remained in the following analytical steps. Therefore, these sequences were detected as unique OTUs with the same blast top hit name but different similarities. To avoid overestimation of the OTUs, these artificial OTUs were merged into a single OTU with the greatest similarity score. Among the sequences detected from all samples, we excluded the sequences of eels and *Homo sapiens*. Finally, 784 taxa within the taxonomic categories of kingdom, phylum or division, class, order, family, genus, and species were determined by referring to WoRMS^91^.

### Statistical analysis

Statistical analyses were conducted using R^92^. Kruskal–Wallis rank-sum tests as analyses of variance were used to determine significant differences in the mean values of five environmental factors at five standard discrete depths. Then, Wilcoxon rank-sum tests with pairwise comparisons using Bonferroni-adjusted *p*-values were used as post hoc tests between every two layers. In between the two eukaryotic community compositions (e.g., gut content vs POM of seawater), a two-sample test for equality of proportions was statistically evaluated using the chi-square test without continuity correction using the stats package^92^. The variability in the eukaryotic community structure was examined using the Ward hierarchy clustering method based on Bray–Curtis dissimilarity^93^. Before estimating the dissimilarity matrix, the community structure was transformed into presence or absence data. PERMANOVA (permutations = 10,000) was used to statistically test the differences in the community composition among clusters or water depths based on the dissimilarity matrix^94^. The relationships between the community structure and geographical distribution were statistically evaluated using the Mantel test (permutations = 10,000, with Pearson’s correlation coefficient), which is a permutation test to determine the correlation between two dissimilar distance matrices (community vs. latitude–longitude). Cluster analysis, PERMANOVA, and the Mantel test were performed using the vegan package^95^. Differences in the mean values of each pair of leptocephalus length clusters were determined using Wilcoxon’s rank-sum test using the stats package^92^. *p*-values < 0.01 were considered significant.

## Supplementary Information

Supplementary Information

## References

[1] Tsukamoto K (1992). Discovery of the spawning area for Japanese eel. Nature.

[2] Tsukamoto K (2006). Spawning of eels near a seamount. Nature.

[3] Chow S (2009). Discovery of mature freshwater eels in the open ocean. Fish. Sci..

[4] Kurogi H (2011). First capture of post-spawning female of the Japanese eel *Anguilla japonica* at the southern West Mariana Ridge. Fish. Sci..

[5] Tsukamoto K (2009). Positive buoyancy in eel leptocephali: an adaptation for life in the ocean surface layer. Mar. Biol..

[6] Cheng PW, Tzeng WN (1996). Timing of metamorphosis and estuarine arrival across the dispersal range of the Japanese eel *Anguilla japonica*. Mar. Ecol. Prog. Ser..

[7] Chen JZ, Huang SL, Han YS (2014). Impact of long-term habitat loss on the Japanese eel *Anguilla japonica*. Estuar. Coast. Shelf Sci..

[8] Tanaka E (2014). Stock assessment of Japanese eels using Japanese abundance indices. Fish. Sci..

[9] Jacoby, D. & Gollock, M. *Anguilla anguilla*. The IUCN red list of threatened species, version 2014.2. *IUCN 2014* e.T60344A45833138. 10.1108/ICS-04-2017-0025 (2014).

[10] Onda H (2017). Vertical distribution and assemblage structure of leptocephali in the North Equatorial Current region of the western Pacific. Mar. Ecol. Prog. Ser..

[11] Saijo Y, Iizuka S, Asaoka O (1969). Chlorophyll maxima in Kuroshio and adjacent area. Mar. Biol..

[12] Furuya K (1990). Subsurface chlorophyll maximum in the tropical and subtropical western Pacific Ocean: Vertical profiles of phytoplankton biomass and its relationship with chlorophylla and particulate organic carbon. Mar. Biol..

[13] Otake T, Nogami K, Maruyama K (1993). Dissolved and particulate organic matter as possible food sources for eel leptocephali. Mar. Ecol. Prog. Ser..

[14] Mochioka N, Iwamizu M (1996). Diet of anguilloid larvae: Leptocephali feed selectively on larvacean houses and fecal pellets. Mar. Biol..

[15] Miller MJ, Otake T, Aoyama J (2012). Observations of gut contents of leptocephali in the North Equatorial current and Tomini Bay Indonesia. Coast. Mar. Sci..

[16] Tomoda T (2018). Observations of gut contents of anguilliform leptocephali collected in the western North Pacific. Nippon Suisan Gakkaishi.

[17] Deibel D, Parrish CC, Grønkjær P, Munk P, GisselNielsen T (2012). Lipid class and fatty acid content of the leptocephalus larva of tropical eels. Lipids.

[18] Liénart C (2016). Geographic variation in stable isotopic and fatty acid composition of anguilliform leptocephali and particulate organic matter in the South Pacific. Mar. Ecol. Prog. Ser..

[19] Miller MJ (2013). A low trophic position of Japanese eel larvae indicates feeding on marine snow. Biol. Lett..

[20] Miyazaki S (2011). Stable isotope analysis of two species of anguilliform leptocephali (*Anguilla japonica* and *Ariosoma major*) relative to their feeding depth in the North Equatorial Current region. Mar. Biol..

[21] Chow S (2010). Japanese eel *Anguilla japonica* do not assimilate nutrition during the oceanic spawning migration: evidence from stable isotope analysis. Mar. Ecol. Prog. Ser..

[22] Chow S (2017). Onboard rearing attempts for the Japanese eel leptocephali using POM-enriched water collected in the Western North Pacific. Aquat. Living Resour..

[23] Miller MJ, Hanel R, Feunteun E, Tsukamoto K (2020). The food source of Sargasso Sea leptocephali. Mar. Biol..

[24] Pompanon F (2012). Who is eating what: Diet assessment using next generation sequencing. Mol. Ecol..

[25] Wang M, Jeffs AG (2014). Nutritional composition of potential zooplankton prey of spiny lobster larvae: a review. Rev. Aquac..

[26] Ho TW, Hwang JS, Cheung MK, Kwan HS, Wong CK (2015). Dietary analysis on the shallow-water hydrothermal vent crab *Xenograpsus testudinatus* using Illumina sequencing. Mar. Biol..

[27] Chow S (2019). Molecular diet analysis of Anguilliformes leptocephalus larvae collected in the western North Pacific. PLoS ONE.

[28] Riemann L (2010). Qualitative assessment of the diet of European eel larvae in the Sargasso Sea resolved by DNA barcoding. Biol. Lett..

[29] Ayala DJ (2018). Gelatinous plankton is important in the diet of European eel (*Anguilla anguilla*) larvae in the Sargasso Sea. Sci. Rep..

[30] Estrada M (2016). Phytoplankton across tropical and subtropical regions of the Atlantic Indian and Pacific Oceans. PLoS ONE.

[31] Lundgreen RBC (2019). Eukaryotic and cyanobacterial communities associated with marine snow particles in the oligotrophic Sargasso Sea. Sci. Rep..

[32] Ayala D, Riemann L, Munk P (2016). Species composition and diversity of fish larvae in the Subtropical Convergence Zone of the Sargasso Sea from morphology and DNA barcoding. Fish. Oceanogr..

[33] Arai MN (1992). Active and passive factors affecting aggregations of hydromedusae: a review. Sci. Mar..

[34] Boero F (2008). Gelatinous plankton: Irregularities rule the world (sometimes). Mar. Ecol. Prog. Ser..

[35] Purcell JE (1982). Feeding and growth of the siphonophore *Muggiaea atlantica* (Cunningham 1893). J. Exp. Mar. Bio. Ecol..

[36] Alldredge, A. Particle aggregation dynamics. In *Encyclopedia of Ocean Sciences*, 2nd edn, 330–337 (Elsevier Inc., 2008). 10.1016/B978-012374473-9.00468-9

[37] Hosia A, Bamstedt U (2008). Seasonal abundance and vertical distribution of siphonophores in western Norwegian fjords. J. Plankton Res..

[38] Lo WT, Yu SF, Hsieh HY (2013). Effects of summer mesoscale hydrographic features on epipelagic siphonophore assemblages in the surrounding waters of Taiwan, western North Pacific Ocean. J. Oceanogr..

[39] Lo W-T, Yu S-F, Hsieh H-Y (2014). Hydrographic processes driven by seasonal monsoon system affect siphonophore assemblages in tropical-subtropical waters (Western North Pacific Ocean). PLoS ONE.

[40] Li KZ, Yin JQ, Huang LM, Song XY (2012). Comparison of siphonophore distributions during the southwest and northeast monsoons on the northwest continental shelf of the South China Sea. J. Plankton Res..

[41] López-López L, Molinero JC, Tseng L-C, Chen Q-C, Hwang J-S (2013). Seasonal variability of the gelatinous carnivore zooplankton community in Northern Taiwan. J. Plankton Res..

[42] Price JF (1981). Upper ocean response to a hurricane. J. Phys. Ocean..

[43] Toratani, M. Primary production enhancement by typhoon Ketsana in 2003 in western North Pacific. In *Remote Sensing of Inland, Coastal, and Oceanic Waters* (eds. Frouin, R. J. et al.) **7150**, 715013 (SPIE, 2008).

[44] Lin II (2012). Typhoon-induced phytoplankton blooms and primary productivity increase in the western North Pacific subtropical ocean. J. Geophys. Res. Ocean..

[45] Ishida H, Furusawa K, Makino T, Ishizaka J, Watanabe Y (2016). The effect of typhoons on phytoplankton communities and settling particle flux in the western North Pacific subtropical region. Oceanogr. Jpn..

[46] Siswanto E, Ishizaka J, Yokouchi K, Tanaka K, Tan CK (2007). Estimation of interannual and interdecadal variations of typhoon-induced primary production: a case study for the outer shelf of the East China Sea. Geophys. Res. Lett..

[47] Chen YLL, Houng-Yung C, Jan S, Tuo SH (2009). Phytoplankton productivity enhancement and assemblage change in the upstream Kuroshio after typhoons. Mar. Ecol. Prog. Ser..

[48] Tsuchiya K (2015). Typhoon-induced response of phytoplankton and bacteria in temperate coastal waters. Estuar. Coast. Shelf Sci..

[49] Typhoon information. Japan Meteorological Agency. https://www.data.jma.go.jp/fcd/yoho/typhoon/index.html. Accessed 10 Dec 2020.

[50] Miller MJ (2019). Morphology and gut contents of anguillid and marine eel larvae in the Sargasso Sea. Zool. Anz..

[51] Singh P, Liu Y, Li L, Wang G (2014). Ecological dynamics and biotechnological implications of thraustochytrids from marine habitats. Appl. Microbiol. Biotechnol..

[52] Tanaka H, Kagawa H, Ohta H, Unuma T, Nomura K (2003). The first production of glass eel in captivity: fish reproductive physiology facilitates great progress in aquaculture. Fish Physiol. Biochem..

[53] Stenly W (2013). Ingestion by Japanese eel *Anguilla japonica* larvae on various minute zooplanktons. Aquac. Sci..

[54] Butts IAE, Sørensen SR, Politis SN, Tomkiewicz J (2016). First-feeding by European eel larvae: a step towards closing the life cycle in captivity. Aquaculture.

[55] Tsukamoto K, Miller MJ (2020). The mysterious feeding ecology of leptocephali: a unique strategy of consuming marine snow materials. Fish. Sci..

[56] Bouilliart M, Tomkiewicz J, Lauesen P, De Kegel B, Adriaens D (2015). Musculoskeletal anatomy and feeding performance of pre-feeding engyodontic larvae of the European eel (*Anguilla anguilla*). J. Anat..

[57] Westeberg H (1990). A proposal regarding the source of nutrition of leptocephalus larvae. Int. Rev. Hydrobiol. Hydrogr..

[58] Miller M (2009). Ecology of anguilliform leptocephali: remarkable transparent fish larvae of the ocean surface layer. Aqua-BioScience Monogr..

[59] Strom S, Bright K, Fredrickson K, Brahamsha B (2017). The *Synechococcus* cell surface protein SwmA increases vulnerability to predation by flagellates and ciliates. Limnol. Oceanogr..

[60] Benner R, Kaiser K (2003). Abundance of amino sugars and peptidoglycan in marine particulate and dissolved organic matter. Limnol. Oceanogr..

[61] Seymour J, Ahmed T, Durham W, Stocker R (2010). Chemotactic response of marine bacteria to the extracellular products of Synechococcus and Prochlorococcus. Aquat. Microb. Ecol..

[62] Biller SJ (2014). Bacterial vesicles in marine ecosystems. Science (80-).

[63] Scanlan D (2014). Bacterial vesicles in the ocean. Science.

[64] Cisternas-Novoa C, Lee C, Engel A (2015). Transparent exopolymer particles (TEP) and Coomassie stainable particles (CSP): Differences between their origin and vertical distributions in the ocean. Mar. Chem..

[65] Long RA, Azam F (1996). Abundant protein-containing particles in the sea. Aquat. Microb. Ecol..

[66] Tanoue E, Ishii M, Midorikawa T (1996). Discrete dissolved and particulate proteins in oceanic waters. Limnol. Oceanogr..

[67] Simon M, Alldredge AL, Azam F (1990). Bacterial carbon dynamics on marine snow. Mar. Ecol. Prog. Ser..

[68] Godhe A (2008). Quantification of diatom and dinoflagellate biomasses in coastal marine seawater samples by real-time PCR. Appl. Environ. Microbiol..

[69] Zhu F, Massana R, Not F, Marie D, Vaulot D (2005). Mapping of picoeucaryotes in marine ecosystems with quantitative PCR of the 18S rRNA gene. FEMS Microbiol. Ecol..

[70] Gong W, Marchetti A (2019). Estimation of 18S gene copy number in marine eukaryotic plankton using a next-generation sequencing approach. Front. Mar. Sci..

[71] Furuya K, Marumo R (1983). The structure of the phytoplankton community in the subsurface chlorophyll maxima in the western North Pacific Ocean. J. Plankton Res..

[72] Kuroki M, Okamura A, Yamada Y, Hayasaka S, Tsukamoto K (2019). Evaluation of optimum temperature for the early larval growth of Japanese eel in captivity. Fish. Sci..

[73] Okamura A (2007). Effects of water temperature on early development of Japanese eel *Anguilla japonica*. Fish. Sci..

[74] Kurokawa T (2008). Influence of water temperature on morphological deformities in cultured larvae of Japanese eel, *Anguilla japonica*, at completion of yolk resorption. J. World Aquac. Soc..

[75] Tsukamoto K (2011). Oceanic spawning ecology of freshwater eels in the western North Pacific. Nat. Commun..

[76] Shirai K (2018). Temperature and depth distribution of Japanese eel eggs estimated using otolith oxygen stable isotopes. Geochim. Cosmochim. Acta.

[77] Ichikawa T (1982). Particulate organic carbon and nitrogen in the adjacent seas of the Pacific Ocean. Mar. Biol..

[78] Hebel DV, Karl DM (2001). Seasonal, interannual and decadal variations in particulate matter concentrations and composition in the subtropical North Pacific Ocean. Deep Sea Res Part II Top. Stud. Oceanogr..

[79] MacIntyre S, Alldredge AL, Gotschalk CC (1995). Accumulation of marines now at density discontinuities in the water column. Limnol. Oceanogr..

[80] Tomas CR, Hasle GR (1997). Identifying Marine Phytoplankton.

[81] Suzuki K (2005). Responses of phytoplankton and heterotrophic bacteria in the northwest subarctic Pacific to in situ iron fertilization as estimated by HPLC pigment analysis and flow cytometry. Prog. Oceanogr..

[82] Nagai S (2016). Influences of diurnal sampling bias on fixed-point monitoring of plankton biodiversity determined using a massively parallel sequencing-based technique. Gene.

[83] Tanabe AS (2016). Comparative study of the validity of three regions of the 18S-rRNA gene for massively parallel sequencing-based monitoring of the planktonic eukaryote community. Mol. Ecol. Resour..

[84] Dzhembekova N, Moncheva S, Ivanova P, Slabakova N, Nagai S (2018). Biodiversity of phytoplankton cyst assemblages in surface sediments of the Black Sea based on metabarcoding. Biotechnol. Biotechnol. Equip..

[85] Dzhembekova N, Urusizaki S, Moncheva S, Ivanova P, Nagai S (2017). Applicability of massively parallel sequencing on monitoring harmful algae at Varna Bay in the Black Sea. Harmful Algae.

[86] Bolger AM, Lohse M, Usadel B (2014). Trimmomatic: a flexible trimmer for Illumina sequence data. Bioinformatics.

[87] Schloss PD, Gevers D, Westcott SL (2011). Reducing the effects of PCR amplification and sequencing artifacts on 16S rRNA-based studies. PLoS ONE.

[88] Edgar RC, Haas BJ, Clemente JC, Quince C, Knight R (2011). UCHIME improves sensitivity and speed of chimera detection. Bioinformatics.

[89] Cheung KLY, Huen J, Houry WA, Ortega J (2010). Comparison of the multiple oligomeric structures observed for the Rvb1 and Rvb2 proteins. Biochem. Cell Biol..

[90] Camacho C (2009). BLAST+: architecture and applications. BMC Bioinform..

[91] Horton, T. *et al.* World register of marine species (WoRMS) (2018).

[92] R Core team. R: A language and environment for statistical computing. R Foundation for Statistical Computing, Vienna, Austria. URL https://www.R-project.org/. *R: A Language and Environment for Statistical Computing. R Foundation for Statistical Computing , Vienna, Austria.* ISBN 3-900051-07-0, URL http://www.R-project.org/ (2017). 10.2788/95827.

[93] Bray JR, Curtis JT (1957). An ordination of the upland forest communities of southern Wisconsin. Ecol. Monogr..

[94] Anderson MJ (2001). A new method for non-parametric multivariate analysis of variance. Aust. Ecol..

[95] Oksanen, J. *et al.* vegan: Community ecology package. R package version 2.5-2. *CRAN R* (2018). ISBN 0-387-95457-0.

