# Supplementary material for: 18S rRNA gene sequences of leptocephalus gut contents, particulate organic matter, and biological oceanographic conditions in the western North Pacific
Source: Sci Rep. 2021 Mar 9;11:5488. doi: 10.1038/s41598-021-84532-y (PMC7930194; doi:10.1038/s41598-021-84532-y)
Supplement: Supplementary file 1 — Supplementary Information [file 41598_2021_84532_MOESM1_ESM.pdf]

Electronic supplementary material

18S rRNA gene sequences of leptocephalus gut contents, particulate organic matter, and biological oceanographic conditions in the western North Pacific

Tsuyoshi Watanabe*, Satoshi Nagai, Yoko Kawakami, Taiga Asakura, Jun Kikuchi, Nobuharu Inaba, Yukiko Taniuchi, Hiroaki Kurogi, Seinen Chow, Tsutomu Tomoda, Daisuke Ambe, Daisuke Hasegawa

*Corresponding author: tsuyoshiw@affrc.go.jp
Current affiliation: Fisheries Technology Institute, Japan Fisheries Research and Education Agency, 116 Katsurakoi, Kushiro, Hokkaido 085-0802, Japan

Supplementary Figure and Table

Supplementary Figure 1. Eukaryotic composition of larval eel gut contents and POM of seawater. The relative abundance of eukaryotic composition and number of taxa in 86 POM samples and 75 eel larva gut contents.

Supplementary Table 1. Environmental and phytoplankton data at standard discrete depths in this study

Supplementary Table 2. Sample information used for metagenomic analysis of larval eel gut contents and POM in this study

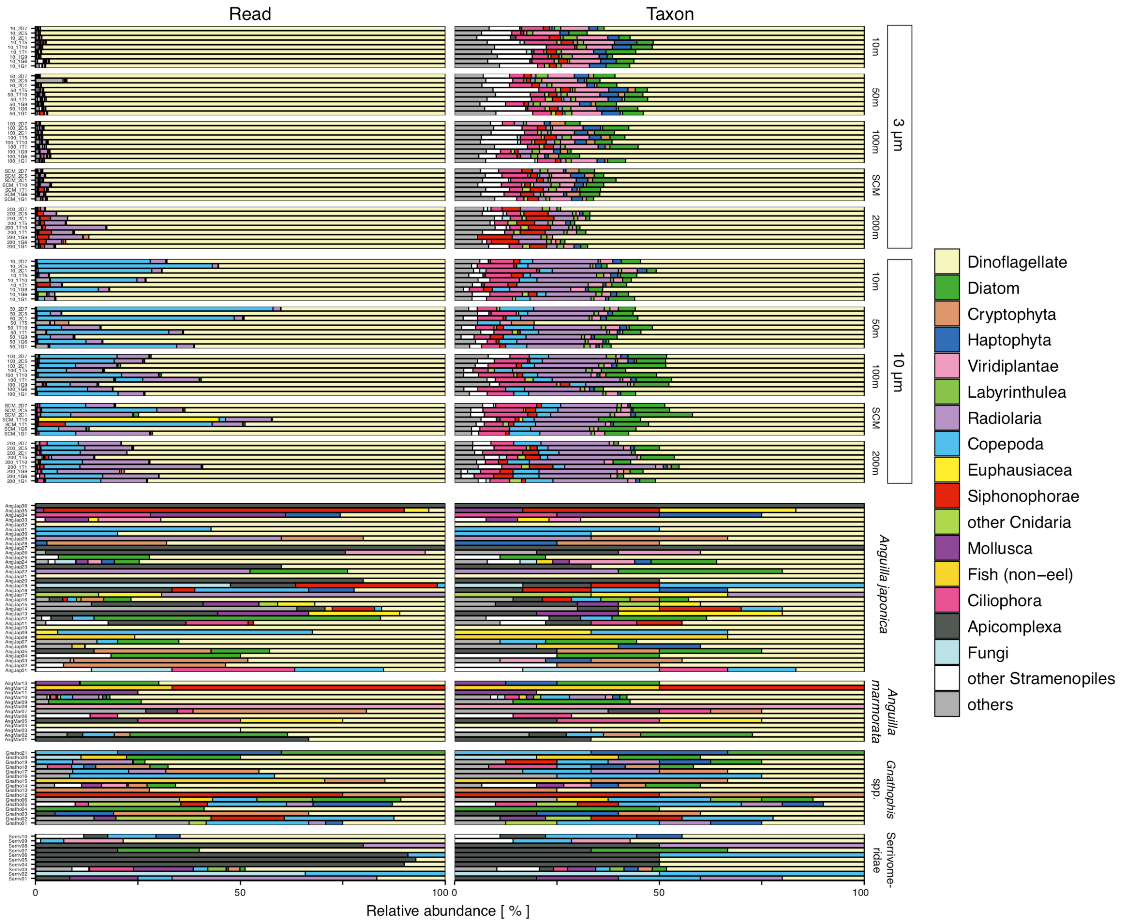

Supplementary Figure 1. Eukaryotic composition of larval eel gut contents and POM of seawater. The relative abundance of eukaryotic composition and number of taxa in 86 POM samples and 75 eel larva gut contents.

Supplementary Table 1. Environmental and phytoplankton data at standard discrete depths in this study

No	Cruise	Leg	Station	Date	Time	Latitude [°N]	Longitude [°E]	Layer	Depth [m]	Temperature [°C]	Salinity [psu]	Sigma-t [kg/m^3]	DO [mL/L]	FISP [μg/L]	NO3 [μM]	NO2 [μM]	Si [μM]	PO4 [μM]	Chl-a [μg/L]	Chl-a > 10 μm [μg/L]	Chl-a 3-10 μm [μg/L]	Chl-a 0.2-3 μm [μg/L]	Picoeukaryotes [cells/mL]	Synechococcus [cells/mL]	Dinoflagellate [cells/L]	Other phytoplankton [cells/L]
1	KY1604	Leg1	1T1	28-Sep-16	15:58	14.00	138.00	0	0	29.4					0.17	0.07	1.14	0.04	0.05							
2	KY1604	Leg1	1T1	28-Sep-16	15:58	14.00	138.00	10	10	29.4	34.3	21.4	6.0	0.04	0.04	0.05	1.20	0.02	0.05						6713	594
3	KY1604	Leg1	1T1	28-Sep-16	15:58	14.00	138.00	50	50	29.4	34.4	21.5	6.0	0.05	0.00	0.09	1.15	0.04	0.07						3200	220
4	KY1604	Leg1	1T1	28-Sep-16	15:58	14.00	138.00	75	75	28.1	34.9	22.3	6.4	0.07												
5	KY1604	Leg1	1T1	28-Sep-16	15:58	14.00	138.00	100	100	26.7	35.0	22.8	6.2	0.13	0.01	0.10	1.26	0.05	0.18						3162	488
6	KY1604	Leg1	1T1	28-Sep-16	15:58	14.00	138.00	150	150	24.1	35.2	23.8	5.8	0.12												
7	KY1604	Leg1	1T1	28-Sep-16	15:58	14.00	138.00	200	200	18.5	34.9	25.1	5.4	0.03	5.18	0.00	4.33	0.33	0.02						3071	622
8	KY1604	Leg1	1T1	28-Sep-16	15:58	14.00	138.00	300	300	12.1	34.4	26.1	3.7	0.02	11.48	-0.01	12.86	0.67								
9	KY1604	Leg1	1T1	28-Sep-16	15:58	14.00	138.00	500	500	7.1	34.4	26.9	2.2	0.03												
10	KY1604	Leg1	1T1	28-Sep-16	15:58	14.00	138.00	750	750	5.3	34.5	27.2	2.3	0.03												
11	KY1604	Leg1	1T1	28-Sep-16	15:58	14.00	138.00	1000	1000	4.3	34.5	27.4	2.5	0.03												
12	KY1604	Leg1	1T1	28-Sep-16	15:58	14.00	138.00	SCM	113	25.8	35.2	23.2	6.1	0.17	0.92	0.01	1.28	0.06	0.18	0.01	0.01	0.14			6328	551
13	KY1604	Leg1	1T2	28-Sep-16	23:45	14.02	137.01	0	0	28.7	34.6				0.11	-0.01	1.18	0.03	0.04							
14	KY1604	Leg1	1T2	28-Sep-16	23:45	14.02	137.01	10	10	29.4	34.6	21.6	6.0	0.04	0.04	-0.01	1.20	0.01	0.05							
15	KY1604	Leg1	1T2	28-Sep-16	23:45	14.02	137.01	50	50	28.5	35.0	22.2	6.4	0.05	-0.01	-0.02	1.15	0.01	0.08							
16	KY1604	Leg1	1T2	28-Sep-16	23:45	14.02	137.01	75	75	25.9	35.0	23.0	6.0	0.14												
17	KY1604	Leg1	1T2	28-Sep-16	23:45	14.02	137.01	100	100	23.9	35.1	23.8	5.7	0.23	0.58	0.14	1.80	0.10	0.22							
18	KY1604	Leg1	1T2	28-Sep-16	23:45	14.02	137.01	150	150	21.3	35.1	24.5	5.6	0.10												
19	KY1604	Leg1	1T2	28-Sep-16	23:45	14.02	137.01	200	200	16.8	34.7	25.3	5.5	0.02	8.15	0.00	7.15	0.52	0.00							
20	KY1604	Leg1	1T2	28-Sep-16	23:45	14.02	137.01	300	300	11.7	34.4	26.2	3.9	0.02	21.92	-0.01	24.63	1.53								
21	KY1604	Leg1	1T2	28-Sep-16	23:45	14.02	137.01	500	500	7.4	34.4	26.9	2.3	0.03												
22	KY1604	Leg1	1T2	28-Sep-16	23:45	14.02	137.01	750	750	5.2	34.5	27.2	2.4	0.03												
23	KY1604	Leg1	1T2	28-Sep-16	23:45	14.02	137.01	1000	1000	4.3	34.5	27.4	2.6	0.03												
24	KY1604	Leg1	1T2	28-Sep-16	23:45	14.02	137.01	SCM	90	25.3	35.0	23.3	5.9	0.28	0.19	0.04	1.83	0.13	0.26	0.01	0.03	0.26				
25	KY1604	Leg1	1T3	29-Sep-16	15:59	14.00	136.00	0	0	29.4	34.6				0.61	0.01	1.05	0.00	0.05							
26	KY1604	Leg1	1T3	29-Sep-16	15:59	14.00	136.00	10	10	29.5	34.5	21.5	6.0	0.04	0.15	0.00	1.26	0.04	0.05							
27	KY1604	Leg1	1T3	29-Sep-16	15:59	14.00	136.00	50	50	29.3	34.6	21.7	6.1	0.05	0.00	0.00	1.30	0.01	0.07							
28	KY1604	Leg1	1T3	29-Sep-16	15:59	14.00	136.00	75	75	27.4	34.9	22.6	6.3	0.08												
29	KY1604	Leg1	1T3	29-Sep-16	15:59	14.00	136.00	100	100	25.4	35.0	23.2	5.9	0.35	0.36	0.15	1.82	0.13	0.24							
30	KY1604	Leg1	1T3	29-Sep-16	15:59	14.00	136.00	150	150	20.0	35.0	24.8	5.6	0.07												
31	KY1604	Leg1	1T3	29-Sep-16	15:59	14.00	136.00	200	200	16.3	34.7	25.4	5.5	0.03	8.45	0.00	7.40	0.56	0.01							
32	KY1604	Leg1	1T3	29-Sep-16	15:59	14.00	136.00	300	300	11.1	34.4	26.3	3.4	0.02	24.38	-0.01	25.89	1.58								
33	KY1604	Leg1	1T3	29-Sep-16	15:59	14.00	136.00	500	500	6.8	34.4	27.0	2.5	0.03												
34	KY1604	Leg1	1T3	29-Sep-16	15:59	14.00	136.00	750	750	5.5	34.5	27.2	2.7	0.03												
35	KY1604	Leg1	1T3	29-Sep-16	15:59	14.00	136.00	1000	1000	4.3	34.5	27.4	2.7	0.03												
36	KY1604	Leg1	1T3	29-Sep-16	15:59	14.00	136.00	SCM	100	25.4	35.0	23.2	5.9	0.35	0.36	0.15	1.82	0.13	0.24	0.02	0.02	0.20				
37	KY1604	Leg1	1T4	30-Sep-16	0:55	14.02	135.00	0	0	28.0	34.6				0.15	0.00	1.13	0.03	0.05							
38	KY1604	Leg1	1T4	30-Sep-16	0:55	14.02	135.00	10	10	29.7	34.6	21.5	6.0	0.04	-0.04	0.00	1.12	0.01	0.05							
39	KY1604	Leg1	1T4	30-Sep-16	0:55	14.02	135.00	50	50	29.5	34.6	21.6	5.9	0.04	-0.03	-0.04	0.95	0.01	0.05							
40	KY1604	Leg1	1T4	30-Sep-16	0:55	14.02	135.00	75	75	28.6	34.8	22.0	6.2	0.06												
41	KY1604	Leg1	1T4	30-Sep-16	0:55	14.02	135.00	100	100	27.0	35.0	22.7	6.2	0.12	0.01	-0.04	1.26	0.06	0.16							
42	KY1604	Leg1	1T4	30-Sep-16	0:55	14.02	135.00	150	150	22.8	35.2	24.1	5.6	0.14												
43	KY1604	Leg1	1T4	30-Sep-16	0:55	14.02	135.00	200	200	18.8	34.9	25.0	5.5	0.06	5.80	-0.02	4.72	0.38	0.03							
44	KY1604	Leg1	1T4	30-Sep-16	0:55	14.02	135.00	300	300	12.2	34.4	26.1	4.1	0.02	21.00	-0.03	22.08	1.39								
45	KY1604	Leg1	1T4	30-Sep-16	0:55	14.02	135.00	500	500	6.8	34.3	26.9	2.4	0.03												
46	KY1604	Leg1	1T4	30-Sep-16	0:55	14.02	135.00	750	750	5.3	34.5	27.3	2.8	0.03												
47	KY1604	Leg1	1T4	30-Sep-16	0:55	14.02	135.00	1000	1000	4.2	34.5	27.4	2.6	0.03												
48	KY1604	Leg1	1T4	30-Sep-16	0:55	14.02	135.00	SCM	116	25.6	35.0	23.2	5.9	0.22	0.04	-0.03	1.54	0.09	0.20	0.01	0.01	0.18				
49	KY1604	Leg1	1T5	30-Sep-16	15:55	14.00	134.00	0	0	29.3					0.32	0.02	1.39	0.01	0.06							
50	KY1604	Leg1	1T5	30-Sep-16	15:55	14.00	134.00	10	10	29.6	34.6	21.5	6.0	0.04	0.06	-0.03	1.26	0.01	0.06						17060	644
51	KY1604	Leg1	1T5	30-Sep-16	15:55	14.00	134.00	50	50	29.6	34.6	21.5	5.9	0.06	-0.02	-0.03	1.17	0.01	0.07						10181	684
52	KY1604	Leg1	1T5	30-Sep-16	15:55	14.00	134.00	75	75	27.5	34.9	22.5	6.3	0.09												
53	KY1604	Leg1	1T5	30-Sep-16	15:55	14.00	134.00	100	100	26.3	35.1	23.0	6.2	0.12	-0.11	-0.03	1.02	0.00	0.17						11361	6668
54	KY1604	Leg1	1T5	30-Sep-16	15:55	14.00	134.00	150	150	22.0	35.1	24.3	5.5	0.11												
55	KY1604	Leg1	1T5	30-Sep-16	15:55	14.00	134.00	200	200	18.1	34.8	25.1	4.9	0.04	7.39	-0.03	6.26	0.49	0.03						2514	85
56	KY1604	Leg1	1T5	30-Sep-16	15:55	14.00	134.00	300	300	12.6	34.4	26.0	4.2	0.02	19.25	-0.03	19.70	1.13								
57	KY1604	Leg1	1T5	30-Sep-16	15:55	14.00	134.00	500	500	7.3	34.3	26.8	2.3	0.03												
58	KY1604	Leg1	1T5	30-Sep-16	15:55	14.00	134.00	750	750	5.1	34.5	27.2	2.4	0.03												
59	KY1604	Leg1	1T5	30-Sep-16	15:55	14.00	134.00	1000	1000	4.1	34.5	27.4	2.6	0.03												
60	KY1604	Leg1	1T5	30-Sep-16	15:55	14.00	134.00	SCM	120	24.6	35.2	23.6	5.8	0.23	0.01	-0.03	1.20	0.03	0.23	0.01	0.01	0.21			4650	522
61	KY1604	Leg1	1T6	1-Oct-16	0:13	14.01	133.02	0	0	29.4	34.6				0.00	-0.03	1.31	0.02	0.06							
62	KY1604	Leg1	1T6	1-Oct-16	0:13	14.01	133.02	10	10	29.7	34.6	21.5	6.0	0.04	0.05	-0.03	1.25	0.02	0.06							
63	KY1604	Leg1	1T6	1-Oct-16	0:13	14.01	133.02	50	50	29.7	34.6	21.5	6.0	0.05	-0.01	-0.03	1.22	0.03	0.07							
64	KY1604	Leg1	1T6	1-Oct-16	0:13	14.01	133.02	75	75	28.0	34.9	22.3	6.3	0.08												
65	KY1604	Leg1	1T6	1-Oct-16	0:13	14.01	133.02	100	100	27.0	35.0	22.7	6.2	0.15	-0.04	-0.02	1.22	0.04	0.21							
66	KY1604	Leg1	1T6	1-Oct-16	0:13	14.01	133.02	150	150	22.5	35.1	24.2	5.4	0.09												
67	KY1604	Leg1	1T6	1-Oct-16	0:13	14.01	133.02	200	200	18.8	34.9	25.0	5.3	0.03	5.07	-0.01	4.43	0.35	0.02							
68	KY1604	Leg1	1T6	1-Oct-16	0:13	14.01	133.02	300	300	12.9	34.4	26.0	4.3	0.02	20.06	-0.03	20.11	1.34								
69	KY1604	Leg1	1T6	1-Oct-16	0:13	14.01	133.02	500	500	7.2	34.3	26.9	2.3	0.03												
70	KY1604	Leg1	1T6	1-Oct-16	0:13	14.01	133.02	750	750	5.2	34.5	27.2	2.3	0.03												
71	KY1604	Leg1	1T6	1-Oct-16	0:13	14.01	133.02	1000	1000	4.1	34.5	27.4	2.7	0.03												
72	KY1604	Leg1	1T6	1-Oct-16	0:13	14.01	133.02	SCM	120	25.9	35.1	23.1	6.0	0.21	0.04	0.02	1.46	0.08	0.21	0.01	0.02	0.20				
73	KY1604	Leg1	1T7	1-Oct-16	16:55	14.00	132.00	0	0	29.4	34.5				0.07	-0.02	1.27	0.01	0.05							
74	KY1604	Leg1	1T7	1-Oct-16	16:55	14.00	132.00	10	10	29.7	34.5	21.4	6.0	0.05	0.00	-0.02	1.18	0.02	0.04							
75	KY1604	Leg1	1T7	1-Oct-16	16:55	14.00	132.00	50	50	29.7	34.5	21.4	5.9	0.05	0.01	-0.02	1.13	0.00	0.07							
76	KY1604	Leg1	1T7	1-Oct-16	16:55	14.00	132.00	75	75	28.0	34.9	22.3	6.3	0.08												
77	KY1604	Leg1	1T7	1-Oct-16	16:55	14.00	132.00	100	100	26.6	35.1	22.9	6.2	0.15	0.01	-0.02	1.20	0.02	0.17							
78	KY1604	Leg1	1T7	1-Oct-16	16:55	14.00	132.00	150	150	21.1	35.1	24.5	5.4	0.08												
79	KY1604	Leg1	1T7	1-Oct-16	16:55	14.00	132.00	200	200	17.7	34.8	25.2	5.4	0.02	6.97	-0.02	5.68	0.46	0.00							
80	KY1604	Leg1	1T7	1-Oct-16	16:55	14.00	132.00	300	300	12.1	34.4	26.1	4.5	0.02	19.17	-0.02	20.94	1.24								
81	KY1604	Leg1	1T7	1-Oct-16	16:55	14.00	132.00	500	500	7.3	34.3	26.9	2.2	0.03												
82	KY1604	Leg1	1T7	1-Oct-16	16:55	14.00	132.00	750	750	5.2	34.5	27.3	2.4	0.03												
83	KY1604	Leg1	1T7	1-Oct-16	16:55	14.00	132.00	1000	1000	4.1	34.5	27.4	2.7	0.03												
84	KY1604	Leg1	1T7	1-Oct-16	16:55	14.00	132.00	SCM	110	25.3	35.1	23.3	5.9	0.24	0.09	0.02	1.53	0.08	0.21	0.01	0.01	0.21				
85	KY1604	Leg1	1T10	2-Oct-16	16:56	14.00	129.00	0	0	29.9					0.01	-0.01	1.10	-0.01	0.06							
86	KY1604	Leg1	1T10	2-Oct-16	16:56	14.00	129.00	10	10	30.0	34.5	21.4	5.9	0.05	-0.04	-0.02	1.20	0.00	0.06						13691	998
87	KY1604	Leg1	1T10	2-Oct-16	16:56	14.00	129.00	50	50	29.8	34.7	21.6	5.9	0.06	-0.03	-0.01	1.30	-0.01	0.08						14604	656
88	KY1604	Leg1	1T10	2-Oct-16	16:56	14.00	129.00	75	75	27.6	34.9	22.4	6.3	0.07												
89	KY1604	Leg1	1T10	2-Oct-16	16:56	14.00	129.00	100	100	26.2	35.1	23.0	6.1	0.21	0.17	0.14	1.29	0.03	0.20						6118	1612
90	KY1604	Leg1	1T10	2-Oct-16	16:56	14.00	129.00	150	150	22.6	35.2	24.2	5.4	0.11												
91	KY1604	Leg1	1T10	2-Oct-16	16:56	14.00	129.00	200	200	19.0	34.9	24.9	5.2	0.04	8.55	-0.01	7.00	0.59	0.01						3133	285
92	KY1604	Leg1	1T10	2-Oct-16	16:56	14.00	129.00	300	300	13.0	34.4	25.9	5.0	0.02	19.44	-0.01	21.17	1.35								
93	KY1604	Leg1	1T10	2-Oct-16	16:56	14.00	129.00	500	500	7.0	34.2	26.8	2.6	0.03												
94	KY1604	Leg1	1T10	2-Oct-16	16:56	14.00	129.00	750	750	4.6	34.5	27.3	2.3	0.03												
95	KY1604	Leg1	1T10	2-Oct-16	16:56	14.00	129.00	1000	1000	3.9	34.6	27.4	2.8	0.03												
96	KY1604	Leg1	1T10	2-Oct-16	16:56	14.00	129.00	SCM	112	25.2	35.1	23.4	6.0	0.22	0.28	0.15	1.39	0.05	0.19	0.01	0.02	0.11			7951	1986
97	KY1604	Leg1	1T8	2-Oct-16	1:29	13.98	131.02	0	0	29.6	34.4				0.01	-0.01	1.36	0.02	0.05							
98	KY1604	Leg1	1T8	2-Oct-16	1:29	13.98	131.02	10	10	29.8	34.4	21.4	5.9	0.04	0.07	-0.01	1.40	0.00	0.05							
99	KY1604	Leg1	1T8	2-Oct-16	1:29	13.98	131.02	50	50	29.8	34.4	21.4	6.0	0.05	0.23	0.00	1.21	0.02	0.05							
100	KY1604	Leg1	1T8	2-Oct-16	1:29	13.98	131.02	75	75	28.3	34.8	22.2	6.4	0.07												
101	KY1604	Leg1	1T8	2-Oct-16	1:29	13.98	131.02	100	100	26.6	35.1	22.9	6.1	0.15	-0.02	-0.01	1.13	0.03	0.20							
102	KY1604	Leg1	1T8	2-Oct-16	1:29	13.98	131.02	150	150	21.9	35.1	24.4	5.5	0.08												
103	KY1604	Leg1	1T8	2-Oct-16	1:29	13.98	131.02	200	200	17.2	34.7	25.2	4.4	0.03	11.00	-0.01	9.46	0.73	0.01							
104	KY1604	Leg1	1T8	2-Oct-16	1:29	13.98	131.02	300	300	11.5	34.3	26.2	4.7	0.02	20.52	-0.01	22.62	1.34								
105	KY1604	Leg1	1T8	2-Oct-16	1:29	13.98	131.02	500	500	6.9	34.3	26.9	2.4	0.03												
106	KY1604	Leg1	1T8	2-Oct-16	1:29	13.98	131.02	750	750	5.1	34.5	27.3	2.4	0.03												
107	KY1604	Leg1	1T8	2-Oct-16	1:29	13.98	131.02	1000	1000	4.0	34.5	27.4	2.7	0.03												
108	KY1604	Leg1	1T8	2-Oct-16	1:29	13.98	131.02	SCM	112	25.6	35.2	23.3	5.9	0.19	0.10	0.03	1.33	0.07	0.19	0.01	0.01	0.17				
109	KY1604	Leg1	1T9	3-Oct-16	1:06	14.00	129.98	0	0	28.5	34.5				0.06	-0.01	1.38	0.01	0.05							
110	KY1604	Leg1	1T9	3-Oct-16	1:06	14.00	129.98	10	10	29.9	34.5	21.4	5.9	0.05	0.01	-0.02	1.44	0.00	0.05							
111	KY1604	Leg1	1T9	3-Oct-16	1:06	14.00	129.98	50	50	29.9	34.5	21.4	6.0	0.05	-0.01	-0.02	1.28	0.02	0.07							
112	KY1604	Leg1	1T9	3-Oct-16	1:06	14.00	129.98	75	75	28.2	34.8	22.2	6.3	0.08												
113	KY1604	Leg1	1T9	3-Oct-16	1:06	14.00	129.98	100	100	27.1	35.0	22.7	6.2	0.11	0.01	-0.01	1.37	0.05	0.16							
114	KY1604	Leg1	1T9	3-Oct-16	1:06	14.00	129.98	150	150	24.1	35.2	23.7	5.6	0.18												
115	KY1604	Leg1	1T9	3-Oct-16	1:06	14.00	129.98	200	200	19.8	35.0	24.8	5.4	0.03	4.54	0.00	3.84	0.30	0.02							
116	KY1604	Leg1	1T9	3-Oct-16	1:06	14.00	129.98	300	300	11.9	34.3	26.1	4.9	0.02	17.49	-0.02	19.16	1.14								
117	KY1604	Leg1	1T9	3-Oct-16	1:06	14.00	129.98	500	500	6.6	34.4	27.0	2.3	0.03												
118	KY1604	Leg1	1T9	3-Oct-16	1:06	14.00	129.98	750	750	4.9	34.5	27.3	2.4	0.03												
119	KY1604	Leg1	1T9	3-Oct-16	1:06	14.00	129.98	1000	1000	3.9	34.5	27.4	2.6	0.03												
120	KY1604	Leg1	1T9	3-Oct-16	1:06	14.00	129.98	SCM	135	25.2	35.2	23.4	5.8	0.21	0.12	0.04	1.32	0.04	0.19	0.01	0.01	0.16				

Supplementary Table 1. Continued 1

No	Cruise	Leg	Station	Date	Time	Latitude [°N]	Longitude [°E]	Layer	Depth [m]	Temperature [°C]	Salinity [psu]	Sigma-t [kg/m^3]	DO [mL/L]	FISP [μg/L]	NO3 [μM]	NO2 [μM]	Si [μM]	PO4 [μM]	Chl-a [μg/L]	Chl-a >10 μm [μg/L]	Chl-a 3-10 μm [μg/L]	Chl-a 0.2-3 μm [μg/L]	Picoeukaryotes [cells/mL]	Synechococcus [cells/mL]	Dinoflagellate [cells/L]	Other phytoplankton [cells/L]
121	KY1604	Leg1	1G1	5-Oct-16	16:59	22.50	133.25	0	0	28.4					-0.03	-0.02	1.11	-0.01	0.05							
122	KY1604	Leg1	1G1	5-Oct-16	16:59	22.50	133.25	10	10	29.3	34.7	21.8	6.0	0.04	-0.06	-0.01	1.15	-0.01	0.05						8375	488
123	KY1604	Leg1	1G1	5-Oct-16	16:59	22.50	133.25	50	50	29.2	34.7	21.8	6.0	0.05	-0.02	-0.02	1.00	-0.01	0.06						9450	663
124	KY1604	Leg1	1G1	5-Oct-16	16:59	22.50	133.25	75	75	29.0	34.9	22.0	6.1	0.08												
125	KY1604	Leg1	1G1	5-Oct-16	16:59	22.50	133.25	100	100	27.1	35.0	22.7	6.3	0.11	-0.06	-0.01	1.08	-0.01	0.12						5497	870
126	KY1604	Leg1	1G1	5-Oct-16	16:59	22.50	133.25	150	150	24.8	35.2	23.6	5.9	0.17												
127	KY1604	Leg1	1G1	5-Oct-16	16:59	22.50	133.25	200	200	21.1	35.1	24.5	5.7	0.04	1.65	0.00	2.04	0.11	0.02						3770	291
128	KY1604	Leg1	1G1	5-Oct-16	16:59	22.50	133.25	300	300	17.4	34.8	25.2	6.1	0.02	4.97	-0.01	4.01	0.27								
129	KY1604	Leg1	1G1	5-Oct-16	16:59	22.50	133.25	500	500	11.9	34.3	26.1	5.4	0.02												
130	KY1604	Leg1	1G1	5-Oct-16	16:59	22.50	133.25	750	750	5.8	34.2	26.9	2.5	0.03												
131	KY1604	Leg1	1G1	5-Oct-16	16:59	22.50	133.25	1000	1000	4.2	34.4	27.3	1.7	0.03												
132	KY1604	Leg1	1G1	5-Oct-16	16:59	22.50	133.25	SCM	130	26.0	35.2	23.2	6.2	0.21	0.38	-0.01	1.06	-0.01	0.21	0.01	0.01	0.19			5433	1027
133	KY1604	Leg1	1G2	5-Oct-16	21:55	22.20	133.10	0	0	29.1	34.7				-0.03	-0.02	1.15	-0.01	0.05							
134	KY1604	Leg1	1G2	5-Oct-16	21:55	22.20	133.10	10	10	28.9	34.7	21.8	6.0	0.04	-0.04	-0.01	1.03	-0.01	0.04							
135	KY1604	Leg1	1G2	5-Oct-16	21:55	22.20	133.10	50	50	28.9	34.7	21.8	6.0	0.05	-0.06	-0.01	1.15	-0.02	0.05							
136	KY1604	Leg1	1G2	5-Oct-16	21:55	22.20	133.10	75	75	27.9	35.0	22.5	6.4	0.09												
137	KY1604	Leg1	1G2	5-Oct-16	21:55	22.20	133.10	100	100	26.2	35.1	23.1	6.2	0.17	-0.04	-0.01	1.17	0.01	0.22							
138	KY1604	Leg1	1G2	5-Oct-16	21:55	22.20	133.10	150	150	23.5	35.2	23.9	5.8	0.08												
139	KY1604	Leg1	1G2	5-Oct-16	21:55	22.20	133.10	200	200	20.6	35.0	24.6	5.7	0.03	2.14	0.01	2.15	0.14	0.01							
140	KY1604	Leg1	1G2	5-Oct-16	21:55	22.20	133.10	300	300	16.9	34.7	25.3	6.0	0.02	6.11	-0.01	4.94	0.34								
141	KY1604	Leg1	1G2	5-Oct-16	21:55	22.20	133.10	500	500	10.8	34.3	26.2	5.2	0.02												
142	KY1604	Leg1	1G2	5-Oct-16	21:55	22.20	133.10	750	750	5.5	34.2	27.0	2.2	0.03												
143	KY1604	Leg1	1G2	5-Oct-16	21:55	22.20	133.10	1000	1000	4.0	34.4	27.3	1.8	0.03												
144	KY1604	Leg1	1G2	5-Oct-16	21:55	22.20	133.10	SCM	100	26.2	35.1	23.1	6.2	0.17	-0.04	0.00	1.10	0.00	0.22	0.01	0.01	0.19				
145	KY1604	Leg1	1G3	6-Oct-16	2:36	21.88	133.00	0	0	28.9	34.7				-0.04	-0.01	1.18	-0.01	0.05							
146	KY1604	Leg1	1G3	6-Oct-16	2:36	21.88	133.00	10	10	28.9	34.7	21.9	6.0	0.04	0.67	0.00	0.97	-0.01	0.04							
147	KY1604	Leg1	1G3	6-Oct-16	2:36	21.88	133.00	50	50	28.9	34.7	21.9	6.0	0.04	0.15	-0.01	1.13	-0.01	0.05							
148	KY1604	Leg1	1G3	6-Oct-16	2:36	21.88	133.00	75	75	28.4	34.7	22.0	6.2	0.07												
149	KY1604	Leg1	1G3	6-Oct-16	2:36	21.88	133.00	100	100	26.1	34.9	22.9	6.5	0.10	-0.05	0.00	1.39	0.00	0.20							
150	KY1604	Leg1	1G3	6-Oct-16	2:36	21.88	133.00	150	150	22.0	35.0	24.2	6.3	0.12												
151	KY1604	Leg1	1G3	6-Oct-16	2:36	21.88	133.00	200	200	19.8	35.0	24.8	5.6	0.03	3.57	0.01	3.07	0.23	0.01							
152	KY1604	Leg1	1G3	6-Oct-16	2:36	21.88	133.00	300	300	16.6	34.7	25.4	6.1	0.02	7.58	0.00	6.41	0.44								
153	KY1604	Leg1	1G3	6-Oct-16	2:36	21.88	133.00	500	500	10.7	34.3	26.2	4.7	0.02												
154	KY1604	Leg1	1G3	6-Oct-16	2:36	21.88	133.00	750	750	5.7	34.2	27.0	2.3	0.03												
155	KY1604	Leg1	1G3	6-Oct-16	2:36	21.88	133.00	1000	1000	4.0	34.4	27.3	1.8	0.03												
156	KY1604	Leg1	1G3	6-Oct-16	2:36	21.88	133.00	SCM	132	22.8	35.0	24.0	6.5	0.15	1.01	0.00	1.44	0.00	0.22	0.01	0.02					
157	KY1604	Leg1	1G8	6-Oct-16	21:17	20.16	132.41	0	0	28.5	34.7				-0.02	0.00	1.08	-0.05	0.03							
158	KY1604	Leg1	1G8	6-Oct-16	21:17	20.16	132.41	10	10	28.2	34.6	22.1	6.1	0.03	0.02	0.00	0.95	-0.04	0.03							
159	KY1604	Leg1	1G8	6-Oct-16	21:17	20.16	132.41	50	50	27.0	34.8	22.6	6.3	0.06	0.01	-0.01	1.25	-0.03	0.07							
160	KY1604	Leg1	1G8	6-Oct-16	21:17	20.16	132.41	75	75	24.0	34.9	23.6	6.4	0.10												
161	KY1604	Leg1	1G8	6-Oct-16	21:17	20.16	132.41	100	100	22.3	35.0	24.1	6.1	0.26	0.33	0.15	1.61	0.02	0.26							
162	KY1604	Leg1	1G8	6-Oct-16	21:17	20.16	132.41	150	150	20.2	34.9	24.7	5.9	0.10												
163	KY1604	Leg1	1G8	6-Oct-16	21:17	20.16	132.41	200	200	18.5	34.8	25.0	6.0	0.02	4.93	0.00	4.06	0.30	0.00							
164	KY1604	Leg1	1G8	6-Oct-16	21:17	20.16	132.41	300	300	16.1	34.6	25.5	6.0	0.02	8.84	-0.01	8.62	0.58								
165	KY1604	Leg1	1G8	6-Oct-16	21:17	20.16	132.41	500	500	9.9	34.2	26.4	4.8	0.02												
166	KY1604	Leg1	1G8	6-Oct-16	21:17	20.16	132.41	750	750	5.1	34.3	27.1	1.9	0.03												
167	KY1604	Leg1	1G8	6-Oct-16	21:17	20.16	132.41	1000	1000	3.7	34.5	27.4	2.0	0.03												
168	KY1604	Leg1	1G8	6-Oct-16	21:17	20.16	132.41	SCM	100	22.3	35.0	24.1	6.1	0.26	0.29	0.14	1.78	0.01	0.26	0.01	0.03	0.25				
169	KY1604	Leg1	1G9	6-Oct-16	17:00	19.87	132.31	0	0	29.0					0.06	0.00	0.99	-0.02	0.04							
170	KY1604	Leg1	1G9	6-Oct-16	17:00	19.87	132.31	10	10	29.1	34.7	21.8	6.1	0.03	-0.05	0.00	0.91	-0.03	0.04						13931	552
171	KY1604	Leg1	1G9	6-Oct-16	17:00	19.87	132.31	50	50	25.4	35.0	23.2	6.4	0.06	-0.01	-0.01	1.33	-0.02	0.09						4495	2426
172	KY1604	Leg1	1G9	6-Oct-16	17:00	19.87	132.31	75	75	23.3	35.0	23.9	6.4	0.10												
173	KY1604	Leg1	1G9	6-Oct-16	17:00	19.87	132.31	100	100	22.2	35.0	24.2	6.2	0.19	0.09	0.03	1.54	0.00	0.28						10608	2159
174	KY1604	Leg1	1G9	6-Oct-16	17:00	19.87	132.31	150	150	20.0	34.9	24.7	5.9	0.07												
175	KY1604	Leg1	1G9	6-Oct-16	17:00	19.87	132.31	200	200	18.4	34.8	25.0	6.0	0.02	4.26	0.01	3.29	0.22	0.01						2096	564
176	KY1604	Leg1	1G9	6-Oct-16	17:00	19.87	132.31	300	300	15.8	34.6	25.5	5.9	0.02	8.82	0.00	8.93	0.48								
177	KY1604	Leg1	1G9	6-Oct-16	17:00	19.87	132.31	500	500	9.4	34.2	26.4	4.6	0.02												
178	KY1604	Leg1	1G9	6-Oct-16	17:00	19.87	132.31	750	750	5.1	34.3	27.1	1.9	0.03												
179	KY1604	Leg1	1G9	6-Oct-16	17:00	19.87	132.31	1000	1000	3.7	34.5	27.4	2.0	0.03												
180	KY1604	Leg1	1G9	6-Oct-16	17:00	19.87	132.31	SCM	108	21.9	35.0	24.2	6.1	0.31	0.16	0.05	1.88	0.02	0.30	0.01	0.02				5244	893
181	KY1604	Leg1	1G5	7-Oct-16	21:45	21.15	132.79	0	0	28.6	34.7				0.02	-0.01	1.30	-0.04	0.04							
182	KY1604	Leg1	1G5	7-Oct-16	21:45	21.15	132.79	10	10	28.5	34.7	22.0	6.1	0.04	-0.03	0.00	1.42	-0.04	0.04							
183	KY1604	Leg1	1G5	7-Oct-16	21:45	21.15	132.79	50	50	27.7	34.8	22.3	6.2	0.05	-0.07	0.01	0.76	-0.04	0.06							
184	KY1604	Leg1	1G5	7-Oct-16	21:45	21.15	132.79	75	75	25.8	34.9	23.1	6.5	0.07												
185	KY1604	Leg1	1G5	7-Oct-16	21:45	21.15	132.79	100	100	22.3	35.0	24.1	6.5	0.12	0.06	0.01	1.11	-0.02	0.20							
186	KY1604	Leg1	1G5	7-Oct-16	21:45	21.15	132.79	150	150	20.2	35.0	24.7	5.7	0.05												
187	KY1604	Leg1	1G5	7-Oct-16	21:45	21.15	132.79	200	200	18.1	34.8	25.1	5.8	0.02	3.50	0.00	2.89	0.26	0.00							
188	KY1604	Leg1	1G5	7-Oct-16	21:45	21.15	132.79	300	300	15.8	34.6	25.5	6.0	0.02	6.94	0.01	6.93	0.50								
189	KY1604	Leg1	1G5	7-Oct-16	21:45	21.15	132.79	500	500	9.7	34.2	26.4	4.9	0.02												
190	KY1604	Leg1	1G5	7-Oct-16	21:45	21.15	132.79	750	750	5.2	34.2	27.0	2.3	0.03												
191	KY1604	Leg1	1G5	7-Oct-16	21:45	21.15	132.79	1000	1000	3.9	34.4	27.3	1.9	0.03												
192	KY1604	Leg1	1G5	7-Oct-16	21:45	21.15	132.79	SCM	110	22.0	35.1	24.3	6.1	0.26	0.03	0.00	1.55	0.00	0.24	0.01	0.02	0.23				
193	KY1604	Leg1	1G6	7-Oct-16	16:59	20.87	132.67	0	0	28.4					0.03	-0.01	1.47	-0.04	0.06							
194	KY1604	Leg1	1G6	7-Oct-16	16:59	20.87	132.67	10	10	28.4	34.7	22.1	6.1	0.03	-0.05	-0.02	1.42	-0.05	0.04						4667	1042
195	KY1604	Leg1	1G6	7-Oct-16	16:59	20.87	132.67	50	50	28.0	34.7	22.2	6.2	0.04	-0.07	-0.01	1.52	-0.05	0.05						7389	5569
196	KY1604	Leg1	1G6	7-Oct-16	16:59	20.87	132.67	75	75	26.8	34.8	22.7	6.5	0.06												
197	KY1604	Leg1	1G6	7-Oct-16	16:59	20.87	132.67	100	100	23.5	35.1	23.8	6.6	0.10	-0.01	-0.01	2.11	-0.04	0.20						6883	862
198	KY1604	Leg1	1G6	7-Oct-16	16:59	20.87	132.67	150	150	20.5	35.0	24.6	6.0	0.12												
199	KY1604	Leg1	1G6	7-Oct-16	16:59	20.87	132.67	200	200	18.5	34.9	25.1	5.7	0.03	4.78	-0.01	4.42	0.31	0.01						5365	1639
200	KY1604	Leg1	1G6	7-Oct-16	16:59	20.87	132.67	300	300	15.8	34.6	25.5	6.0	0.02	9.17	-0.01	9.28	0.58								
201	KY1604	Leg1	1G6	7-Oct-16	16:59	20.87	132.67	500	500	9.4	34.2	26.4	4.7	0.02												
202	KY1604	Leg1	1G6	7-Oct-16	16:59	20.87	132.67	750	750	5.0	34.3	27.1	2.0	0.03												
203	KY1604	Leg1	1G6	7-Oct-16	16:59	20.87	132.67	1000	1000	3.7	34.4	27.4	1.9	0.03												
204	KY1604	Leg1	1G6	7-Oct-16	16:59	20.87	132.67	SCM	126	21.9	35.0	24.3	6.2	0.26	0.07	0.08	1.96	-0.02	0.25	0.01	0.02	0.22			17845	2802
205	KY1604	Leg1	1G7	7-Oct-16	1:30	20.50	132.52	0	0	28.4	34.8				0.32	-0.01	1.23	-0.05	0.04							
206	KY1604	Leg1	1G7	7-Oct-16	1:30	20.50	132.52	10	10	28.2	34.8	22.1	6.2	0.04	-0.01	-0.02	0.95	-0.03	0.04							
207	KY1604	Leg1	1G7	7-Oct-16	1:30	20.50	132.52	50	50	27.5	34.8	22.4	6.4	0.04	0.01	-0.01	0.90	-0.03	0.06							
208	KY1604	Leg1	1G7	7-Oct-16	1:30	20.50	132.52	75	75	23.8	35.0	23.7	6.7	0.08												
209	KY1604	Leg1	1G7	7-Oct-16	1:30	20.50	132.52	100	100	23.1	35.2	24.0	5.9	0.24	0.46	0.01	1.49	0.04	0.28							
210	KY1604	Leg1	1G7	7-Oct-16	1:30	20.50	132.52	150	150	20.3	35.0	24.7	5.8	0.06												
211	KY1604	Leg1	1G7	7-Oct-16	1:30	20.50	132.52	200	200	18.2	34.8	25.1	6.0	0.02	4.28	-0.01	3.64	0.23	0.01							
212	KY1604	Leg1	1G7	7-Oct-16	1:30	20.50	132.52	300	300	16.3	34.7	25.4	6.0	0.02	6.67	0.00	6.44	0.40								
213	KY1604	Leg1	1G7	7-Oct-16	1:30	20.50	132.52	500	500	9.4	34.2	26.4	4.6	0.02												
214	KY1604	Leg1	1G7	7-Oct-16	1:30	20.50	132.52	750	750	4.9	34.3	27.1	1.9	0.03												
215	KY1604	Leg1	1G7	7-Oct-16	1:30	20.50	132.52	1000	1000	3.8	34.4	27.4	1.9	0.03												
216	KY1604	Leg1	1G7	7-Oct-16	1:30	20.50	132.52	SCM	96	23.2	35.1	24.0	6.0	0.26	0.33	0.02	1.59	0.03	0.26	0.01	0.02					
217	KY1604	Leg1	1AJ1	8-Oct-16	17:00	20.52	132.40	0	0	28.5																
218	KY1604	Leg1	1AJ1	8-Oct-16	17:00	20.52	132.40	10	10	28.5	34.8	22.1	6.1	0.03												
219	KY1604	Leg1	1AJ1	8-Oct-16	17:00	20.52	132.40	50	50	27.9	34.7	22.2	6.2	0.04												
220	KY1604	Leg1	1AJ1	8-Oct-16	17:00	20.52	132.40	75	75	27.5	34.8	22.4	6.3	0.05												
221	KY1604	Leg1	1AJ1	8-Oct-16	17:00	20.52	132.40	100	100	23.9	35.0	23.7	6.8	0.10												
222	KY1604	Leg1	1AJ1	8-Oct-16	17:00	20.52	132.40	150	150	20.5	35.0	24.6	6.0	0.14												
223	KY1604	Leg1	1AJ1	8-Oct-16	17:00	20.52	132.40	200	200	18.8	34.9	25.0	5.8	0.02												
224	KY1604	Leg1	1AJ1	8-Oct-16	17:00	20.52	132.40	300	300	16.0	34.6	25.5	6.1	0.02												
225	KY1604	Leg1	1AJ1	8-Oct-16	17:00	20.52	132.40	500	500	9.7	34.2	26.4	4.8	0.02												
226	KY1604	Leg1	1AJ1	8-Oct-16	17:00	20.52	132.40	750	750	5.2	34.3	27.1	2.0	0.03												
227	KY1604	Leg1	1AJ1	8-Oct-16	17:00	20.52	132.40	1000	1000	3.7	34.4	27.4	1.9	0.03												
228	KY1604	Leg1	1AJ1	8-Oct-16	17:00	20.52	132.40	SCM	126	21.6	35.0	24.3	6.3	0.27												
229	KY1604	Leg1	1AJ2	8-Oct-16	20:13	20.53	132.35	0	0																	
230	KY1604	Leg1	1AJ2	8-Oct-16	20:13	20.53	132.35	10	10																	
231	KY1604	Leg1	1AJ2	8-Oct-16	20:13	20.53	132.35	50	50																	
232	KY1604	Leg1	1AJ2	8-Oct-16	20:13	20.53	132.35	75	75																	
233	KY1604	Leg1	1AJ2	8-Oct-16	20:13	20.53	132.35	100	100																	
234	KY1604	Leg1	1AJ2	8-Oct-16	20:13	20.53	132.35	150	150																	
235	KY1604	Leg1	1AJ2	8-Oct-16	20:13	20.53	132.35	200	200																	
236	KY1604	Leg1	1AJ2	8-Oct-16	20:13	20.53	132.35	300	300																	
237	KY1604	Leg1	1AJ2	8-Oct-16	20:13	20.53	132.35	500	500																	
238	KY1604	Leg1	1AJ2	8-Oct-16	20:13	20.53	132.35	750	750																	
239	KY1604	Leg1	1AJ2	8-Oct-16	20:13	20.53	132.35	1000	1000																	
240	KY1604	Leg1	1AJ2	8-Oct-16	20:13	20.53	132.35	SCM																		

Supplementary Table 1. Continued 2

No	Cruise	Leg	Station	Date	Time	Latitude [°N]	Longitude [°E]	Layer	Depth [m]	Temperature [°C]	Salinity [psu]	Sigma-t [kg/m^3]	DO [mL/L]	FISP [μg/L]	NO3 [μM]	NO2 [μM]	Si [μM]	PO4 [μM]	Chl-a [μg/L]	Chl-a >10 μm [μg/L]	Chl-a 3-10 μm [μg/L]	Chl-a 0.2-3 μm [μg/L]	Picoeukaryotes [cells/mL]	Synechococcus [cells/mL]	Dinoflagellate [cells/L]	Other phytoplankton [cells/L]
241	KY1604	Leg1	1AJ3	8-Oct-16	21:48	20.54	132.30	0	0																	
242	KY1604	Leg1	1AJ3	8-Oct-16	21:48	20.54	132.30	10	10																	
243	KY1604	Leg1	1AJ3	8-Oct-16	21:48	20.54	132.30	50	50																	
244	KY1604	Leg1	1AJ3	8-Oct-16	21:48	20.54	132.30	75	75																	
245	KY1604	Leg1	1AJ3	8-Oct-16	21:48	20.54	132.30	100	100																	
246	KY1604	Leg1	1AJ3	8-Oct-16	21:48	20.54	132.30	150	150																	
247	KY1604	Leg1	1AJ3	8-Oct-16	21:48	20.54	132.30	200	200																	
248	KY1604	Leg1	1AJ3	8-Oct-16	21:48	20.54	132.30	300	300																	
249	KY1604	Leg1	1AJ3	8-Oct-16	21:48	20.54	132.30	500	500																	
250	KY1604	Leg1	1AJ3	8-Oct-16	21:48	20.54	132.30	750	750																	
251	KY1604	Leg1	1AJ3	8-Oct-16	21:48	20.54	132.30	1000	1000																	
252	KY1604	Leg1	1AJ3	8-Oct-16	21:48	20.54	132.30	SCM																		
253	KY1604	Leg1	1AJ4	8-Oct-16	23:00	20.56	132.26	0	0																	
254	KY1604	Leg1	1AJ4	8-Oct-16	23:00	20.56	132.26	10	10																	
255	KY1604	Leg1	1AJ4	8-Oct-16	23:00	20.56	132.26	50	50																	
256	KY1604	Leg1	1AJ4	8-Oct-16	23:00	20.56	132.26	75	75																	
257	KY1604	Leg1	1AJ4	8-Oct-16	23:00	20.56	132.26	100	100																	
258	KY1604	Leg1	1AJ4	8-Oct-16	23:00	20.56	132.26	150	150																	
259	KY1604	Leg1	1AJ4	8-Oct-16	23:00	20.56	132.26	200	200																	
260	KY1604	Leg1	1AJ4	8-Oct-16	23:00	20.56	132.26	300	300																	
261	KY1604	Leg1	1AJ4	8-Oct-16	23:00	20.56	132.26	500	500																	
262	KY1604	Leg1	1AJ4	8-Oct-16	23:00	20.56	132.26	750	750																	
263	KY1604	Leg1	1AJ4	8-Oct-16	23:00	20.56	132.26	1000	1000																	
264	KY1604	Leg1	1AJ4	8-Oct-16	23:00	20.56	132.26	SCM																		
265	KY1604	Leg1	1G4	8-Oct-16	2:19	21.47	132.90	0	0	28.9	34.7				0.24	0.00	1.61	-0.01	0.04							
266	KY1604	Leg1	1G4	8-Oct-16	2:19	21.47	132.90	10	10	28.8	34.7	21.9	6.1	0.03	0.05	-0.01	1.42	-0.04	0.04							
267	KY1604	Leg1	1G4	8-Oct-16	2:19	21.47	132.90	50	50	28.2	34.7	22.1	6.1	0.04	0.00	-0.01	1.22	-0.02	0.04							
268	KY1604	Leg1	1G4	8-Oct-16	2:19	21.47	132.90	75	75	26.1	34.9	22.9	6.5	0.07												
269	KY1604	Leg1	1G4	8-Oct-16	2:19	21.47	132.90	100	100	23.1	35.1	23.9	6.3	0.11	-0.02	0.00	1.57	-0.03	0.19							
270	KY1604	Leg1	1G4	8-Oct-16	2:19	21.47	132.90	150	150	20.8	35.0	24.5	5.8	0.14												
271	KY1604	Leg1	1G4	8-Oct-16	2:19	21.47	132.90	200	200	18.5	34.8	25.0	6.0	0.03	4.22	0.01	3.64	0.25	0.00							
272	KY1604	Leg1	1G4	8-Oct-16	2:19	21.47	132.90	300	300	16.2	34.7	25.4	6.2	0.02	6.99	0.00	6.71	0.43								
273	KY1604	Leg1	1G4	8-Oct-16	2:19	21.47	132.90	500	500	9.7	34.2	26.4	4.9	0.02												
274	KY1604	Leg1	1G4	8-Oct-16	2:19	21.47	132.90	750	750	5.2	34.2	27.0	2.2	0.03												
275	KY1604	Leg1	1G4	8-Oct-16	2:19	21.47	132.90	1000	1000	4.0	34.4	27.3	1.8	0.03												
276	KY1604	Leg1	1G4	8-Oct-16	2:19	21.47	132.90	SCM	120	21.7	35.0	24.3	6.2	0.18	0.76	0.04	1.92	0.00	0.21	0.01	0.01	0.22				
277	KY1604	Leg1	1AJ5	9-Oct-16	0:16	20.58	132.24	0	0																	
278	KY1604	Leg1	1AJ5	9-Oct-16	0:16	20.58	132.24	10	10																	
279	KY1604	Leg1	1AJ5	9-Oct-16	0:16	20.58	132.24	50	50																	
280	KY1604	Leg1	1AJ5	9-Oct-16	0:16	20.58	132.24	75	75																	
281	KY1604	Leg1	1AJ5	9-Oct-16	0:16	20.58	132.24	100	100																	
282	KY1604	Leg1	1AJ5	9-Oct-16	0:16	20.58	132.24	150	150																	
283	KY1604	Leg1	1AJ5	9-Oct-16	0:16	20.58	132.24	200	200																	
284	KY1604	Leg1	1AJ5	9-Oct-16	0:16	20.58	132.24	300	300																	
285	KY1604	Leg1	1AJ5	9-Oct-16	0:16	20.58	132.24	500	500																	
286	KY1604	Leg1	1AJ5	9-Oct-16	0:16	20.58	132.24	750	750																	
287	KY1604	Leg1	1AJ5	9-Oct-16	0:16	20.58	132.24	1000	1000																	
288	KY1604	Leg1	1AJ5	9-Oct-16	0:16	20.58	132.24	SCM																		
289	KY1604	Leg1	1AJ6	9-Oct-16	1:41	20.56	132.26	0	0																	
290	KY1604	Leg1	1AJ6	9-Oct-16	1:41	20.56	132.26	10	10																	
291	KY1604	Leg1	1AJ6	9-Oct-16	1:41	20.56	132.26	50	50																	
292	KY1604	Leg1	1AJ6	9-Oct-16	1:41	20.56	132.26	75	75																	
293	KY1604	Leg1	1AJ6	9-Oct-16	1:41	20.56	132.26	100	100																	
294	KY1604	Leg1	1AJ6	9-Oct-16	1:41	20.56	132.26	150	150																	
295	KY1604	Leg1	1AJ6	9-Oct-16	1:41	20.56	132.26	200	200																	
296	KY1604	Leg1	1AJ6	9-Oct-16	1:41	20.56	132.26	300	300																	
297	KY1604	Leg1	1AJ6	9-Oct-16	1:41	20.56	132.26	500	500																	
298	KY1604	Leg1	1AJ6	9-Oct-16	1:41	20.56	132.26	750	750																	
299	KY1604	Leg1	1AJ6	9-Oct-16	1:41	20.56	132.26	1000	1000																	
300	KY1604	Leg1	1AJ6	9-Oct-16	1:41	20.56	132.26	SCM																		
301	KY1604	Leg1	1AJ7	9-Oct-16	3:07	20.55	132.28	0	0																	
302	KY1604	Leg1	1AJ7	9-Oct-16	3:07	20.55	132.28	10	10																	
303	KY1604	Leg1	1AJ7	9-Oct-16	3:07	20.55	132.28	50	50																	
304	KY1604	Leg1	1AJ7	9-Oct-16	3:07	20.55	132.28	75	75																	
305	KY1604	Leg1	1AJ7	9-Oct-16	3:07	20.55	132.28	100	100																	
306	KY1604	Leg1	1AJ7	9-Oct-16	3:07	20.55	132.28	150	150																	
307	KY1604	Leg1	1AJ7	9-Oct-16	3:07	20.55	132.28	200	200																	
308	KY1604	Leg1	1AJ7	9-Oct-16	3:07	20.55	132.28	300	300																	
309	KY1604	Leg1	1AJ7	9-Oct-16	3:07	20.55	132.28	500	500																	
310	KY1604	Leg1	1AJ7	9-Oct-16	3:07	20.55	132.28	750	750																	
311	KY1604	Leg1	1AJ7	9-Oct-16	3:07	20.55	132.28	1000	1000																	
312	KY1604	Leg1	1AJ7	9-Oct-16	3:07	20.55	132.28	SCM																		
313	KY1604	Leg1	1AJ8	9-Oct-16	4:22	20.53	132.30	0	0																	
314	KY1604	Leg1	1AJ8	9-Oct-16	4:22	20.53	132.30	10	10																	
315	KY1604	Leg1	1AJ8	9-Oct-16	4:22	20.53	132.30	50	50																	
316	KY1604	Leg1	1AJ8	9-Oct-16	4:22	20.53	132.30	75	75																	
317	KY1604	Leg1	1AJ8	9-Oct-16	4:22	20.53	132.30	100	100																	
318	KY1604	Leg1	1AJ8	9-Oct-16	4:22	20.53	132.30	150	150																	
319	KY1604	Leg1	1AJ8	9-Oct-16	4:22	20.53	132.30	200	200																	
320	KY1604	Leg1	1AJ8	9-Oct-16	4:22	20.53	132.30	300	300																	
321	KY1604	Leg1	1AJ8	9-Oct-16	4:22	20.53	132.30	500	500																	
322	KY1604	Leg1	1AJ8	9-Oct-16	4:22	20.53	132.30	750	750																	
323	KY1604	Leg1	1AJ8	9-Oct-16	4:22	20.53	132.30	1000	1000																	
324	KY1604	Leg1	1AJ8	9-Oct-16	4:22	20.53	132.30	SCM																		
325	KY1604	Leg2	2A1	19-Oct-16	16:56	25.00	131.00	0	0	29.4					0.02	0.00	1.44	-0.03	0.04							
326	KY1604	Leg2	2A1	19-Oct-16	16:56	25.00	131.00	10	10	29.1	34.7	21.8	6.0	0.04	0.13	0.00	1.50	-0.04	0.04							
327	KY1604	Leg2	2A1	19-Oct-16	16:56	25.00	131.00	50	50	28.3	34.6	22.0	6.2	0.06	0.00	-0.01	1.75	-0.04	0.07							
328	KY1604	Leg2	2A1	19-Oct-16	16:56	25.00	131.00	75	75	24.3	34.8	23.4	6.8	0.10												
329	KY1604	Leg2	2A1	19-Oct-16	16:56	25.00	131.00	100	100	21.7	34.9	24.2	6.5	0.17	-0.01	0.00	2.06	-0.04	0.10							
330	KY1604	Leg2	2A1	19-Oct-16	16:56	25.00	131.00	150	150	19.6	34.9	24.8	5.9	0.07												
331	KY1604	Leg2	2A1	19-Oct-16	16:56	25.00	131.00	200	200	18.6	34.8	25.0	5.9	0.02	5.05	0.00	4.57	0.30	0.01							
332	KY1604	Leg2	2A1	19-Oct-16	16:56	25.00	131.00	300	300	16.7	34.7	25.4	6.0	0.02	6.86	-0.01	6.73	0.63								
333	KY1604	Leg2	2A1	19-Oct-16	16:56	25.00	131.00	500	500	11.4	34.3	26.2	5.1	0.02												
334	KY1604	Leg2	2A1	19-Oct-16	16:56	25.00	131.00	750	750	6.2	34.2	26.9	2.9	0.03												
335	KY1604	Leg2	2A1	19-Oct-16	16:56	25.00	131.00	1000	1000	4.1	34.3	27.3	1.8	0.03												
336	KY1604	Leg2	2A1	19-Oct-16	16:56	25.00	131.00	SCM	111	20.9	34.9	24.4	6.4	0.25	0.65	0.04	2.61	0.06	0.23	0.01	0.02	0.20				
337	KY1604	Leg2	2A2	19-Oct-16	23:06	24.50	131.00	0	0	29.1	34.7															
338	KY1604	Leg2	2A2	19-Oct-16	23:06	24.50	131.00	10	10	28.9	34.7	21.9	6.0	0.05												
339	KY1604	Leg2	2A2	19-Oct-16	23:06	24.50	131.00	50	50	26.8	34.8	22.6	6.3	0.07												
340	KY1604	Leg2	2A2	19-Oct-16	23:06	24.50	131.00	75	75	24.3	34.9	23.5	6.3	0.16												
341	KY1604	Leg2	2A2	19-Oct-16	23:06	24.50	131.00	100	100	23.0	34.9	23.9	6.1	0.24												
342	KY1604	Leg2	2A2	19-Oct-16	23:06	24.50	131.00	150	150	20.8	34.9	24.5	5.8	0.04												
343	KY1604	Leg2	2A2	19-Oct-16	23:06	24.50	131.00	200	200	19.1	34.8	24.9	5.8	0.02												
344	KY1604	Leg2	2A2	19-Oct-16	23:06	24.50	131.00	300	300	16.7	34.7	25.3	6.0	0.02												
345	KY1604	Leg2	2A2	19-Oct-16	23:06	24.50	131.00	500	500	10.7	34.3	26.3	5.0	0.02												
346	KY1604	Leg2	2A2	19-Oct-16	23:06	24.50	131.00	750	750	6.0	34.2	26.9	2.7	0.03												
347	KY1604	Leg2	2A2	19-Oct-16	23:06	24.50	131.00	1000	1000	4.0	34.3	27.3	1.7	0.03												
348	KY1604	Leg2	2A2	19-Oct-16	23:06	24.50	131.00	SCM	90	23.5	34.9	23.7	6.1	0.27												
349	KY1604	Leg2	2A3	20-Oct-16	3:40	24.02	131.02	0	0	29.2	34.6				0.16	0.03	1.82	0.03	0.07							
350	KY1604	Leg2	2A3	20-Oct-16	3:40	24.02	131.02	10	10	29.2	34.6	21.7	6.0	0.04	0.37	0.03	1.70	0.04	0.06							
351	KY1604	Leg2	2A3	20-Oct-16	3:40	24.02	131.02	50	50	29.2	34.6	21.7	6.0	0.05	0.12	0.02	1.79	0.01	0.08							
352	KY1604	Leg2	2A3	20-Oct-16	3:40	24.02	131.02	75	75	28.3	34.7	22.1	6.2	0.06												
353	KY1604	Leg2	2A3	20-Oct-16	3:40	24.02	131.02	100	100	25.5	34.9	23.1	6.4	0.12	0.35	0.02	2.01	0.03	0.23							
354	KY1604	Leg2	2A3	20-Oct-16	3:40	24.02	131.02	150	150	22.1	35.0	24.2	6.0	0.12												
355	KY1604	Leg2	2A3	20-Oct-16	3:40	24.02	131.02	200	200	20.4	34.9	24.6	5.9	0.03	3.37	0.03	3.42	0.22	0.02							
356	KY1604	Leg2	2A3	20-Oct-16	3:40	24.02	131.02	300	300	17.6	34.7	25.2	5.8	0.02	6.91	0.01	6.92	0.44								
357	KY1604	Leg2	2A3	20-Oct-16	3:40	24.02	131.02	500	500	11.4	34.3	26.2	5.1	0.02												
358	KY1604	Leg2	2A3	20-Oct-16	3:40	24.02	131.02	750	750	5.9	34.2	26.9	2.7	0.03												
359	KY1604	Leg2	2A3	20-Oct-16	3:40	24.02	131.02	1000	1000	4.1	34.4	27.3	1.7	0.03												
360	KY1604	Leg2	2A3	20-Oct-16	3:40	24.02	131.02	SCM	118	23.5	34.9	23.7	6.1	0.18	0.35	0.10	2.08	0.05	0.28	0.02	0.02	0.21				

Supplementary Table 1. Continued 3

No	Cruise	Leg	Station	Date	Time	Latitude [°N]	Longitude [°E]	Layer	Depth [m]	Temperature [°C]	Salinity [psu]	Sigma-t [kg/m^3]	DO [mL/L]	FISP [µg/L]	NO3 [µM]	NO2 [µM]	Si [µM]	PO4 [µM]	Chl-a [µg/L]	Chl-a >10 µm [µg/L]	Chl-a 3-10 µm [µg/L]	Chl-a 0.2-3 µm [µg/L]	Picoeukaryotes [cells/mL]	Synechococcus [cells/mL]	Dinoflagellate [cells/L]	Other phytoplankton [cells/L]
361	KY1604	Leg2	2A5	20-Oct-16	17:00	23.00	131.00	0	0	29.2					0.07	-0.01	1.39	0.00	0.04							
362	KY1604	Leg2	2A5	20-Oct-16	17:00	23.00	131.00	10	10	28.9	34.7	21.9	6.0	0.03	3.84	0.00	1.23	-0.02	0.04							
363	KY1604	Leg2	2A5	20-Oct-16	17:00	23.00	131.00	50	50	28.2	34.7	22.1	6.2	0.05	0.39	-0.01	1.25	-0.01	0.05							
364	KY1604	Leg2	2A5	20-Oct-16	17:00	23.00	131.00	75	75	26.1	34.9	22.9	6.5	0.07												
365	KY1604	Leg2	2A5	20-Oct-16	17:00	23.00	131.00	100	100	24.5	35.2	23.6	5.9	0.15	0.05	0.02	1.61	0.04	0.25							
366	KY1604	Leg2	2A5	20-Oct-16	17:00	23.00	131.00	150	150	21.1	35.0	24.5	5.9	0.13												
367	KY1604	Leg2	2A5	20-Oct-16	17:00	23.00	131.00	200	200	19.1	34.9	24.9	5.9	0.04	3.76	0.01	3.33	0.23	0.01							
368	KY1604	Leg2	2A5	20-Oct-16	17:00	23.00	131.00	300	300	16.6	34.7	25.4	6.1	0.02	6.78	-0.01	6.36	0.44								
369	KY1604	Leg2	2A5	20-Oct-16	17:00	23.00	131.00	500	500	10.8	34.3	26.2	5.1	0.02												
370	KY1604	Leg2	2A5	20-Oct-16	17:00	23.00	131.00	750	750	5.8	34.2	26.9	2.7	0.03												
371	KY1604	Leg2	2A5	20-Oct-16	17:00	23.00	131.00	1000	1000	4.0	34.4	27.3	1.7	0.03												
372	KY1604	Leg2	2A5	20-Oct-16	17:00	23.00	131.00	SCM	100	24.5	35.2	23.6	5.9	0.15	0.10	0.02	1.72	0.06	0.22	0.02	0.02	0.20				
373	KY1604	Leg2	2A6	20-Oct-16	22:31	22.50	131.00	0	0	28.7	34.8															
374	KY1604	Leg2	2A6	20-Oct-16	22:31	22.50	131.00	10	10	28.8	34.8	21.9	6.0	0.03												
375	KY1604	Leg2	2A6	20-Oct-16	22:31	22.50	131.00	50	50	27.9	34.7	22.2	6.2	0.04												
376	KY1604	Leg2	2A6	20-Oct-16	22:31	22.50	131.00	75	75	26.5	34.8	22.8	6.4	0.06												
377	KY1604	Leg2	2A6	20-Oct-16	22:31	22.50	131.00	100	100	23.9	35.1	23.7	6.7	0.09												
378	KY1604	Leg2	2A6	20-Oct-16	22:31	22.50	131.00	150	150	21.3	35.0	24.4	6.0	0.14												
379	KY1604	Leg2	2A6	20-Oct-16	22:31	22.50	131.00	200	200	19.3	35.0	24.9	5.7	0.03												
380	KY1604	Leg2	2A6	20-Oct-16	22:31	22.50	131.00	300	300	16.3	34.7	25.4	6.1	0.02												
381	KY1604	Leg2	2A6	20-Oct-16	22:31	22.50	131.00	500	500	10.1	34.2	26.3	4.8	0.02												
382	KY1604	Leg2	2A6	20-Oct-16	22:31	22.50	131.00	750	750	5.5	34.2	27.0	2.2	0.03												
383	KY1604	Leg2	2A6	20-Oct-16	22:31	22.50	131.00	1000	1000	4.0	34.4	27.3	1.7	0.03												
384	KY1604	Leg2	2A6	20-Oct-16	22:31	22.50	131.00	SCM	122	22.7	35.1	24.1	6.3	0.14												
385	KY1604	Leg2	2A7	21-Oct-16	3:00	22.03	131.02	0	0	28.9	34.8				-0.09	-0.01	1.49	-0.03								
386	KY1604	Leg2	2A7	21-Oct-16	3:00	22.03	131.02	10	10	28.8	34.8	22.0	6.0	0.03	-0.02	-0.01	1.33	-0.04	0.03							
387	KY1604	Leg2	2A7	21-Oct-16	3:00	22.03	131.02	50	50	28.0	34.7	22.2	6.2	0.04	-0.05	-0.01	1.44	-0.03	0.05							
388	KY1604	Leg2	2A7	21-Oct-16	3:00	22.03	131.02	75	75	25.2	35.0	23.3	6.8	0.07												
389	KY1604	Leg2	2A7	21-Oct-16	3:00	22.03	131.02	100	100	23.6	35.0	23.8	6.6	0.11	-0.02	-0.01	1.58	-0.01	0.20							
390	KY1604	Leg2	2A7	21-Oct-16	3:00	22.03	131.02	150	150	20.9	35.1	24.6	5.6	0.09												
391	KY1604	Leg2	2A7	21-Oct-16	3:00	22.03	131.02	200	200	18.6	34.9	25.0	5.5	0.02	4.81	-0.01	3.99	0.35	0.01							
392	KY1604	Leg2	2A7	21-Oct-16	3:00	22.03	131.02	300	300	15.1	34.5	25.6	5.6	0.02	9.75	0.00	9.26	0.67								
393	KY1604	Leg2	2A7	21-Oct-16	3:00	22.03	131.02	500	500	9.3	34.2	26.4	4.6	0.02												
394	KY1604	Leg2	2A7	21-Oct-16	3:00	22.03	131.02	750	750	5.1	34.2	27.1	2.0	0.03												
395	KY1604	Leg2	2A7	21-Oct-16	3:00	22.03	131.02	1000	1000	3.9	34.4	27.3	1.8	0.03												
396	KY1604	Leg2	2A7	21-Oct-16	3:00	22.03	131.02	SCM	120	22.3	35.1	24.2	6.1	0.16	0.02	0.01	1.69	0.01	0.21	0.01	0.01	0.20				
397	KY1604	Leg2	2T1	21-Oct-16	22:24	22.01	130.58	0	0																	
398	KY1604	Leg2	2T1	21-Oct-16	22:24	22.01	130.58	10	10	28.9		21.9	6.3													
399	KY1604	Leg2	2T1	21-Oct-16	22:24	22.01	130.58	50	50	28.7		22.0	6.3													
400	KY1604	Leg2	2T1	21-Oct-16	22:24	22.01	130.58	75	75	27.2		22.5	6.7													
401	KY1604	Leg2	2T1	21-Oct-16	22:24	22.01	130.58	100	100	24.5		23.5	7.2													
402	KY1604	Leg2	2T1	21-Oct-16	22:24	22.01	130.58	150	150																	
403	KY1604	Leg2	2T1	21-Oct-16	22:24	22.01	130.58	200	200																	
404	KY1604	Leg2	2T1	21-Oct-16	22:24	22.01	130.58	300	300																	
405	KY1604	Leg2	2T1	21-Oct-16	22:24	22.01	130.58	500	500																	
406	KY1604	Leg2	2T1	21-Oct-16	22:24	22.01	130.58	750	750																	
407	KY1604	Leg2	2T1	21-Oct-16	22:24	22.01	130.58	1000	1000																	
408	KY1604	Leg2	2T1	21-Oct-16	22:24	22.01	130.58	SCM																		
409	KY1604	Leg2	2T2	21-Oct-16	23:44	22.02	130.54	0	0																	
410	KY1604	Leg2	2T2	21-Oct-16	23:44	22.02	130.54	10	10	28.9		21.9	6.3													
411	KY1604	Leg2	2T2	21-Oct-16	23:44	22.02	130.54	50	50	28.7		22.0	6.4													
412	KY1604	Leg2	2T2	21-Oct-16	23:44	22.02	130.54	75	75	26.9		22.6	6.8													
413	KY1604	Leg2	2T2	21-Oct-16	23:44	22.02	130.54	100	100	24.5		23.5	7.2													
414	KY1604	Leg2	2T2	21-Oct-16	23:44	22.02	130.54	150	150																	
415	KY1604	Leg2	2T2	21-Oct-16	23:44	22.02	130.54	200	200																	
416	KY1604	Leg2	2T2	21-Oct-16	23:44	22.02	130.54	300	300																	
417	KY1604	Leg2	2T2	21-Oct-16	23:44	22.02	130.54	500	500																	
418	KY1604	Leg2	2T2	21-Oct-16	23:44	22.02	130.54	750	750																	
419	KY1604	Leg2	2T2	21-Oct-16	23:44	22.02	130.54	1000	1000																	
420	KY1604	Leg2	2T2	21-Oct-16	23:44	22.02	130.54	SCM																		
421	KY1604	Leg2	2A10	22-Oct-16	22:07	20.50	131.00	0	0	28.9	34.7															
422	KY1604	Leg2	2A10	22-Oct-16	22:07	20.50	131.00	10	10	28.8	34.7	21.9	6.1	0.04												
423	KY1604	Leg2	2A10	22-Oct-16	22:07	20.50	131.00	50	50	27.2	34.8	22.5	6.3	0.05												
424	KY1604	Leg2	2A10	22-Oct-16	22:07	20.50	131.00	75	75	24.5	34.9	23.5	6.3	0.09												
425	KY1604	Leg2	2A10	22-Oct-16	22:07	20.50	131.00	100	100	22.3	35.0	24.1	6.2	0.20												
426	KY1604	Leg2	2A10	22-Oct-16	22:07	20.50	131.00	150	150	20.0	34.9	24.7	5.9	0.10												
427	KY1604	Leg2	2A10	22-Oct-16	22:07	20.50	131.00	200	200	18.2	34.8	25.1	6.0	0.02												
428	KY1604	Leg2	2A10	22-Oct-16	22:07	20.50	131.00	300	300	16.2	34.6	25.4	6.0	0.02												
429	KY1604	Leg2	2A10	22-Oct-16	22:07	20.50	131.00	500	500	9.3	34.2	26.4	4.6	0.02												
430	KY1604	Leg2	2A10	22-Oct-16	22:07	20.50	131.00	750	750	5.2	34.3	27.1	2.0	0.03												
431	KY1604	Leg2	2A10	22-Oct-16	22:07	20.50	131.00	1000	1000	3.9	34.5	27.4	2.0	0.03												
432	KY1604	Leg2	2A10	22-Oct-16	22:07	20.50	131.00	SCM	117	21.5	35.0	24.3	6.0	0.18												
433	KY1604	Leg2	2A9	22-Oct-16	16:59	21.00	131.00	0	0	29.2					-0.04	-0.01	1.14	-0.03	0.04							
434	KY1604	Leg2	2A9	22-Oct-16	16:59	21.00	131.00	10	10	29.3	34.7	21.7	6.0	0.04	-0.03	-0.01	1.28	-0.03	0.04							
435	KY1604	Leg2	2A9	22-Oct-16	16:59	21.00	131.00	50	50	28.4	34.8	22.1	6.0	0.05	0.03	-0.01	1.27	-0.02	0.05							
436	KY1604	Leg2	2A9	22-Oct-16	16:59	21.00	131.00	75	75	26.3	34.9	22.9	6.5	0.08												
437	KY1604	Leg2	2A9	22-Oct-16	16:59	21.00	131.00	100	100	22.9	35.0	23.9	6.3	0.16	-0.05	-0.01	1.63	-0.01	0.18							
438	KY1604	Leg2	2A9	22-Oct-16	16:59	21.00	131.00	150	150	19.9	34.9	24.7	6.2	0.11												
439	KY1604	Leg2	2A9	22-Oct-16	16:59	21.00	131.00	200	200	19.0	34.9	24.9	6.3	0.03	2.55	0.00	2.69	0.16	0.01							
440	KY1604	Leg2	2A9	22-Oct-16	16:59	21.00	131.00	300	300	17.0	34.7	25.3	6.1	0.02	6.28	0.00	5.51	0.42								
441	KY1604	Leg2	2A9	22-Oct-16	16:59	21.00	131.00	500	500	9.9	34.2	26.4	4.9	0.02												
442	KY1604	Leg2	2A9	22-Oct-16	16:59	21.00	131.00	750	750	5.2	34.2	27.0	2.1	0.03												
443	KY1604	Leg2	2A9	22-Oct-16	16:59	21.00	131.00	1000	1000	3.8	34.4	27.4	1.8	0.03												
444	KY1604	Leg2	2A9	22-Oct-16	16:59	21.00	131.00	SCM	118	22.0	35.0	24.2	6.0	0.26	0.01	0.03	2.07	0.01	0.28	0.02	0.02	0.23				
445	KY1604	Leg2	2T3	22-Oct-16	0:59	22.02	130.51	0	0																	
446	KY1604	Leg2	2T3	22-Oct-16	0:59	22.02	130.51	10	10	29.0		21.8	6.3													
447	KY1604	Leg2	2T3	22-Oct-16	0:59	22.02	130.51	50	50	28.3		22.1	6.5													
448	KY1604	Leg2	2T3	22-Oct-16	0:59	22.02	130.51	75	75	26.4		22.8	6.8													
449	KY1604	Leg2	2T3	22-Oct-16	0:59	22.02	130.51	100	100																	
450	KY1604	Leg2	2T3	22-Oct-16	0:59	22.02	130.51	150	150																	
451	KY1604	Leg2	2T3	22-Oct-16	0:59	22.02	130.51	200	200																	
452	KY1604	Leg2	2T3	22-Oct-16	0:59	22.02	130.51	300	300																	
453	KY1604	Leg2	2T3	22-Oct-16	0:59	22.02	130.51	500	500																	
454	KY1604	Leg2	2T3	22-Oct-16	0:59	22.02	130.51	750	750																	
455	KY1604	Leg2	2T3	22-Oct-16	0:59	22.02	130.51	1000	1000																	
456	KY1604	Leg2	2T3	22-Oct-16	0:59	22.02	130.51	SCM																		
457	KY1604	Leg2	2T4	22-Oct-16	1:59	22.03	130.48	0	0																	
458	KY1604	Leg2	2T4	22-Oct-16	1:59	22.03	130.48	10	10	29.2		21.7	6.3													
459	KY1604	Leg2	2T4	22-Oct-16	1:59	22.03	130.48	50	50	27.9		22.3	6.6													
460	KY1604	Leg2	2T4	22-Oct-16	1:59	22.03	130.48	75	75	25.3		23.2	7.0													
461	KY1604	Leg2	2T4	22-Oct-16	1:59	22.03	130.48	100	100																	
462	KY1604	Leg2	2T4	22-Oct-16	1:59	22.03	130.48	150	150																	
463	KY1604	Leg2	2T4	22-Oct-16	1:59	22.03	130.48	200	200																	
464	KY1604	Leg2	2T4	22-Oct-16	1:59	22.03	130.48	300	300																	
465	KY1604	Leg2	2T4	22-Oct-16	1:59	22.03	130.48	500	500																	
466	KY1604	Leg2	2T4	22-Oct-16	1:59	22.03	130.48	750	750																	
467	KY1604	Leg2	2T4	22-Oct-16	1:59	22.03	130.48	1000	1000																	
468	KY1604	Leg2	2T4	22-Oct-16	1:59	22.03	130.48	SCM																		
469	KY1604	Leg2	2T5	22-Oct-16	2:57	22.03	130.50	0	0																	
470	KY1604	Leg2	2T5	22-Oct-16	2:57	22.03	130.50	10	10	29.0		21.8	6.3													
471	KY1604	Leg2	2T5	22-Oct-16	2:57	22.03	130.50	50	50	28.3		22.1	6.5													
472	KY1604	Leg2	2T5	22-Oct-16	2:57	22.03	130.50	75	75	25.9		23.0	6.9													
473	KY1604	Leg2	2T5	22-Oct-16	2:57	22.03	130.50	100	100																	
474	KY1604	Leg2	2T5	22-Oct-16	2:57	22.03	130.50	150	150																	
475	KY1604	Leg2	2T5	22-Oct-16	2:57	22.03	130.50	200	200																	
476	KY1604	Leg2	2T5	22-Oct-16	2:57	22.03	130.50	300	300																	
477	KY1604	Leg2	2T5	22-Oct-16	2:57	22.03	130.50	500	500																	
478	KY1604	Leg2	2T5	22-Oct-16	2:57	22.03	130.50	750	750																	
479	KY1604	Leg2	2T5	22-Oct-16	2:57	22.03	130.50	1000	1000																	
480	KY1604	Leg2	2T5	22-Oct-16	2:57	22.03	130.50	SCM																		

Supplementary Table 1. Continued 4

No	Cruise	Leg	Station	Date	Time	Latitude [°N]	Longitude [°E]	Layer	Depth [m]	Temperature [°C]	Salinity [psu]	Sigma-t [kg/m^3]	DO [mL/L]	FISP [µg/L]	NO3 [µM]	NO2 [µM]	Si [µM]	PO4 [µM]	Chl-a [µg/L]	Chl-a >10 µm [µg/L]	Chl-a 3-10 µm [µg/L]	Chl-a 0.2-3 µm [µg/L]	Picoeukaryotes [cells/mL]	Synechococcus [cells/mL]	Dinoflagellate [cells/L]	Other phytoplankton [cells/L]
481	KY1604	Leg2	2T6	22-Oct-16	3:47	22.02	130.50	0	0																	
482	KY1604	Leg2	2T6	22-Oct-16	3:47	22.02	130.50	10	10	29.0		21.9	6.3													
483	KY1604	Leg2	2T6	22-Oct-16	3:47	22.02	130.50	50	50	28.3		22.1	6.5													
484	KY1604	Leg2	2T6	22-Oct-16	3:47	22.02	130.50	75	75	25.8		23.1	7.0													
485	KY1604	Leg2	2T6	22-Oct-16	3:47	22.02	130.50	100	100	23.8		23.8	7.1													
486	KY1604	Leg2	2T6	22-Oct-16	3:47	22.02	130.50	150	150																	
487	KY1604	Leg2	2T6	22-Oct-16	3:47	22.02	130.50	200	200																	
488	KY1604	Leg2	2T6	22-Oct-16	3:47	22.02	130.50	300	300																	
489	KY1604	Leg2	2T6	22-Oct-16	3:47	22.02	130.50	500	500																	
490	KY1604	Leg2	2T6	22-Oct-16	3:47	22.02	130.50	750	750																	
491	KY1604	Leg2	2T6	22-Oct-16	3:47	22.02	130.50	1000	1000																	
492	KY1604	Leg2	2T6	22-Oct-16	3:47	22.02	130.50	SCM																		
493	KY1604	Leg2	2A11	23-Oct-16	2:45	20.02	130.98	0	0	28.9	34.7				0.01	0.00	1.23	-0.01	0.05							
494	KY1604	Leg2	2A11	23-Oct-16	2:45	20.02	130.98	10	10	29.3	34.7	21.8	6.0	0.04	-0.01	-0.01	1.12	-0.02	0.05							
495	KY1604	Leg2	2A11	23-Oct-16	2:45	20.02	130.98	50	50	29.2	34.7	21.8	5.9	0.04	0.01	0.00	1.16	-0.02	0.05							
496	KY1604	Leg2	2A11	23-Oct-16	2:45	20.02	130.98	75	75	28.6	34.8	22.1	6.2	0.06												
497	KY1604	Leg2	2A11	23-Oct-16	2:45	20.02	130.98	100	100	26.4	35.1	23.0	6.1	0.09	-0.03	-0.01	1.32	0.02	0.14							
498	KY1604	Leg2	2A11	23-Oct-16	2:45	20.02	130.98	150	150	22.3	35.0	24.1	6.0	0.13												
499	KY1604	Leg2	2A11	23-Oct-16	2:45	20.02	130.98	200	200	19.7	34.9	24.8	6.1	0.07	2.70	0.01	2.54	0.17	0.04							
500	KY1604	Leg2	2A11	23-Oct-16	2:45	20.02	130.98	300	300	16.6	34.7	25.4	6.1	0.02	6.37	0.00	5.63	0.43								
501	KY1604	Leg2	2A11	23-Oct-16	2:45	20.02	130.98	500	500	10.1	34.2	26.3	4.9	0.02												
502	KY1604	Leg2	2A11	23-Oct-16	2:45	20.02	130.98	750	750	5.4	34.3	27.0	2.0	0.03												
503	KY1604	Leg2	2A11	23-Oct-16	2:45	20.02	130.98	1000	1000	4.0	34.5	27.4	2.1	0.03												
504	KY1604	Leg2	2A11	23-Oct-16	2:45	20.02	130.98	SCM	126	24.7	35.2	23.6	5.7	0.24	0.01	0.01	1.41	0.08	0.18	0.01	0.01	0.16				
505	KY1604	Leg2	2A13	23-Oct-16	16:55	19.00	131.00	0	0	30.0					0.01	-0.01	1.13	0.01	0.05							
506	KY1604	Leg2	2A13	23-Oct-16	16:55	19.00	131.00	10	10	29.4	34.7	21.7	6.0	0.04	0.00	0.00	1.14	-0.02	0.04							
507	KY1604	Leg2	2A13	23-Oct-16	16:55	19.00	131.00	50	50	29.3	34.7	21.7	6.0	0.05	-0.04	-0.02	1.12	-0.03	0.05							
508	KY1604	Leg2	2A13	23-Oct-16	16:55	19.00	131.00	75	75	29.2	34.7	21.8	6.0	0.06												
509	KY1604	Leg2	2A13	23-Oct-16	16:55	19.00	131.00	100	100	27.3	35.1	22.7	6.4	0.10	0.01	-0.01	0.90	0.00	0.16							
510	KY1604	Leg2	2A13	23-Oct-16	16:55	19.00	131.00	150	150	24.4	35.2	23.7	5.9	0.15												
511	KY1604	Leg2	2A13	23-Oct-16	16:55	19.00	131.00	200	200	21.2	35.1	24.5	5.4	0.06	1.52	0.02	1.94	0.15	0.06							
512	KY1604	Leg2	2A13	23-Oct-16	16:55	19.00	131.00	300	300	17.0	34.7	25.3	5.2	0.02	9.01	0.00	7.75	0.65								
513	KY1604	Leg2	2A13	23-Oct-16	16:55	19.00	131.00	500	500	9.1	34.2	26.5	4.3	0.02												
514	KY1604	Leg2	2A13	23-Oct-16	16:55	19.00	131.00	750	750	5.6	34.3	27.1	2.0	0.03												
515	KY1604	Leg2	2A13	23-Oct-16	16:55	19.00	131.00	1000	1000	4.1	34.5	27.4	2.3	0.03												
516	KY1604	Leg2	2A13	23-Oct-16	16:55	19.00	131.00	SCM	130	25.7	35.2	23.3	6.0	0.21	1.17	0.03	1.00	0.05	0.23	0.01	0.02	0.15				
517	KY1604	Leg2	2A14	23-Oct-16	21:58	18.50	131.00	0	0	29.3	34.7															
518	KY1604	Leg2	2A14	23-Oct-16	21:58	18.50	131.00	10	10	29.2	34.7	21.8	6.0	0.04												
519	KY1604	Leg2	2A14	23-Oct-16	21:58	18.50	131.00	50	50	29.1	34.7	21.8	6.0	0.05												
520	KY1604	Leg2	2A14	23-Oct-16	21:58	18.50	131.00	75	75	28.8	34.8	22.0	6.1	0.07												
521	KY1604	Leg2	2A14	23-Oct-16	21:58	18.50	131.00	100	100	26.9	35.1	22.8	6.3	0.13												
522	KY1604	Leg2	2A14	23-Oct-16	21:58	18.50	131.00	150	150	24.2	35.2	23.7	5.8	0.13												
523	KY1604	Leg2	2A14	23-Oct-16	21:58	18.50	131.00	200	200	21.2	35.1	24.5	5.5	0.06												
524	KY1604	Leg2	2A14	23-Oct-16	21:58	18.50	131.00	300	300	15.0	34.6	25.6	4.9	0.02												
525	KY1604	Leg2	2A14	23-Oct-16	21:58	18.50	131.00	500	500	7.8	34.2	26.6	3.6	0.03												
526	KY1604	Leg2	2A14	23-Oct-16	21:58	18.50	131.00	750	750	5.2	34.4	27.2	2.0	0.03												
527	KY1604	Leg2	2A14	23-Oct-16	21:58	18.50	131.00	1000	1000	3.9	34.5	27.4	2.4	0.03												
528	KY1604	Leg2	2A14	23-Oct-16	21:58	18.50	131.00	SCM	124	25.7	35.2	23.2	6.0	0.21												
529	KY1604	Leg2	2A15	24-Oct-16	2:25	18.03	130.99	0	0	29.3	34.7				0.05	-0.01	1.08	-0.02	0.05							
530	KY1604	Leg2	2A15	24-Oct-16	2:25	18.03	130.99	10	10	29.3	34.7	21.8	5.9	0.04	-0.07	-0.02	1.10	-0.02	0.04							
531	KY1604	Leg2	2A15	24-Oct-16	2:25	18.03	130.99	50	50	29.2	34.8	21.8	6.0	0.04	0.02	-0.01	0.95	-0.01	0.05							
532	KY1604	Leg2	2A15	24-Oct-16	2:25	18.03	130.99	75	75	29.0	34.8	21.9	6.0	0.06												
533	KY1604	Leg2	2A15	24-Oct-16	2:25	18.03	130.99	100	100	27.8	34.9	22.4	6.2	0.10	-0.03	-0.01	1.08	-0.01	0.15							
534	KY1604	Leg2	2A15	24-Oct-16	2:25	18.03	130.99	150	150	24.7	35.2	23.6	5.9	0.17												
535	KY1604	Leg2	2A15	24-Oct-16	2:25	18.03	130.99	200	200	22.4	35.2	24.2	5.4	0.10	1.75	0.02	2.24	0.19	0.09							
536	KY1604	Leg2	2A15	24-Oct-16	2:25	18.03	130.99	300	300	15.9	34.6	25.5	5.3	0.02	11.22	0.00	10.40	0.85								
537	KY1604	Leg2	2A15	24-Oct-16	2:25	18.03	130.99	500	500	7.9	34.2	26.7	3.2	0.03												
538	KY1604	Leg2	2A15	24-Oct-16	2:25	18.03	130.99	750	750	5.5	34.4	27.2	2.3	0.03												
539	KY1604	Leg2	2A15	24-Oct-16	2:25	18.03	130.99	1000	1000	4.2	34.5	27.4	2.5	0.03												
540	KY1604	Leg2	2A15	24-Oct-16	2:25	18.03	130.99	SCM	129	26.1	35.1	23.1	6.3	0.14	-0.02	-0.01	0.85	-0.01	0.23	0.01	0.01	0.21				
541	KY1604	Leg2	2A17	24-Oct-16	17:00	17.00	131.00	0	0	29.4					-0.04	-0.02	0.90	-0.02	0.05							
542	KY1604	Leg2	2A17	24-Oct-16	17:00	17.00	131.00	10	10	29.4	34.7	21.7	6.0	0.04	0.03	-0.02	1.05	-0.02	0.04							
543	KY1604	Leg2	2A17	24-Oct-16	17:00	17.00	131.00	50	50	29.2	34.7	21.8	6.0	0.05	-0.01	-0.01	0.92	-0.02	0.06							
544	KY1604	Leg2	2A17	24-Oct-16	17:00	17.00	131.00	75	75	28.3	34.9	22.2	6.2	0.08												
545	KY1604	Leg2	2A17	24-Oct-16	17:00	17.00	131.00	100	100	26.4	35.1	23.0	6.2	0.13	0.00	-0.01	1.02	0.01	0.22							
546	KY1604	Leg2	2A17	24-Oct-16	17:00	17.00	131.00	150	150	24.0	35.2	23.8	5.7	0.15												
547	KY1604	Leg2	2A17	24-Oct-16	17:00	17.00	131.00	200	200	20.3	35.0	24.7	5.6	0.03	2.89	0.00	2.75	0.22	0.02							
548	KY1604	Leg2	2A17	24-Oct-16	17:00	17.00	131.00	300	300	15.8	34.6	25.5	5.6	0.02	9.60	-0.01	8.70	0.66								
549	KY1604	Leg2	2A17	24-Oct-16	17:00	17.00	131.00	500	500	7.8	34.2	26.7	2.9	0.03												
550	KY1604	Leg2	2A17	24-Oct-16	17:00	17.00	131.00	750	750	5.5	34.4	27.2	2.3	0.03												
551	KY1604	Leg2	2A17	24-Oct-16	17:00	17.00	131.00	1000	1000	4.4	34.5	27.4	2.4	0.03												
552	KY1604	Leg2	2A17	24-Oct-16	17:00	17.00	131.00	SCM	128	25.4	35.2	23.3	6.0	0.27	0.82	-0.01	0.97	0.04	0.25	0.01	0.01	0.23				
553	KY1604	Leg2	2A18	24-Oct-16	21:50	16.50	131.00	0	0	29.5	34.7															
554	KY1604	Leg2	2A18	24-Oct-16	21:50	16.50	131.00	10	10	29.5	34.7	21.6	6.0	0.04												
555	KY1604	Leg2	2A18	24-Oct-16	21:50	16.50	131.00	50	50	28.9	34.7	21.9	6.0	0.04												
556	KY1604	Leg2	2A18	24-Oct-16	21:50	16.50	131.00	75	75	28.0	34.9	22.3	6.2	0.08												
557	KY1604	Leg2	2A18	24-Oct-16	21:50	16.50	131.00	100	100	26.3	35.1	23.0	6.2	0.12												
558	KY1604	Leg2	2A18	24-Oct-16	21:50	16.50	131.00	150	150	22.9	35.2	24.1	5.6	0.08												
559	KY1604	Leg2	2A18	24-Oct-16	21:50	16.50	131.00	200	200	19.7	35.0	24.8	5.7	0.03												
560	KY1604	Leg2	2A18	24-Oct-16	21:50	16.50	131.00	300	300	15.0	34.6	25.6	4.9	0.02												
561	KY1604	Leg2	2A18	24-Oct-16	21:50	16.50	131.00	500	500	7.8	34.2	26.7	3.0	0.03												
562	KY1604	Leg2	2A18	24-Oct-16	21:50	16.50	131.00	750	750	5.5	34.4	27.2	2.3	0.03												
563	KY1604	Leg2	2A18	24-Oct-16	21:50	16.50	131.00	1000	1000	4.3	34.5	27.4	2.6	0.03												
564	KY1604	Leg2	2A18	24-Oct-16	21:50	16.50	131.00	SCM	115	25.4	35.2	23.4		0.25												
565	KY1604	Leg2	2A19	25-Oct-16	2:00	16.03	131.00	0	0	29.2	34.7				0.02	0.00	0.96	0.04	0.04							
566	KY1604	Leg2	2A19	25-Oct-16	2:00	16.03	131.00	10	10	28.9	34.7	21.8	6.1	0.03	0.03	0.00	0.93	0.06	0.03							
567	KY1604	Leg2	2A19	25-Oct-16	2:00	16.03	131.00	50	50	28.3	34.7	22.1	6.1	0.05	0.01	0.00	0.95	0.06	0.05							
568	KY1604	Leg2	2A19	25-Oct-16	2:00	16.03	131.00	75	75	27.7	34.9	22.4	6.2	0.06												
569	KY1604	Leg2	2A19	25-Oct-16	2:00	16.03	131.00	100	100	25.5	35.2	23.3	5.9	0.12	0.05	0.02	1.20	0.07	0.22							
570	KY1604	Leg2	2A19	25-Oct-16	2:00	16.03	131.00	150	150	22.2	35.2	24.3	5.6	0.08												
571	KY1604	Leg2	2A19	25-Oct-16	2:00	16.03	131.00	200	200	19.0	34.9	25.0	5.7	0.02	3.76	0.00	3.13	0.26	0.01							
572	KY1604	Leg2	2A19	25-Oct-16	2:00	16.03	131.00	300	300	14.9	34.5	25.6	5.6	0.02	10.17	0.00	9.59	0.72								
573	KY1604	Leg2	2A19	25-Oct-16	2:00	16.03	131.00	500	500	7.4	34.2	26.8	2.7	0.03												
574	KY1604	Leg2	2A19	25-Oct-16	2:00	16.03	131.00	750	750	5.3	34.4	27.2	2.3	0.03												
575	KY1604	Leg2	2A19	25-Oct-16	2:00	16.03	131.00	1000	1000	4.3	34.5	27.4	2.6	0.03												
576	KY1604	Leg2	2A19	25-Oct-16	2:00	16.03	131.00	SCM	106	24.9	35.2	23.5	5.9	0.25	0.47	0.06	1.20	0.09	0.23	0.01	0.01					
577	KY1604	Leg2	2A21	25-Oct-16	16:54	15.00	131.00	0	0	28.8					-0.05	0.00	1.01	0.04	0.04							
578	KY1604	Leg2	2A21	25-Oct-16	16:54	15.00	131.00	10	10	28.5	34.8	22.1	6.1	0.04	0.02	-0.01	1.05	0.04	0.04							
579	KY1604	Leg2	2A21	25-Oct-16	16:54	15.00	131.00	50	50	27.8	34.8	22.3	6.1	0.05	-0.01	-0.01	1.04	0.05	0.05							
580	KY1604	Leg2	2A21	25-Oct-16	16:54	15.00	131.00	75	75	26.7	34.9	22.8	6.1	0.11												
581	KY1604	Leg2	2A21	25-Oct-16	16:54	15.00	131.00	100	100	22.1	35.1	24.3	5.8	0.30	1.48	0.03	1.99	0.11	0.27							
582	KY1604	Leg2	2A21	25-Oct-16	16:54	15.00	131.00	150	150	18.7	34.9	25.0	5.8	0.04												
583	KY1604	Leg2	2A21	25-Oct-16	16:54	15.00	131.00	200	200	16.8	34.7	25.3	5.8	0.02	6.81	0.01	5.82	0.44	0.00							
584	KY1604	Leg2	2A21	25-Oct-16	16:54	15.00	131.00	300	300	11.7	34.3	26.1	4.6	0.02	19.54	-0.01	22.53	1.39								
585	KY1604	Leg2	2A21	25-Oct-16	16:54	15.00	131.00	500	500	6.8	34.3	26.9	2.1	0.03												
586	KY1604	Leg2	2A21	25-Oct-16	16:54	15.00	131.00	750	750	5.0	34.5	27.3	2.5	0.03												
587	KY1604	Leg2	2A21	25-Oct-16	16:54	15.00	131.00	1000	1000	4.2	34.5	27.4	2.7	0.03												
588	KY1604	Leg2	2A21	25-Oct-16	16:54	15.00	131.00	SCM	92	23.1	35.2	24.0	5.9	0.39	0.18	0.00	1.53	0.09	0.28	0.02	0.01	0.22				
589	KY1604	Leg2	2A22	25-Oct-16	22:07	14.50	131.00	0	0	29.2	34.6															
590	KY1604	Leg2	2A22	25-Oct-16	22:07	14.50	131.00	10	10	29.1	34.6	21.7	6.1	0.04												
591	KY1604	Leg2	2A22	25-Oct-16	22:07	14.50	131.00	50	50	27.8	34.9	22.4	6.3	0.06												
592	KY1604	Leg2	2A22	25-Oct-16	22:07	14.50	131.00	75	75	25.1	35.2	23.5	6.2	0.11												
593	KY1604	Leg2	2A22	25-Oct-16	22:07	14.50	131.00	100	100	23.0	35.1	24.0	6.0	0.30												
594	KY1604	Leg2	2A22	25-Oct-16	22:07	14.50	131.00	150	150	19.0	34.9	24.9	5.8	0.04												
595	KY1604	Leg2	2A22	25-Oct-16	22:07	14.50	131.00	200	200	16.2	34.6	25.4	5.7	0.02												
596	KY1604	Leg2	2A22	25-Oct-16	22:07	14.50	131.00	300	300	10.9	34.3	26.3	4.1	0.02												
597	KY1604	Leg2	2A22	25-Oct-16	22:07	14.50	131.00	500	500	6.8	34.3	26.9	2.3	0.03												
598	KY1604	Leg2	2A22	25-Oct-16	22:07	14.50	131.00	750	750	5.1	34.5	27.3	2.6	0.03												
599	KY1604	Leg2	2A22	25-Oct-16	22:07	14.50	131.00	1000	1000	4.1	34.5	27.4	2.7	0.03												
600	KY1604	Leg2	2A22	25-Oct-16	22:07	14.50	131.00	SCM	107	22.7	35.1	24.1	5.9	0.29												

Supplementary Table 1. Continued 5

No	Cruise	Leg	Station	Date	Time	Latitude [°N]	Longitude [°E]	Layer	Depth [m]	Temperature [°C]	Salinity [psu]	Sigma-t [kg/m^3]	DO [mL/L]	FISP [μg/L]	NO3 [μM]	NO2 [μM]	Si [μM]	PO4 [μM]	Chl-a [μg/L]	Chl-a >10 μm [μg/L]	Chl-a 3-10 μm [μg/L]	Chl-a 0.2-3 μm [μg/L]	Picoeukaryotes [cells/mL]	Synechococcus [cells/mL]	Dinoflagellate [cells/L]	Other phytoplankton [cells/L]
601	KY1604	Leg2	2A23	26-Oct-16	2:16	14.00	131.00	0	0	29.3	34.5				0.00	-0.01	1.20	0.04	0.04							
602	KY1604	Leg2	2A23	26-Oct-16	2:16	14.00	131.00	10	10	29.3	34.5	21.6	6.0	0.04	0.24	0.00	1.09	0.04	0.04							
603	KY1604	Leg2	2A23	26-Oct-16	2:16	14.00	131.00	50	50	29.1	34.6	21.7	6.0	0.05	0.01	0.00	1.16	0.02	0.06							
604	KY1604	Leg2	2A23	26-Oct-16	2:16	14.00	131.00	75	75	27.7	34.9	22.4	6.3	0.06												
605	KY1604	Leg2	2A23	26-Oct-16	2:16	14.00	131.00	100	100	25.5	35.1	23.3	5.9	0.12	0.07	0.03	1.43	0.10	0.28							
606	KY1604	Leg2	2A23	26-Oct-16	2:16	14.00	131.00	150	150	20.3	35.0	24.7	5.3	0.09												
607	KY1604	Leg2	2A23	26-Oct-16	2:16	14.00	131.00	200	200	14.7	34.5	25.7	5.1	0.02	12.46	0.00	11.86	0.86	0.00							
608	KY1604	Leg2	2A23	26-Oct-16	2:16	14.00	131.00	300	300	11.4	34.4	26.3	3.5	0.02	23.10	0.00	26.67	1.66								
609	KY1604	Leg2	2A23	26-Oct-16	2:16	14.00	131.00	500	500	7.0	34.4	26.9	2.4	0.03												
610	KY1604	Leg2	2A23	26-Oct-16	2:16	14.00	131.00	750	750	5.5	34.5	27.2	2.8	0.03												
611	KY1604	Leg2	2A23	26-Oct-16	2:16	14.00	131.00	1000	1000	4.3	34.5	27.4	2.8	0.03												
612	KY1604	Leg2	2A23	26-Oct-16	2:16	14.00	131.00	SCM	99	25.8	35.2	23.2	6.1	0.12	0.00	0.01	1.44	0.11	0.24	0.01	0.01	0.22				
613	KY1604	Leg2	2B1	26-Oct-16	17:30	14.00	129.00	0	0	29.3					0.01	0.00	1.12	0.04	0.04							
614	KY1604	Leg2	2B1	26-Oct-16	17:30	14.00	129.00	10	10	29.4	34.5	21.6	6.0	0.04	-0.01	0.00	1.17	0.04	0.04							
615	KY1604	Leg2	2B1	26-Oct-16	17:30	14.00	129.00	50	50	29.2	34.5	21.7	6.0	0.05	-0.01	0.01	1.01	-0.01	0.06							
616	KY1604	Leg2	2B1	26-Oct-16	17:30	14.00	129.00	75	75	28.1	34.8	22.2	6.3	0.07												
617	KY1604	Leg2	2B1	26-Oct-16	17:30	14.00	129.00	100	100	26.3	35.1	23.0	6.1	0.12	0.04	0.00	1.12	0.05	0.17							
618	KY1604	Leg2	2B1	26-Oct-16	17:30	14.00	129.00	150	150	21.6	35.1	24.4	5.3	0.12												
619	KY1604	Leg2	2B1	26-Oct-16	17:30	14.00	129.00	200	200	17.1	34.7	25.3	5.1	0.03	9.51	0.01	8.29	0.68	0.00							
620	KY1604	Leg2	2B1	26-Oct-16	17:30	14.00	129.00	300	300	11.0	34.3	26.2	4.6	0.02	20.14	0.00	24.69	1.40								
621	KY1604	Leg2	2B1	26-Oct-16	17:30	14.00	129.00	500	500	6.1	34.3	27.0	2.3	0.03												
622	KY1604	Leg2	2B1	26-Oct-16	17:30	14.00	129.00	750	750	4.9	34.5	27.3	2.7	0.03												
623	KY1604	Leg2	2B1	26-Oct-16	17:30	14.00	129.00	1000	1000	4.1	34.6	27.4	2.8	0.03												
624	KY1604	Leg2	2B1	26-Oct-16	17:30	14.00	129.00	SCM	116	25.2	35.2	23.4	5.9	0.25	0.15	0.08	1.36	0.11	0.28	0.01	0.02	0.22				
625	KY1604	Leg2	2B2	26-Oct-16	22:42	14.50	129.00	0	0	29.3	34.6															
626	KY1604	Leg2	2B2	26-Oct-16	22:42	14.50	129.00	10	10	29.3	34.6	21.6	6.0	0.04												
627	KY1604	Leg2	2B2	26-Oct-16	22:42	14.50	129.00	50	50	29.1	34.6	21.7	6.0	0.05												
628	KY1604	Leg2	2B2	26-Oct-16	22:42	14.50	129.00	75	75	28.7	34.7	21.9	6.2	0.06												
629	KY1604	Leg2	2B2	26-Oct-16	22:42	14.50	129.00	100	100	26.5	35.1	22.9	6.1	0.11												
630	KY1604	Leg2	2B2	26-Oct-16	22:42	14.50	129.00	150	150	20.5	35.1	24.7	5.3	0.05												
631	KY1604	Leg2	2B2	26-Oct-16	22:42	14.50	129.00	200	200	16.4	34.7	25.4	5.2	0.02												
632	KY1604	Leg2	2B2	26-Oct-16	22:42	14.50	129.00	300	300	11.1	34.3	26.2	4.8	0.02												
633	KY1604	Leg2	2B2	26-Oct-16	22:42	14.50	129.00	500	500	6.8	34.4	27.0	2.6	0.03												
634	KY1604	Leg2	2B2	26-Oct-16	22:42	14.50	129.00	750	750	5.0	34.5	27.3	2.9	0.03												
635	KY1604	Leg2	2B2	26-Oct-16	22:42	14.50	129.00	1000	1000	3.9	34.6	27.4	2.8	0.03												
636	KY1604	Leg2	2B2	26-Oct-16	22:42	14.50	129.00	SCM	115	24.9	35.1	23.5	5.8	0.28												
637	KY1604	Leg2	2B3	27-Oct-16	2:59	14.98	129.01	0	0	29.3	34.6				-0.04	0.00	1.06	0.03	0.04							
638	KY1604	Leg2	2B3	27-Oct-16	2:59	14.98	129.01	10	10	29.2	34.6	21.7	6.0	0.04	0.44	0.01	0.80	0.01	0.04							
639	KY1604	Leg2	2B3	27-Oct-16	2:59	14.98	129.01	50	50	29.2	34.6	21.7	6.0	0.04	-0.04	0.00	0.93	-0.02	0.04							
640	KY1604	Leg2	2B3	27-Oct-16	2:59	14.98	129.01	75	75	29.2	34.6	21.7	6.0	0.04												
641	KY1604	Leg2	2B3	27-Oct-16	2:59	14.98	129.01	100	100	26.9	35.0	22.8	6.2	0.08	-0.06	0.00	0.93	0.02	0.15							
642	KY1604	Leg2	2B3	27-Oct-16	2:59	14.98	129.01	150	150	23.0	35.2	24.1	5.5	0.14												
643	KY1604	Leg2	2B3	27-Oct-16	2:59	14.98	129.01	200	200	19.2	34.9	24.9	5.3	0.03	5.35	0.00	4.52	0.42	0.01							
644	KY1604	Leg2	2B3	27-Oct-16	2:59	14.98	129.01	300	300	13.1	34.4	25.9	5.0	0.02	16.41	0.01	17.30	1.15								
645	KY1604	Leg2	2B3	27-Oct-16	2:59	14.98	129.01	500	500	7.0	34.3	26.8	2.5	0.03												
646	KY1604	Leg2	2B3	27-Oct-16	2:59	14.98	129.01	750	750	4.9	34.5	27.3	2.5	0.03												
647	KY1604	Leg2	2B3	27-Oct-16	2:59	14.98	129.01	1000	1000	3.8	34.5	27.5	2.7	0.03												
648	KY1604	Leg2	2B3	27-Oct-16	2:59	14.98	129.01	SCM	114	26.0	35.1	23.1	6.1	0.11	0.05	0.03	0.97	0.07	0.22	0.01	0.01					
649	KY1604	Leg2	2C1	27-Oct-16	17:27	16.00	128.00	0	0	28.8					0.00	0.01	1.12	0.04	0.04							
650	KY1604	Leg2	2C1	27-Oct-16	17:27	16.00	128.00	10	10	28.6	34.8	22.1	6.1	0.03	-0.04	0.00	1.12	0.00	0.04				501	928	7845	871
651	KY1604	Leg2	2C1	27-Oct-16	17:27	16.00	128.00	50	50	27.5	34.9	22.5	6.1	0.04	-0.03	0.01	1.03	0.03	0.04				650	1690	3609	568
652	KY1604	Leg2	2C1	27-Oct-16	17:27	16.00	128.00	75	75	27.2	34.9	22.6	6.1	0.08												
653	KY1604	Leg2	2C1	27-Oct-16	17:27	16.00	128.00	100	100	26.5	35.0	22.9	6.1	0.11	0.19	0.01	1.25	0.05	0.15				4630	1285	7246	1655
654	KY1604	Leg2	2C1	27-Oct-16	17:27	16.00	128.00	150	150	20.2	35.0	24.7	5.7	0.05												
655	KY1604	Leg2	2C1	27-Oct-16	17:27	16.00	128.00	200	200	16.7	34.7	25.4	5.4	0.02	7.46	0.01	6.50	0.53	0.00				115	2	1229	0
656	KY1604	Leg2	2C1	27-Oct-16	17:27	16.00	128.00	300	300	12.1	34.4	26.1	4.4	0.02	18.93	-0.01	20.61	1.34								
657	KY1604	Leg2	2C1	27-Oct-16	17:27	16.00	128.00	500	500	7.2	34.3	26.8	2.5	0.03												
658	KY1604	Leg2	2C1	27-Oct-16	17:27	16.00	128.00	750	750	4.6	34.5	27.3	2.3	0.03												
659	KY1604	Leg2	2C1	27-Oct-16	17:27	16.00	128.00	1000	1000	3.7	34.6	27.5	2.7	0.03												
660	KY1604	Leg2	2C1	27-Oct-16	17:27	16.00	128.00	SCM	112	24.9	35.1	23.4	5.9	0.30	0.44	0.00	1.14	0.07	0.25	0.01	0.01	0.22	13788	626	15689	3492
661	KY1604	Leg2	2C2	27-Oct-16	22:21	16.50	128.00	0	0	28.4	34.8															
662	KY1604	Leg2	2C2	27-Oct-16	22:21	16.50	128.00	10	10	28.3	34.8	22.2	6.1	0.04												
663	KY1604	Leg2	2C2	27-Oct-16	22:21	16.50	128.00	50	50	27.2	34.9	22.6	6.1	0.06												
664	KY1604	Leg2	2C2	27-Oct-16	22:21	16.50	128.00	75	75	26.9	34.9	22.7	6.1	0.09												
665	KY1604	Leg2	2C2	27-Oct-16	22:21	16.50	128.00	100	100	24.2	35.1	23.7	5.9	0.22												
666	KY1604	Leg2	2C2	27-Oct-16	22:21	16.50	128.00	150	150	20.0	34.9	24.7	5.8	0.05												
667	KY1604	Leg2	2C2	27-Oct-16	22:21	16.50	128.00	200	200	17.8	34.8	25.2	6.0	0.02												
668	KY1604	Leg2	2C2	27-Oct-16	22:21	16.50	128.00	300	300	14.5	34.5	25.7	5.4	0.02												
669	KY1604	Leg2	2C2	27-Oct-16	22:21	16.50	128.00	500	500	7.5	34.2	26.7	2.8	0.03												
670	KY1604	Leg2	2C2	27-Oct-16	22:21	16.50	128.00	750	750	5.1	34.5	27.2	2.3	0.03												
671	KY1604	Leg2	2C2	27-Oct-16	22:21	16.50	128.00	1000	1000	4.1	34.5	27.4	2.6	0.03												
672	KY1604	Leg2	2C2	27-Oct-16	22:21	16.50	128.00	SCM	90	25.6	35.1	23.2		0.26												
673	KY1604	Leg2	2C3	28-Oct-16	2:41	16.99	128.02	0	0	28.4	34.7				-0.05	-0.02	0.87	0.03	0.04							
674	KY1604	Leg2	2C3	28-Oct-16	2:41	16.99	128.02	10	10	28.5	34.7	22.0	6.1	0.04	-0.09	-0.04	0.72	0.02	0.05							
675	KY1604	Leg2	2C3	28-Oct-16	2:41	16.99	128.02	50	50	28.5	34.7	22.0	6.1	0.04	-0.08	-0.04	0.55	0.01	0.05							
676	KY1604	Leg2	2C3	28-Oct-16	2:41	16.99	128.02	75	75	28.3	34.8	22.1	6.1	0.05												
677	KY1604	Leg2	2C3	28-Oct-16	2:41	16.99	128.02	100	100	27.9	34.8	22.3	6.1	0.06	-0.13	-0.03	0.56	0.00	0.09							
678	KY1604	Leg2	2C3	28-Oct-16	2:41	16.99	128.02	150	150	23.9	35.2	23.8	5.6	0.17												
679	KY1604	Leg2	2C3	28-Oct-16	2:41	16.99	128.02	200	200	20.3	35.0	24.7	5.4	0.03	3.50	-0.04	2.53	0.32	0.02							
680	KY1604	Leg2	2C3	28-Oct-16	2:41	16.99	128.02	300	300	13.7	34.4	25.8	5.2	0.02	13.44	-0.05	13.81	0.92								
681	KY1604	Leg2	2C3	28-Oct-16	2:41	16.99	128.02	500	500	7.6	34.2	26.7	2.9	0.03												
682	KY1604	Leg2	2C3	28-Oct-16	2:41	16.99	128.02	750	750	4.8	34.4	27.2	2.1	0.03												
683	KY1604	Leg2	2C3	28-Oct-16	2:41	16.99	128.02	1000	1000	4.0	34.6	27.4	2.8	0.03												
684	KY1604	Leg2	2C3	28-Oct-16	2:41	16.99	128.02	SCM	140	25.1	35.1	23.4	5.9	0.24	0.17	-0.02	1.56	0.09	0.26	0.01	0.01	0.19				
685	KY1604	Leg2	2C5	28-Oct-16	17:23	18.00	128.00	0	0	29.3					0.11	0.02	1.07	0.03	0.04							
686	KY1604	Leg2	2C5	28-Oct-16	17:23	18.00	128.00	10	10	29.3	34.7	21.7	6.0	0.04	-0.03	0.00	1.10	-0.02	0.04				353	330	2135	341
687	KY1604	Leg2	2C5	28-Oct-16	17:23	18.00	128.00	50	50	29.2	34.7	21.8	6.0	0.05	0.00	0.00	1.03	0.09	0.06				389	536	5867	452
688	KY1604	Leg2	2C5	28-Oct-16	17:23	18.00	128.00	75	75	28.7	34.8	22.0	6.1	0.06												
689	KY1604	Leg2	2C5	28-Oct-16	17:23	18.00	128.00	100	100	26.8	35.0	22.8	6.3	0.10	-0.02	0.00	1.20	0.12	0.19				6489	1275	11214	2250
690	KY1604	Leg2	2C5	28-Oct-16	17:23	18.00	128.00	150	150	21.2	35.0	24.4	5.9	0.13												
691	KY1604	Leg2	2C5	28-Oct-16	17:23	18.00	128.00	200	200	18.6	34.8	25.0	5.9	0.03	4.04	0.01	3.37	0.32	0.01				316	3	2224	426
692	KY1604	Leg2	2C5	28-Oct-16	17:23	18.00	128.00	300	300	15.2	34.6	25.6	5.8	0.02	10.62	0.00	10.76	0.73								
693	KY1604	Leg2	2C5	28-Oct-16	17:23	18.00	128.00	500	500	7.8	34.2	26.7	3.5	0.03												
694	KY1604	Leg2	2C5	28-Oct-16	17:23	18.00	128.00	750	750	5.0	34.4	27.2	2.0	0.03												
695	KY1604	Leg2	2C5	28-Oct-16	17:23	18.00	128.00	1000	1000	3.8	34.5	27.4	2.3	0.03												
696	KY1604	Leg2	2C5	28-Oct-16	17:23	18.00	128.00	SCM	113	24.7	35.0	23.5	6.2	0.14	0.03	0.00	1.31	0.13	0.26	0.01	0.02	0.24	35422	166	11994	3269
697	KY1604	Leg2	2C6	28-Oct-16	22:00	18.48	128.00	0	0	29.0	34.8															
698	KY1604	Leg2	2C6	28-Oct-16	22:00	18.48	128.00	10	10	29.2	34.8	21.8	6.0	0.04												
699	KY1604	Leg2	2C6	28-Oct-16	22:00	18.48	128.00	50	50	28.6	34.7	22.0	6.0	0.05												
700	KY1604	Leg2	2C6	28-Oct-16	22:00	18.48	128.00	75	75	27.9	34.8	22.3	6.1	0.06												
701	KY1604	Leg2	2C6	28-Oct-16	22:00	18.48	128.00	100	100	23.9	35.0	23.7	6.3	0.12												
702	KY1604	Leg2	2C6	28-Oct-16	22:00	18.48	128.00	150	150	20.9	35.0	24.5	5.9	0.06												
703	KY1604	Leg2	2C6	28-Oct-16	22:00	18.48	128.00	200	200	19.1	34.9	24.9	5.9	0.02												
704	KY1604	Leg2	2C6	28-Oct-16	22:00	18.48	128.00	300	300	15.9	34.6	25.5	6.0	0.02												
705	KY1604	Leg2	2C6	28-Oct-16	22:00	18.48	128.00	500	500	8.4	34.2	26.6	3.9	0.03												
706	KY1604	Leg2	2C6	28-Oct-16	22:00	18.48	128.00	750	750	5.2	34.4	27.2	2.0	0.03												
707	KY1604	Leg2	2C6	28-Oct-16	22:00	18.48	128.00	1000	1000	3.8	34.5	27.4	2.3	0.03												
708	KY1604	Leg2	2C6	28-Oct-16	22:00	18.48	128.00	SCM	113	23.2	35.0	23.9		0.26												
709	KY1604	Leg2	2D1	29-Oct-16	17:55	22.50	128.00	0	0	29.0	34.7															
710	KY1604	Leg2	2D1	29-Oct-16	17:55	22.50	128.00	10	10	29.0	34.7	21.8	6.0	0.04												
711	KY1604	Leg2	2D1	29-Oct-16	17:55	22.50	128.00	50	50	29.0	34.7	21.8	6.0	0.05												
712	KY1604	Leg2	2D1	29-Oct-16	17:55	22.50	128.00	75	75	25.7	34.8	22.9	6.6	0.09												
713	KY1604	Leg2	2D1	29-Oct-16	17:55	22.50	128.00	100	100	23.2	34.9	23.8	6.6	0.15												
714	KY1604	Leg2	2D1	29-Oct-16	17:55	22.50	128.00	150	150	20.1	34.9	24.7	6.0	0.13												
715	KY1604	Leg2	2D1	29-Oct-16	17:55	22.50	128.00	200	200	18.7	34.8	25.0	6.0	0.03												
716	KY1604	Leg2	2D1	29-Oct-16	17:55	22.50	128.00	300	300	16.4	34.7	25.4	5.9	0.02												
717	KY1604	Leg2	2D1	29-Oct-16	17:55	22.50	128.00	500	500	10.1	34.2	26.3	4.7	0.02												
718	KY1604	Leg2	2D1	29-Oct-16	17:55	22.50	128.00	750	750	5.4	34.2	27.0	2.2	0.03												
719	KY1604	Leg2	2D1	29-Oct-16	17:55	22.50	128.00	1000	1000	3.8	34.4	27.3	1.8	0.03												
720	KY1604	Leg2	2D1	29-Oct-16	17:55	22.50	128.00	SCM	108	22.2	34.9	24.1		0.36												

Supplementary Table 1. Continued 6

No	Cruise	Leg	Station	Date	Time	Latitude [°N]	Longitude [°E]	Layer	Depth [m]	Temperature [°C]	Salinity [psu]	Sigma-t [kg/m^3]	DO [mL/L]	FISP [μg/L]	NO3 [μM]	NO2 [μM]	Si [μM]	PO4 [μM]	Chl-a [μg/L]	Chl-a >10 μm [μg/L]	Chl-a 3-10 μm [μg/L]	Chl-a 0.2-3 μm [μg/L]	Picoeukaryotes [cells/mL]	Synechococcus [cells/mL]	Dinoflagellate [cells/L]	Other phytoplankton [cells/L]
721	KY1604	Leg2	2D2	29-Oct-16	21:59	22.50	128.47	0	0	29.1	34.6															
722	KY1604	Leg2	2D2	29-Oct-16	21:59	22.50	128.47	10	10	29.0	34.6	21.8	6.0	0.04												
723	KY1604	Leg2	2D2	29-Oct-16	21:59	22.50	128.47	50	50	28.3	34.6	22.0	6.2	0.05												
724	KY1604	Leg2	2D2	29-Oct-16	21:59	22.50	128.47	75	75	24.7	34.8	23.3	6.6	0.10												
725	KY1604	Leg2	2D2	29-Oct-16	21:59	22.50	128.47	100	100	22.1	34.9	24.1	6.6	0.19												
726	KY1604	Leg2	2D2	29-Oct-16	21:59	22.50	128.47	150	150	19.9	34.9	24.7	6.2	0.13												
727	KY1604	Leg2	2D2	29-Oct-16	21:59	22.50	128.47	200	200	18.6	34.8	25.0	5.9	0.03												
728	KY1604	Leg2	2D2	29-Oct-16	21:59	22.50	128.47	300	300	16.3	34.7	25.4	5.8	0.02												
729	KY1604	Leg2	2D2	29-Oct-16	21:59	22.50	128.47	500	500	10.1	34.2	26.3	4.7	0.02												
730	KY1604	Leg2	2D2	29-Oct-16	21:59	22.50	128.47	750	750	5.3	34.2	27.0	2.1	0.03												
731	KY1604	Leg2	2D2	29-Oct-16	21:59	22.50	128.47	1000	1000	3.7	34.4	27.3	1.8	0.03												
732	KY1604	Leg2	2D2	29-Oct-16	21:59	22.50	128.47	SCM	117	21.1	34.9	24.4		0.30												
733	KY1604	Leg2	2D3	30-Oct-16	2:03	22.49	128.97	0	0	29.2	34.7															
734	KY1604	Leg2	2D3	30-Oct-16	2:03	22.49	128.97	10	10	29.0	34.7	21.8	6.0	0.04												
735	KY1604	Leg2	2D3	30-Oct-16	2:03	22.49	128.97	50	50	27.1	34.7	22.5	6.4	0.06												
736	KY1604	Leg2	2D3	30-Oct-16	2:03	22.49	128.97	75	75	23.5	34.9	23.7	6.8	0.08												
737	KY1604	Leg2	2D3	30-Oct-16	2:03	22.49	128.97	100	100	21.8	34.9	24.2	6.5	0.18												
738	KY1604	Leg2	2D3	30-Oct-16	2:03	22.49	128.97	150	150	19.8	34.9	24.7	6.3	0.06												
739	KY1604	Leg2	2D3	30-Oct-16	2:03	22.49	128.97	200	200	18.2	34.8	25.1	5.9	0.02												
740	KY1604	Leg2	2D3	30-Oct-16	2:03	22.49	128.97	300	300	16.3	34.7	25.4	5.9	0.02												
741	KY1604	Leg2	2D3	30-Oct-16	2:03	22.49	128.97	500	500	9.3	34.2	26.5	4.6	0.02												
742	KY1604	Leg2	2D3	30-Oct-16	2:03	22.49	128.97	750	750	5.2	34.3	27.1	2.2	0.03												
743	KY1604	Leg2	2D3	30-Oct-16	2:03	22.49	128.97	1000	1000	3.7	34.4	27.4	1.8	0.03												
744	KY1604	Leg2	2D3	30-Oct-16	2:03	22.49	128.97	SCM	108	21.3	34.9	24.3		0.27												
745	KY1604	Leg2	2D5	30-Oct-16	21:56	22.52	130.01	0	0		34.7															
746	KY1604	Leg2	2D5	30-Oct-16	21:56	22.52	130.01	10	10	29.0		21.9	6.4													
747	KY1604	Leg2	2D5	30-Oct-16	21:56	22.52	130.01	50	50	29.0		21.9	6.3													
748	KY1604	Leg2	2D5	30-Oct-16	21:56	22.52	130.01	75	75	25.3		23.2	7.1													
749	KY1604	Leg2	2D5	30-Oct-16	21:56	22.52	130.01	100	100	22.8		24.0	6.9													
750	KY1604	Leg2	2D5	30-Oct-16	21:56	22.52	130.01	150	150	20.4		24.6	6.6													
751	KY1604	Leg2	2D5	30-Oct-16	21:56	22.52	130.01	200	200	18.6		25.0	5.5													
752	KY1604	Leg2	2D5	30-Oct-16	21:56	22.52	130.01	300	300	16.4		25.4	5.5													
753	KY1604	Leg2	2D5	30-Oct-16	21:56	22.52	130.01	500	500	10.6		26.3	5.3													
754	KY1604	Leg2	2D5	30-Oct-16	21:56	22.52	130.01	750	750	5.4		27.0	2.6													
755	KY1604	Leg2	2D5	30-Oct-16	21:56	22.52	130.01	1000	1000																	
756	KY1604	Leg2	2D5	30-Oct-16	21:56	22.52	130.01	SCM	103	22.7		24.0	6.8													
757	KY1604	Leg2	2D6	30-Oct-16	16:56	23.00	130.00	0	0	29.1	34.7															
758	KY1604	Leg2	2D6	30-Oct-16	16:56	23.00	130.00	10	10	29.0	34.7	21.8	6.0	0.05												
759	KY1604	Leg2	2D6	30-Oct-16	16:56	23.00	130.00	50	50	29.0	34.7	21.9	6.0	0.06												
760	KY1604	Leg2	2D6	30-Oct-16	16:56	23.00	130.00	75	75	23.6	34.9	23.7	6.4	0.14												
761	KY1604	Leg2	2D6	30-Oct-16	16:56	23.00	130.00	100	100	21.9	34.9	24.2	5.9	0.21												
762	KY1604	Leg2	2D6	30-Oct-16	16:56	23.00	130.00	150	150	20.1	34.9	24.7	5.9	0.03												
763	KY1604	Leg2	2D6	30-Oct-16	16:56	23.00	130.00	200	200	18.4	34.8	25.0	6.0	0.02												
764	KY1604	Leg2	2D6	30-Oct-16	16:56	23.00	130.00	300	300	16.3	34.7	25.4	5.9	0.02												
765	KY1604	Leg2	2D6	30-Oct-16	16:56	23.00	130.00	500	500	10.1	34.2	26.3	4.6	0.02												
766	KY1604	Leg2	2D6	30-Oct-16	16:56	23.00	130.00	750	750	5.6	34.2	27.0	2.2	0.03												
767	KY1604	Leg2	2D6	30-Oct-16	16:56	23.00	130.00	1000	1000	3.9	34.4	27.3	1.8	0.03												
768	KY1604	Leg2	2D6	30-Oct-16	16:56	23.00	130.00	SCM	82	22.8	34.9	23.9		0.40												
769	KY1604	Leg2	2D4	31-Oct-16	2:06	22.51	129.53	0	0		34.7															
770	KY1604	Leg2	2D4	31-Oct-16	2:06	22.51	129.53	10	10	28.8		21.9	6.4													
771	KY1604	Leg2	2D4	31-Oct-16	2:06	22.51	129.53	50	50	28.1		22.2	6.6													
772	KY1604	Leg2	2D4	31-Oct-16	2:06	22.51	129.53	75	75	24.2		23.6	6.9													
773	KY1604	Leg2	2D4	31-Oct-16	2:06	22.51	129.53	100	100	21.9		24.3	6.5													
774	KY1604	Leg2	2D4	31-Oct-16	2:06	22.51	129.53	150	150	19.9		24.7	6.4													
775	KY1604	Leg2	2D4	31-Oct-16	2:06	22.51	129.53	200	200	18.6		25.0	6.5													
776	KY1604	Leg2	2D4	31-Oct-16	2:06	22.51	129.53	300	300	16.6		25.4	6.8													
777	KY1604	Leg2	2D4	31-Oct-16	2:06	22.51	129.53	500	500	10.7		26.3	5.4													
778	KY1604	Leg2	2D4	31-Oct-16	2:06	22.51	129.53	750	750																	
779	KY1604	Leg2	2D4	31-Oct-16	2:06	22.51	129.53	1000	1000																	
780	KY1604	Leg2	2D4	31-Oct-16	2:06	22.51	129.53	SCM	98	22.0		24.2	6.5													
781	KY1604	Leg2	2D7	31-Oct-16	17:00	23.50	130.00	0	0	28.8	34.8				0.61	0.00	1.08	0.04	0.05							
782	KY1604	Leg2	2D7	31-Oct-16	17:00	23.50	130.00	10	10	28.8	34.7	22.0	6.0	0.04	-0.03	-0.01	1.09	0.02	0.05				397	486	6629	823
783	KY1604	Leg2	2D7	31-Oct-16	17:00	23.50	130.00	50	50	28.1	34.7	22.1	6.2	0.06	-0.03	0.00	1.00	0.02	0.11				513	779	10833	634
784	KY1604	Leg2	2D7	31-Oct-16	17:00	23.50	130.00	75	75	25.8	34.9	23.1	6.5	0.11												
785	KY1604	Leg2	2D7	31-Oct-16	17:00	23.50	130.00	100	100	22.7	35.0	24.0	6.4	0.16	0.06	0.03	1.46	0.03	0.24				28305	238	12458	1520
786	KY1604	Leg2	2D7	31-Oct-16	17:00	23.50	130.00	150	150	20.4	35.0	24.6	5.9	0.09												
787	KY1604	Leg2	2D7	31-Oct-16	17:00	23.50	130.00	200	200	18.8	34.9	25.0	5.7	0.02	4.10	0.01	3.44	0.27	0.01				65	0	1453	377
788	KY1604	Leg2	2D7	31-Oct-16	17:00	23.50	130.00	300	300	16.5	34.7	25.4	6.0	0.02	6.95	0.00	6.08	0.37								
789	KY1604	Leg2	2D7	31-Oct-16	17:00	23.50	130.00	500	500	10.4	34.2	26.3	4.8	0.02												
790	KY1604	Leg2	2D7	31-Oct-16	17:00	23.50	130.00	750	750	5.6	34.2	27.0	2.2	0.03												
791	KY1604	Leg2	2D7	31-Oct-16	17:00	23.50	130.00	1000	1000	3.9	34.4	27.3	1.8	0.03												
792	KY1604	Leg2	2D7	31-Oct-16	17:00	23.50	130.00	SCM	100	22.7	35.0	24.0	6.4	0.16	-0.01	0.00	1.48	-0.01	0.24	0.01	0.01	0.22	26655	480	5560	712
793	KY1604	Leg2	2D8	31-Oct-16	21:32	23.52	129.56	0	0	28.9	34.7															
794	KY1604	Leg2	2D8	31-Oct-16	21:32	23.52	129.56	10	10	28.9	34.7	21.9	6.0	0.04												
795	KY1604	Leg2	2D8	31-Oct-16	21:32	23.52	129.56	50	50	28.2	34.7	22.1	6.3	0.05												
796	KY1604	Leg2	2D8	31-Oct-16	21:32	23.52	129.56	75	75	25.3	34.8	23.1	6.7	0.07												
797	KY1604	Leg2	2D8	31-Oct-16	21:32	23.52	129.56	100	100	22.2	34.9	24.1	6.3	0.17												
798	KY1604	Leg2	2D8	31-Oct-16	21:32	23.52	129.56	150	150	19.8	34.9	24.7	5.9	0.04												
799	KY1604	Leg2	2D8	31-Oct-16	21:32	23.52	129.56	200	200	18.5	34.8	25.0	5.9	0.02												
800	KY1604	Leg2	2D8	31-Oct-16	21:32	23.52	129.56	300	300	16.4	34.7	25.4	5.9	0.02												
801	KY1604	Leg2	2D8	31-Oct-16	21:32	23.52	129.56	500	500	10.4	34.3	26.3	4.8	0.02												
802	KY1604	Leg2	2D8	31-Oct-16	21:32	23.52	129.56	750	750	5.4	34.2	27.0	2.2	0.03												
803	KY1604	Leg2	2D8	31-Oct-16	21:32	23.52	129.56	1000	1000	3.6	34.4	27.3	1.8	0.03												
804	KY1604	Leg2	2D8	31-Oct-16	21:32	23.52	129.56	SCM	105	21.9	34.9	24.2		0.27												
805	KY1604	Leg2	2D9	1-Nov-16	1:39	23.51	129.03	0	0	29.0	34.6															
806	KY1604	Leg2	2D9	1-Nov-16	1:39	23.51	129.03	10	10	28.8	34.6	21.9	6.0	0.04												
807	KY1604	Leg2	2D9	1-Nov-16	1:39	23.51	129.03	50	50	28.0	34.7	22.2	6.4	0.06												
808	KY1604	Leg2	2D9	1-Nov-16	1:39	23.51	129.03	75	75	24.1	34.9	23.5	6.5	0.10												
809	KY1604	Leg2	2D9	1-Nov-16	1:39	23.51	129.03	100	100	21.9	34.9	24.2	6.3	0.16												
810	KY1604	Leg2	2D9	1-Nov-16	1:39	23.51	129.03	150	150	19.1	34.9	24.9	5.8	0.04												
811	KY1604	Leg2	2D9	1-Nov-16	1:39	23.51	129.03	200	200	18.1	34.8	25.1	6.0	0.03												
812	KY1604	Leg2	2D9	1-Nov-16	1:39	23.51	129.03	300	300	16.2	34.7	25.4	5.8	0.02												
813	KY1604	Leg2	2D9	1-Nov-16	1:39	23.51	129.03	500	500	10.0	34.3	26.4	4.5	0.02												
814	KY1604	Leg2	2D9	1-Nov-16	1:39	23.51	129.03	750	750	5.4	34.2	27.0	2.3	0.03												
815	KY1604	Leg2	2D9	1-Nov-16	1:39	23.51	129.03	1000	1000	3.8	34.5	27.4	2.2	0.03												
816	KY1604	Leg2	2D9	1-Nov-16	1:39	23.51	129.03	SCM	109	21.5	34.9	24.3		0.31												
817	KY1604	Leg2	2T2-1	1-Nov-16	17:31	23.31	129.56	0	0																	
818	KY1604	Leg2	2T2-1	1-Nov-16	17:31	23.31	129.56	10	10	28.9		21.9	6.4													
819	KY1604	Leg2	2T2-1	1-Nov-16	17:31	23.31	129.56	50	50	28.3		22.1	6.6													
820	KY1604	Leg2	2T2-1	1-Nov-16	17:31	23.31	129.56	75	75	27.1		22.6	6.9													
821	KY1604	Leg2	2T2-1	1-Nov-16	17:31	23.31	129.56	100	100	24.1		23.6	7.1													
822	KY1604	Leg2	2T2-1	1-Nov-16	17:31	23.31	129.56	150	150																	
823	KY1604	Leg2	2T2-1	1-Nov-16	17:31	23.31	129.56	200	200																	
824	KY1604	Leg2	2T2-1	1-Nov-16	17:31	23.31	129.56	300	300																	
825	KY1604	Leg2	2T2-1	1-Nov-16	17:31	23.31	129.56	500	500																	
826	KY1604	Leg2	2T2-1	1-Nov-16	17:31	23.31	129.56	750	750																	
827	KY1604	Leg2	2T2-1	1-Nov-16	17:31	23.31	129.56	1000	1000																	
828	KY1604	Leg2	2T2-1	1-Nov-16	17:31	23.31	129.56	SCM																		
829	KY1604	Leg2	E1	4-Nov-16	18:00	23.75	124.00	0	0	26.7	34.7															
830	KY1604	Leg2	E1	4-Nov-16	18:00	23.75	124.00	10	10	27.2	34.7	22.4	6.2	0.09												
831	KY1604	Leg2	E1	4-Nov-16	18:00	23.75	124.00	50	50	27.2	34.7	22.4	6.1	0.08												
832	KY1604	Leg2	E1	4-Nov-16	18:00	23.75	124.00	75	75	25.7	34.8	23.0	6.3	0.15												
833	KY1604	Leg2	E1	4-Nov-16	18:00	23.75	124.00	100	100	24.2	34.8	23.5	6.0	0.15												
834	KY1604	Leg2	E1	4-Nov-16	18:00	23.75	124.00	150	150	19.8	34.9	24.7	5.7	0.09												
835	KY1604	Leg2	E1	4-Nov-16	18:00	23.75	124.00	200	200	18.1	34.8	25.1	5.7	0.03												
836	KY1604	Leg2	E1	4-Nov-16	18:00	23.75	124.00	300	300	15.1	34.6	25.6	5.4	0.02												
837	KY1604	Leg2	E1	4-Nov-16	18:00	23.75	124.00	500	500	9.6	34.3	26.4	4.1	0.02												
838	KY1604	Leg2	E1	4-Nov-16	18:00	23.75	124.00	750	750	5.6	34.3	27.0	2.4	0.03												
839	KY1604	Leg2	E1	4-Nov-16	18:00	23.75	124.00	1000	1000	4.0	34.5	27.4	2.3	0.03												
840	KY1604	Leg2	E1	4-Nov-16	18:00	23.75	124.00	SCM	88	24.9	34.8	23.2		0.18												

Supplementary Table 1. Continued 7

No	Cruise	Leg	Station	Date	Time	Latitude [°N]	Longitude [°E]	Layer	Depth [m]	Temperature [°C]	Salinity [psu]	Sigma-t [kg/m^3]	DO [mL/L]	FISP [μg/L]	NO3 [μM]	NO2 [μM]	Si [μM]	PO4 [μM]	Chl-a [μg/L]	Chl-a >10 μm [μg/L]	Chl-a 3-10 μm [μg/L]	Chl-a 0.2-3 μm [μg/L]	Picoeukaryotes [cells/mL]	Synechococcus [cells/mL]	Dinoflagellate [cells/L]	Other phytoplankton [cells/L]
841	KY1604	Leg2	E4	4-Nov-16	22:42	23.27	124.02	0	0	27.5	34.3															
842	KY1604	Leg2	E4	4-Nov-16	22:42	23.27	124.02	10	10	27.5	34.3	22.0	6.1	0.12												
843	KY1604	Leg2	E4	4-Nov-16	22:42	23.27	124.02	50	50	27.4	34.4	22.1	6.1	0.08												
844	KY1604	Leg2	E4	4-Nov-16	22:42	23.27	124.02	75	75	27.2	34.7	22.4	6.1	0.08												
845	KY1604	Leg2	E4	4-Nov-16	22:42	23.27	124.02	100	100	26.1	34.7	22.8	6.2	0.13												
846	KY1604	Leg2	E4	4-Nov-16	22:42	23.27	124.02	150	150	23.1	34.9	23.8	5.9	0.13												
847	KY1604	Leg2	E4	4-Nov-16	22:42	23.27	124.02	200	200	18.7	34.8	25.0	5.7	0.03												
848	KY1604	Leg2	E4	4-Nov-16	22:42	23.27	124.02	300	300	15.4	34.6	25.6	5.4	0.02												
849	KY1604	Leg2	E4	4-Nov-16	22:42	23.27	124.02	500	500	9.2	34.2	26.5	4.2	0.02												
850	KY1604	Leg2	E4	4-Nov-16	22:42	23.27	124.02	750	750	5.2	34.3	27.1	2.3	0.03												
851	KY1604	Leg2	E4	4-Nov-16	22:42	23.27	124.02	1000	1000	3.7	34.4	27.4	2.0	0.03												
852	KY1604	Leg2	E4	4-Nov-16	22:42	23.27	124.02	SCM	138	23.7	34.9	23.6		0.17												
853	KY1604	Leg2	E6	5-Nov-16	22:34	23.25	125.00	0	0	27.8	34.6															
854	KY1604	Leg2	E6	5-Nov-16	22:34	23.25	125.00	10	10	28.0	34.6	22.1	6.1	0.09												
855	KY1604	Leg2	E6	5-Nov-16	22:34	23.25	125.00	50	50	28.0	34.6	22.1	6.0	0.09												
856	KY1604	Leg2	E6	5-Nov-16	22:34	23.25	125.00	75	75	28.0	34.6	22.1	6.0	0.09												
857	KY1604	Leg2	E6	5-Nov-16	22:34	23.25	125.00	100	100	27.3	34.7	22.4	5.9	0.16												
858	KY1604	Leg2	E6	5-Nov-16	22:34	23.25	125.00	150	150	24.2	35.0	23.6	5.8	0.07												
859	KY1604	Leg2	E6	5-Nov-16	22:34	23.25	125.00	200	200	20.9	34.9	24.5	5.7	0.03												
860	KY1604	Leg2	E6	5-Nov-16	22:34	23.25	125.00	300	300	16.8	34.7	25.3	5.7	0.02												
861	KY1604	Leg2	E6	5-Nov-16	22:34	23.25	125.00	500	500	10.4	34.3	26.3	4.8	0.02												
862	KY1604	Leg2	E6	5-Nov-16	22:34	23.25	125.00	750	750	5.8	34.3	27.0	2.4	0.03												
863	KY1604	Leg2	E6	5-Nov-16	22:34	23.25	125.00	1050	1050	4.2	34.5	27.3	2.5	0.03												
864	KY1604	Leg2	E6	5-Nov-16	22:34	23.25	125.00	SCM	95	27.6	34.7	22.3		0.19												
865	KY1604	Leg2	E7	5-Nov-16	3:12	22.75	123.97	0	0	28.2	34.6															
866	KY1604	Leg2	E7	5-Nov-16	3:12	22.75	123.97	10	10	28.2	34.6	22.0	6.0	0.07												
867	KY1604	Leg2	E7	5-Nov-16	3:12	22.75	123.97	50	50	28.2	34.6	22.0	6.0	0.08												
868	KY1604	Leg2	E7	5-Nov-16	3:12	22.75	123.97	75	75	28.2	34.6	22.0	6.0	0.08												
869	KY1604	Leg2	E7	5-Nov-16	3:12	22.75	123.97	100	100	28.2	34.6	22.0	6.0	0.07												
870	KY1604	Leg2	E7	5-Nov-16	3:12	22.75	123.97	150	150	24.6	34.9	23.4	5.8	0.09												
871	KY1604	Leg2	E7	5-Nov-16	3:12	22.75	123.97	200	200	21.2	35.0	24.4	5.7	0.04												
872	KY1604	Leg2	E7	5-Nov-16	3:12	22.75	123.97	300	300	16.4	34.7	25.4	5.4	0.02												
873	KY1604	Leg2	E7	5-Nov-16	3:12	22.75	123.97	500	500	10.0	34.3	26.4	4.2	0.02												
874	KY1604	Leg2	E7	5-Nov-16	3:12	22.75	123.97	750	750	5.2	34.3	27.1	2.3	0.03												
875	KY1604	Leg2	E7	5-Nov-16	3:12	22.75	123.97	1000	1000	3.8	34.4	27.4	2.0	0.03												
876	KY1604	Leg2	E7	5-Nov-16	3:12	22.75	123.97	SCM	129	25.5	34.9	23.1		0.17												
877	KY1604	Leg2	E9	5-Nov-16	18:00	22.75	125.00	0	0	27.9	34.6				0.12	0.00	1.22	-0.03	0.12							
878	KY1604	Leg2	E9	5-Nov-16	18:00	22.75	125.00	10	10	28.0	34.6	22.1	6.1	0.09	0.00	-0.01	1.26	-0.02	0.11							
879	KY1604	Leg2	E9	5-Nov-16	18:00	22.75	125.00	50	50	28.0	34.6	22.1	6.0	0.10	-0.01	-0.01	1.22	0.03	0.14							
880	KY1604	Leg2	E9	5-Nov-16	18:00	22.75	125.00	75	75	28.0	34.6	22.1	6.0	0.11												
881	KY1604	Leg2	E9	5-Nov-16	18:00	22.75	125.00	100	100	28.0	34.6	22.1	6.0	0.14	-0.02	0.00	1.27	0.02	0.21							
882	KY1604	Leg2	E9	5-Nov-16	18:00	22.75	125.00	150	150	24.1	35.0	23.6	5.8	0.07												
883	KY1604	Leg2	E9	5-Nov-16	18:00	22.75	125.00	200	200	21.1	35.0	24.4	5.6	0.05	2.42	0.03	2.58	0.16	0.04							
884	KY1604	Leg2	E9	5-Nov-16	18:00	22.75	125.00	300	300	16.7	34.7	25.3	5.7	0.02												
885	KY1604	Leg2	E9	5-Nov-16	18:00	22.75	125.00	500	500	10.9	34.4	26.3	4.4	0.02												
886	KY1604	Leg2	E9	5-Nov-16	18:00	22.75	125.00	750	750	6.4	34.3	26.9	2.7	0.03												
887	KY1604	Leg2	E9	5-Nov-16	18:00	22.75	125.00	1050	1050	4.0	34.5	27.4	2.3	0.03												
888	KY1604	Leg2	E9	5-Nov-16	18:00	22.75	125.00	SCM	100	28.0	34.6	22.1	6.0	0.14												
889	KY1604	Leg2	E3	6-Nov-16	2:23	23.71	125.01	0	0	27.4	34.6															
890	KY1604	Leg2	E3	6-Nov-16	2:23	23.71	125.01	10	10	27.6	34.6	22.3	6.1	0.13												
891	KY1604	Leg2	E3	6-Nov-16	2:23	23.71	125.01	50	50	27.6	34.6	22.3	6.0	0.14												
892	KY1604	Leg2	E3	6-Nov-16	2:23	23.71	125.01	75	75	27.6	34.6	22.3	6.0	0.13												
893	KY1604	Leg2	E3	6-Nov-16	2:23	23.71	125.01	100	100	27.6	34.7	22.3	6.0	0.12												
894	KY1604	Leg2	E3	6-Nov-16	2:23	23.71	125.01	150	150	23.8	34.9	23.6	5.9	0.10												
895	KY1604	Leg2	E3	6-Nov-16	2:23	23.71	125.01	200	200	20.0	34.9	24.7	5.7	0.04												
896	KY1604	Leg2	E3	6-Nov-16	2:23	23.71	125.01	300	300	16.5	34.7	25.4	5.9	0.02												
897	KY1604	Leg2	E3	6-Nov-16	2:23	23.71	125.01	500	500	10.3	34.3	26.3	4.7	0.02												
898	KY1604	Leg2	E3	6-Nov-16	2:23	23.71	125.01	750	750	6.0	34.3	27.0	2.5	0.03												
899	KY1604	Leg2	E3	6-Nov-16	2:23	23.71	125.01	1050	1050	4.3	34.5	27.3	2.4	0.03												
900	KY1604	Leg2	E3	6-Nov-16	2:23	23.71	125.01	SCM	97	27.6	34.6	22.3		0.15												
901	KY1604	Leg2	E5	6-Nov-16	22:27	23.23	124.50	0	0	28.0	34.6															
902	KY1604	Leg2	E5	6-Nov-16	22:27	23.23	124.50	10	10	28.0	34.6	22.1	6.0	0.08												
903	KY1604	Leg2	E5	6-Nov-16	22:27	23.23	124.50	50	50	28.0	34.6	22.1	6.0	0.09												
904	KY1604	Leg2	E5	6-Nov-16	22:27	23.23	124.50	75	75	28.0	34.6	22.1	6.0	0.08												
905	KY1604	Leg2	E5	6-Nov-16	22:27	23.23	124.50	100	100	28.1	34.6	22.1	6.0	0.09												
906	KY1604	Leg2	E5	6-Nov-16	22:27	23.23	124.50	150	150	24.6	34.9	23.4	5.8	0.08												
907	KY1604	Leg2	E5	6-Nov-16	22:27	23.23	124.50	200	200	21.6	34.9	24.3	5.6	0.04												
908	KY1604	Leg2	E5	6-Nov-16	22:27	23.23	124.50	300	300	16.6	34.7	25.4	5.7	0.02												
909	KY1604	Leg2	E5	6-Nov-16	22:27	23.23	124.50	500	500	10.4	34.3	26.3	4.3	0.02												
910	KY1604	Leg2	E5	6-Nov-16	22:27	23.23	124.50	750	750	5.6	34.2	27.0	2.4	0.03												
911	KY1604	Leg2	E5	6-Nov-16	22:27	23.23	124.50	1050	1050	3.9	34.5	27.4	2.5	0.03												
912	KY1604	Leg2	E5	6-Nov-16	22:27	23.23	124.50	SCM	119	27.4	34.7	22.4		0.20												
913	KY1604	Leg2	E8	6-Nov-16	18:00	22.75	124.50	0	0	28.2	34.6															
914	KY1604	Leg2	E8	6-Nov-16	18:00	22.75	124.50	10	10	28.1	34.6	22.0	6.1	0.07												
915	KY1604	Leg2	E8	6-Nov-16	18:00	22.75	124.50	50	50	28.1	34.6	22.0	6.0	0.08												
916	KY1604	Leg2	E8	6-Nov-16	18:00	22.75	124.50	75	75	28.1	34.6	22.0	6.0	0.10												
917	KY1604	Leg2	E8	6-Nov-16	18:00	22.75	124.50	100	100	28.1	34.6	22.0	6.0	0.14												
918	KY1604	Leg2	E8	6-Nov-16	18:00	22.75	124.50	150	150	26.0	35.0	23.0	5.9	0.11												
919	KY1604	Leg2	E8	6-Nov-16	18:00	22.75	124.50	200	200	22.3	35.0	24.1	5.8	0.06												
920	KY1604	Leg2	E8	6-Nov-16	18:00	22.75	124.50	300	300	17.6	34.7	25.2	5.6	0.02												
921	KY1604	Leg2	E8	6-Nov-16	18:00	22.75	124.50	500	500	10.8	34.3	26.3	4.5	0.02												
922	KY1604	Leg2	E8	6-Nov-16	18:00	22.75	124.50	750	750	5.8	34.3	27.0	2.5	0.03												
923	KY1604	Leg2	E8	6-Nov-16	18:00	22.75	124.50	1050	1050	4.0	34.4	27.4	2.2	0.03												
924	KY1604	Leg2	E8	6-Nov-16	18:00	22.75	124.50	SCM	132	26.9	34.9	22.7		0.15												
925	KY1604	Leg2	E2	7-Nov-16	2:38	23.72	124.51	0	0	27.6	34.6															
926	KY1604	Leg2	E2	7-Nov-16	2:38	23.72	124.51	10	10	27.5	34.6	22.2	6.1	0.09												
927	KY1604	Leg2	E2	7-Nov-16	2:38	23.72	124.51	50	50	27.6	34.6	22.2	6.0	0.08												
928	KY1604	Leg2	E2	7-Nov-16	2:38	23.72	124.51	75	75	27.6	34.7	22.3	6.0	0.10												
929	KY1604	Leg2	E2	7-Nov-16	2:38	23.72	124.51	100	100	26.9	34.7	22.5	5.9	0.15												
930	KY1604	Leg2	E2	7-Nov-16	2:38	23.72	124.51	150	150	23.7	34.8	23.6	5.9	0.12												
931	KY1604	Leg2	E2	7-Nov-16	2:38	23.72	124.51	200	200	19.8	34.9	24.7	5.7	0.05												
932	KY1604	Leg2	E2	7-Nov-16	2:38	23.72	124.51	300	300	16.2	34.6	25.4	5.5	0.02												
933	KY1604	Leg2	E2	7-Nov-16	2:38	23.72	124.51	500	500	10.0	34.3	26.4	4.2	0.02												
934	KY1604	Leg2	E2	7-Nov-16	2:38	23.72	124.51	750	750	5.4	34.3	27.1	2.3	0.03												
935	KY1604	Leg2	E2	7-Nov-16	2:38	23.72	124.51	1050	1050	3.8	34.5	27.4	2.4	0.03												
936	KY1604	Leg2	E2	7-Nov-16	2:38	23.72	124.51	SCM	100	26.9	34.7	22.5		0.15												

Supplementary Table 2. Sample information used for metagenomic analysis of larval eel gut contents and POM in this study

No	Sample name	Sample type	ID	Cruise	Leg	Station	Date	Time	Latitude [°N]	Longitude [°E]	Layer	Depth [m]	Pore size [μm]	Net depth [m]	Kingdom	Phylum	Class	Order	Family	Genus	Species	Scientific name	Abb name	Species ID	Taxinomic rank	Total length [mm]
1	N_1002_F_S75	Eel	1002	KY1604	Leg2	E5	6-Nov-16	22:27	23.23	124.50				125	Animalia	Chordata	Actinopterygii	Anguilliformes	Anguillidae	Anguilla	japonica	Anguilla japonica	AngJap	AngJap01	Species	54.7
2	N_152_F_S12	Eel	152	KY1604	Leg1	1AJ2	8-Oct-16	20:13	20.53	132.35				168	Animalia	Chordata	Actinopterygii	Anguilliformes	Anguillidae	Anguilla	japonica	Anguilla japonica	AngJap	AngJap02	Species	48.7
3	N_153_F_S13	Eel	153	KY1604	Leg1	1AJ2	8-Oct-16	20:13	20.53	132.35				168	Animalia	Chordata	Actinopterygii	Anguilliformes	Anguillidae	Anguilla	japonica	Anguilla japonica	AngJap	AngJap03	Species	50
4	N_159_F_S14	Eel	159	KY1604	Leg1	1AJ3	8-Oct-16	21:48	20.54	132.30				104	Animalia	Chordata	Actinopterygii	Anguilliformes	Anguillidae	Anguilla	japonica	Anguilla japonica	AngJap	AngJap04	Species	48.6
5	N_165_F_S15	Eel	165	KY1604	Leg1	1AJ4	8-Oct-16	23:00	20.56	132.26				80	Animalia	Chordata	Actinopterygii	Anguilliformes	Anguillidae	Anguilla	japonica	Anguilla japonica	AngJap	AngJap05	Species	50.7
6	N_166_F_S16	Eel	166	KY1604	Leg1	1AJ4	8-Oct-16	23:00	20.56	132.26				80	Animalia	Chordata	Actinopterygii	Anguilliformes	Anguillidae	Anguilla	japonica	Anguilla japonica	AngJap	AngJap06	Species	52.8
7	N_168_F_S17	Eel	168	KY1604	Leg1	1AJ4	8-Oct-16	23:00	20.56	132.26				80	Animalia	Chordata	Actinopterygii	Anguilliformes	Anguillidae	Anguilla	japonica	Anguilla japonica	AngJap	AngJap07	Species	46.9
8	N_171_F_S18	Eel	171	KY1604	Leg1	1AJ5	8-Oct-16	0:16	20.58	132.24				57	Animalia	Chordata	Actinopterygii	Anguilliformes	Anguillidae	Anguilla	japonica	Anguilla japonica	AngJap	AngJap08	Species	51.3
9	N_172_F_S19	Eel	172	KY1604	Leg1	1AJ5	8-Oct-16	0:16	20.58	132.24				57	Animalia	Chordata	Actinopterygii	Anguilliformes	Anguillidae	Anguilla	japonica	Anguilla japonica	AngJap	AngJap09	Species	54.2
10	N_173_F_S20	Eel	173	KY1604	Leg1	1AJ5	8-Oct-16	0:16	20.58	132.24				57	Animalia	Chordata	Actinopterygii	Anguilliformes	Anguillidae	Anguilla	japonica	Anguilla japonica	AngJap	AngJap10	Species	47.7
11	N_21_F_S2	Eel	21	KY1604	Leg1	1T6	30-Sep-16	0:13	14.01	133.02				208	Animalia	Chordata	Actinopterygii	Anguilliformes	Anguillidae	Anguilla	marmorata	Anguilla marmorata	AngMar	AngMar01	Species	15.2
12	N_22_F_S3	Eel	22	KY1604	Leg1	1T6	30-Sep-16	0:13	14.01	133.02				208	Animalia	Chordata	Actinopterygii	Anguilliformes	Anguillidae	Anguilla	marmorata	Anguilla marmorata	AngMar	AngMar02	Species	16.1
13	N_288_R_S21	Eel	288	KY1604	Leg2	2A6	20-Oct-16	22:31	22.50	131.00				221	Animalia	Chordata	Actinopterygii	Anguilliformes	Anguillidae	Anguilla	japonica	Anguilla japonica	AngJap	AngJap11	Species	52.9
14	N_289_R_S22	Eel	289	KY1604	Leg2	2A6	20-Oct-16	22:31	22.50	131.00				221	Animalia	Chordata	Actinopterygii	Anguilliformes	Anguillidae	Anguilla	japonica	Anguilla japonica	AngJap	AngJap12	Species	47
15	N_290_R_S23	Eel	290	KY1604	Leg2	2A6	20-Oct-16	22:31	22.50	131.00				221	Animalia	Chordata	Actinopterygii	Anguilliformes	Anguillidae	Anguilla	japonica	Anguilla japonica	AngJap	AngJap13	Species	49.2
16	N_292_R_S24	Eel	292	KY1604	Leg2	2A6	20-Oct-16	22:31	22.50	131.00				221	Animalia	Chordata	Actinopterygii	Anguilliformes	Anguillidae	Anguilla	japonica	Anguilla japonica	AngJap	AngJap14	Species	53.5
17	N_304_R_S25	Eel	304	KY1604	Leg2	2A7	20-Oct-16	3:00	22.03	131.02					Animalia	Chordata	Actinopterygii	Anguilliformes	Anguillidae	Anguilla	japonica	Anguilla japonica	AngJap	AngJap15	Species	48.1
18	N_305_R_S26	Eel	305	KY1604	Leg2	2A7	20-Oct-16	3:00	22.03	131.02					Animalia	Chordata	Actinopterygii	Anguilliformes	Anguillidae	Anguilla	japonica	Anguilla japonica	AngJap	AngJap16	Species	50
19	N_306_R_S27	Eel	306	KY1604	Leg2	2A7	20-Oct-16	3:00	22.03	131.02					Animalia	Chordata	Actinopterygii	Anguilliformes	Anguillidae	Anguilla	japonica	Anguilla japonica	AngJap	AngJap17	Species	48.6
20	N_31_F_S4	Eel	31	KY1604	Leg1	1T7	1-Oct-16	16:55	14.00	132.00				200	Animalia	Chordata	Actinopterygii	Anguilliformes	Anguillidae	Anguilla	marmorata	Anguilla marmorata	AngMar	AngMar03	Species	25
21	N_311_R_S28	Eel	311	KY1604	Leg2	2T1	21-Oct-16	22:24	22.01	130.58				100	Animalia	Chordata	Actinopterygii	Anguilliformes	Anguillidae	Anguilla	japonica	Anguilla japonica	AngJap	AngJap18	Species	52.9
22	N_312_R_S29	Eel	312	KY1604	Leg2	2T1	21-Oct-16	22:24	22.01	130.58				100	Animalia	Chordata	Actinopterygii	Anguilliformes	Anguillidae	Anguilla	japonica	Anguilla japonica	AngJap	AngJap19	Species	49.2
23	N_313_R_S30	Eel	313	KY1604	Leg2	2T1	21-Oct-16	22:24	22.01	130.58				100	Animalia	Chordata	Actinopterygii	Anguilliformes	Anguillidae	Anguilla	japonica	Anguilla japonica	AngJap	AngJap20	Species	49.7
24	N_33_F_S5	Eel	33	KY1604	Leg1	1T7	1-Oct-16	16:55	14.00	132.00				200	Animalia	Chordata	Actinopterygii	Anguilliformes	Anguillidae	Anguilla	marmorata	Anguilla marmorata	AngMar	AngMar04	Species	14.3
25	N_348_F_S31	Eel	348	KY1604	Leg2	2T5	21-Oct-16	2:57	22.03	130.50				90	Animalia	Chordata	Actinopterygii	Anguilliformes	Anguillidae	Anguilla	japonica	Anguilla japonica	AngJap	AngJap21	Species	53.2
26	N_349_F_S32	Eel	349	KY1604	Leg2	2T5	21-Oct-16	2:57	22.03	130.50				90	Animalia	Chordata	Actinopterygii	Anguilliformes	Anguillidae	Anguilla	japonica	Anguilla japonica	AngJap	AngJap22	Species	52.3
27	N_350_F_S33	Eel	350	KY1604	Leg2	2T5	21-Oct-16	2:57	22.03	130.50				90	Animalia	Chordata	Actinopterygii	Anguilliformes	Anguillidae	Anguilla	japonica	Anguilla japonica	AngJap	AngJap23	Species	46.3
28	N_351_F_S34	Eel	351	KY1604	Leg2	2T5	21-Oct-16	2:57	22.03	130.50				90	Animalia	Chordata	Actinopterygii	Anguilliformes	Anguillidae	Anguilla	japonica	Anguilla japonica	AngJap	AngJap24	Species	48.5
29	N_40_F_S6	Eel	40	KY1604	Leg1	1T7	1-Oct-16	16:55	14.00	132.00				200	Animalia	Chordata	Actinopterygii	Anguilliformes	Anguillidae	Anguilla	marmorata	Anguilla marmorata	AngMar	AngMar05	Species	23.1
30	N_404_RI_S85	Eel	404	KY1604	Leg2	2A19	24-Oct-16	2:00	16.03	131.00				200	Animalia	Chordata	Actinopterygii	Anguilliformes	Anguillidae	Anguilla	japonica	Anguilla japonica	AngJap	AngJap25	Species	42.6
31	N_409_F_S36	Eel	409	KY1604	Leg2	2A21	25-Oct-16	16:54	15.00	131.00				220	Animalia	Chordata	Actinopterygii	Anguilliformes	Serrivomeridae			Serrivomeridae	Serriv	Serriv01	Family	22.2
32	N_41_F_S7	Eel	41	KY1604	Leg1	1T7	1-Oct-16	16:55	14.00	132.00				200	Animalia	Chordata	Actinopterygii	Anguilliformes	Anguillidae	Anguilla	marmorata	Anguilla marmorata	AngMar	AngMar06	Species	13
33	N_410_F_S37	Eel	410	KY1604	Leg2	2A21	25-Oct-16	16:54	15.00	131.00				220	Animalia	Chordata	Actinopterygii	Anguilliformes	Serrivomeridae			Serrivomeridae	Serriv	Serriv02	Family	30.3
34	N_411_F_S38	Eel	411	KY1604	Leg2	2A21	25-Oct-16	16:54	15.00	131.00				220	Animalia	Chordata	Actinopterygii	Anguilliformes	Serrivomeridae			Serrivomeridae	Serriv	Serriv03	Family	27.1
35	N_416_F_S39	Eel	416	KY1604	Leg2	2A22	25-Oct-16	22:07	14.50	131.00				200	Animalia	Chordata	Actinopterygii	Anguilliformes	Serrivomeridae			Serrivomeridae	Serriv	Serriv04	Family	37.7
36	N_417_F_S40	Eel	417	KY1604	Leg2	2A22	25-Oct-16	22:07	14.50	131.00				200	Animalia	Chordata	Actinopterygii	Anguilliformes	Serrivomeridae			Serrivomeridae	Serriv	Serriv05	Family	38.3
37	N_418_F_S41	Eel	418	KY1604	Leg2	2A22	25-Oct-16	22:07	14.50	131.00				200	Animalia	Chordata	Actinopterygii	Anguilliformes	Serrivomeridae			Serrivomeridae	Serriv	Serriv06	Family	41
38	N_419_F_S42	Eel	419	KY1604	Leg2	2A22	25-Oct-16	22:07	14.50	131.00				200	Animalia	Chordata	Actinopterygii	Anguilliformes	Serrivomeridae			Serrivomeridae	Serriv	Serriv07	Family	28.9
39	N_42_F_S8	Eel	42	KY1604	Leg1	1T7	1-Oct-16	16:55	14.00	132.00				200	Animalia	Chordata	Actinopterygii	Anguilliformes	Anguillidae	Anguilla	marmorata	Anguilla marmorata	AngMar	AngMar07	Species	24.6
40	N_420_F_S43	Eel	420	KY1604	Leg2	2A22	25-Oct-16	22:07	14.50	131.00				200	Animalia	Chordata	Actinopterygii	Anguilliformes	Serrivomeridae			Serrivomeridae	Serriv	Serriv08	Family	25
41	N_426_F_S44	Eel	426	KY1604	Leg2	2A23	25-Oct-16	2:16	14.00	131.00				200	Animalia	Chordata	Actinopterygii	Anguilliformes	Anguillidae	Anguilla	marmorata	Anguilla marmorata	AngMar	AngMar08	Species	25.1
42	N_43_F_S9	Eel	43	KY1604	Leg1	1T7	1-Oct-16	16:55	14.00	132.00				200	Animalia	Chordata	Actinopterygii	Anguilliformes	Anguillidae	Anguilla	marmorata	Anguilla marmorata	AngMar	AngMar09	Species	27.5
43	N_438_F_S45	Eel	438	KY1604	Leg2	2B2	26-Oct-16	22:42	14.50	129.00				200	Animalia	Chordata	Actinopterygii	Anguilliformes	Anguillidae	Anguilla	marmorata	Anguilla marmorata	AngMar	AngMar10	Species	27.9
44	N_450_RI6_S91	Eel	91	KY1604	Leg1	1G7	6-Oct-16	1:30	20.50	132.52				205	Animalia	Chordata	Actinopterygii	Anguilliformes	Anguillidae	Anguilla	japonica	Anguilla japonica	AngJap	AngJap26	Species	41.9
45	N_450_RI7_S92	Eel	92	KY1604	Leg1	1G7	6-Oct-16	1:30	20.50	132.52				205	Animalia	Chordata	Actinopterygii	Anguilliformes	Anguillidae	Anguilla	japonica	Anguilla japonica	AngJap	AngJap27	Species	42.3
46	N_46_F_S10	Eel	46	KY1604	Leg1	1T8	1-Oct-16	1:29	13.98	131.02				216	Animalia	Chordata	Actinopterygii	Anguilliformes	Anguillidae	Anguilla	marmorata	Anguilla marmorata	AngMar	AngMar11	Species	38.3
47	N_47_F_S11	Eel	47	KY1604	Leg1	1T8	1-Oct-16	1:29	13.98	131.02				216	Animalia	Chordata	Actinopterygii	Anguilliformes	Anguillidae	Anguilla	marmorata	Anguilla marmorata	AngMar	AngMar12	Species	31.5
48	N_472_RI3_S99	Eel	99	KY1604	Leg1	1G7	6-Oct-16	1:30	20.50	132.52				205	Animalia	Chordata	Actinopterygii	Anguilliformes	Serrivomeridae			Serrivomeridae	Serriv	Serriv09	Family	35.8
49	N_472_RI4_S100	Eel	100	KY1604	Leg1	1G7	6-Oct-16	1:30	20.50	132.52				205	Animalia	Chordata	Actinopterygii	Anguilliformes	Serrivomeridae			Serrivomeridae	Serriv	Serriv10	Family	40.1
50	N_505_F_S46	Eel	505	KY1604	Leg2	2D1	29-Oct-16	17:55	22.50	128.00				100	Animalia	Chordata	Actinopterygii	Anguilliformes	Anguillidae	Anguilla	japonica	Anguilla japonica	AngJap	AngJap28	Species	58.2
51	N_585_F10_S56	Eel	594	KY1604	Leg2	2D6	30-Oct-16	16:56	23.00	130.00				100	Animalia	Chordata	Actinopterygii	Anguilliformes	Congridae	Gnathophis		Gnathophis	Gnatho	Gnatho01	Genus	36.2
52	N_585_F11_S57	Eel	595	KY1604	Leg2	2D6	30-Oct-16	16:56	23.00	130.00				100	Animalia	Chordata	Actinopterygii	Anguilliformes	Congridae	Gnathophis		Gnathophis	Gnatho	Gnatho02	Genus	34.7
53	N_585_F12_S58	Eel	596	KY1604	Leg2	2D6	30-Oct-16	16:56	23.00	130.00				100	Animalia	Chordata	Actinopterygii	Anguilliformes	Congridae	Gnathophis		Gnathophis	Gnatho	Gnatho03	Genus	42.5
54	N_585_F13_S59	Eel	597	KY1604	Leg2	2D6	30-Oct-16	16:56	23.00	130.00				100	Animalia	Chordata	Actinopterygii	Anguilliformes	Congridae	Gnathophis		Gnathophis	Gnatho	Gnatho04	Genus	40.5
55	N_585_F14_S60	Eel	598	KY1604	Leg2	2D6	30-Oct-16	16:56	23.00	130.00				100	Animalia	Chordata	Actinopterygii	Anguilliformes	Congridae	Gnathophis		Gnathophis	Gnatho	Gnatho05	Genus	32.1
56	N_585_F15_S61	Eel	599	KY1604	Leg2	2D6	30-Oct-16	16:56	23.00	130.00				100	Animalia	Chordata	Actinopterygii	Anguilliformes	Congridae	Gnathophis		Gnathophis	Gnatho	Gnatho06	Genus	33.3
57	N_585_F5_S51	Eel	589	KY1604	Leg2	2D6	30-Oct-16	16:56	23.00	130.00				100	Animalia	Chordata	Actinopterygii	Anguilliformes	Congridae	Gnathophis		Gnathophis	Gnatho	Gnatho12	Genus	58.9
58	N_585_F6_S52	Eel	590	KY1604	Leg2	2D6	30-Oct-16	16:56	23.00	130.00				100	Animalia	Chordata	Actinopterygii	Anguilliformes	Congridae	Gnathophis		Gnathophis	Gnatho	Gnatho13	Genus	56.4
59	N_585_F7_S53	Eel	591	KY1604	Leg2	2D6	30-Oct-16	16:56	23.00	130.00				100	Animalia	Chordata	Actinopterygii	Anguilliformes	Congridae	Gnathophis		Gnathophis	Gnatho	Gnatho14	Genus	35.5
60	N_585_F8_S54	Eel	592	KY1604	Leg2	2D6	30-Oct-16	16:56	23.00	130.00				100	Animalia	Chordata	Actinopterygii	Anguilliformes	Congridae	Gnathophis		Gnathophis	Gnatho	Gnatho15	Genus	41.9
61	N_585_F9_S55	Eel	593	KY1604	Leg2	2D6	30-Oct-16	16:56	23.00	130.00				100	Animalia	Chordata	Actinopterygii	Anguilliformes	Congridae	Gnathophis		Gnathophis	Gnatho	Gnatho16	Genus	38.2
62	N_632_F1_S64	Eel	632	KY1604	Leg2	2D5	30-Oct-16	21:56	22.52	130.01				105	Animalia	Chordata	Actinopterygii	Anguilliformes	Congridae	Gnathophis		Gnathophis	Gnatho	Gnatho17	Genus	45.7
63	N_632_F2_S65	Eel	633	KY1604	Leg2	2D5	30-Oct-16	21:56	22.52	130.01				105	Animalia	Chordata	Actinopterygii	Anguilliformes	Congridae	Gnathophis		Gnathophis	Gnatho	Gnatho18	Genus	52.5
64	N_632_F3_S66	Eel	634	KY1604	Leg2	2D5	30-Oct-16	21:56	22.52	130.01				105	Animalia	Chordata	Actinopterygii	Anguilliformes	Congridae	Gnathophis		Gnathophis	Gnatho	Gnatho19	Genus	40.3
65	N_632_F4_S67	Eel	635	KY1604	Leg2	2D5	30-Oct-16	21:56	22.52	130.01				105	Animalia	Chordata	Actinopterygii	Anguilliformes	Congridae	Gnathophis		Gnathophis	Gnatho	Gnatho20	Genus	42
66	N_632_F5_S68	Eel	636	KY1604	Leg2	2D5	30-Oct-16	21:56	22.52	130.01				105	Animalia	Chordata	Actinopterygii	Anguilliformes	Congridae	Gnathophis		Gnathophis	Gnatho	Gnatho21	Genus	58.3
67	N_649_F_S69	Eel	649	KY1604	Leg2	2D4	30-Oct-16	2:06	22.51	129.53				100	Animalia	Chordata	Actinopterygii	Anguilliformes	Anguillidae	Anguilla	japonica	Anguilla japonica	AngJap	AngJap29	Species	59.6
68	N_662_RI_S102	Eel	662	KY1604	Leg2	2D7	31-Oct-16	17:00	23.50	130.00				100	Animalia	Chordata	Actinopterygii	Anguilliformes	Anguillidae	Anguilla	japonica	Anguilla japonica	AngJap	AngJap30	Species	51.8
69	N_663_RI_S103	Eel	663	KY1604	Leg2	2D7	31-Oct-16	17:00	23.50	130.00				100	Animalia	Chordata	Actinopterygii	Anguilliformes	Anguillidae	Anguilla	japonica	Anguilla japonica	AngJap	AngJap31	Species	53.3
70	N_664_RI_S104	Eel	664	KY1604	Leg2	2D7	31-Oct-16	17:00	23.50	130.00				100	Animalia	Chordata	Actinopterygii	Anguilliformes	Anguillidae	Anguilla	japonica	Anguilla japonica	AngJap	AngJap32	Species	52.2
71	N_7_F_S1	Eel	7	KY1604	Leg1	1T3	29-Sep-16	15:59	14.00	136.00				220	Animalia	Chordata	Actinopterygii	Anguilliformes	Anguillidae	Anguilla	marmorata	Anguilla marmorata	AngMar	AngMar13	Species	39.8
72	N_832_F_S70	Eel	832	KY1604	Leg2	2D8	31-Oct-16	21:32	23.52	129.56				100	Animalia	Chordata	Actinopterygii	Anguilliformes	Anguillidae	Anguilla	japonica	Anguilla japonica	AngJap	AngJap33	Species	56.8
73	N_861_F_S71	Eel	861	KY1604	Leg2	2T2-1	1-Nov-16	17:31	23.31	129.56				100	Animalia	Chordata	Actinopterygii	Anguilliformes	Anguillidae	Anguilla	japonica	Anguilla japonica	AngJap	AngJap34	Species	49.9
74	N_862_F_S72	Eel	862	KY1604	Leg2	2T2-1	1-Nov-16	17:31	23.31	129.56				100	Animalia	Chordata	Actinopterygii	Anguilliformes	Anguillidae	Anguilla	japonica	Anguilla japonica	AngJap	AngJap35	Species	47.3
75	N_990_S_S74	Eel	990	KY1604	Leg2	E8	6-Nov-16	18:00	22.75	124.50				125	Animalia	Chordata	Actinopterygii	Anguilliformes	Anguillidae	Anguilla	japonica	Anguilla japonica	AngJap	AngJap36	Species	52.8
76	1G1_3_10m	POM	134	KY1604	Leg1	1G1	5-Oct-16	16:59	22.50	133.25	10	10	3											G1_3um_10m		
77	1G1_3_50m	POM	135	KY1604	Leg1	1G1	5-Oct-16	16:59	22.50	133.25	50	50	3											G1_3um_50m		
78	1G1_3_100m	POM	137	KY1604	Leg1	1G1	5-Oct-16	16:59	22.50	133.25	100	100	3											G1_3um_100m		
79	1G1_3_200m	POM	139	KY1604	Leg1	1G1	5-Oct-16	16:59	22.50	133.25	200	200	3											G1_3um_200m		
80	1G1_3_SCMm	POM	144	KY1604	Leg1	1G1	5-Oct-16	16:59	22.50	133.25	SCM	130	3											G1_3um_SCM		
81	1G6_3_10m	POM	206	KY1604	Leg1	1G6	7-Oct-16	16:59	20.87	132.67	10	10	3											G6_3um_10m		
82	1G6_3_50m	POM	207	KY1604	Leg1	1G6	7-Oct-16	16:59	20.87	132.67	50	50	3											G6_3um_50m		
83	1G6_3_100m	POM	209	KY1604	Leg1	1G6	7-Oct-16	16:59	20.87	132.67	100	100	3											G6_3um_100m		
84	1G6_3_200m	POM	211	KY1604	Leg1	1G6	7-Oct-16	16:59	20.87	132.67	200	200	3											G6_3um_200m		
85	1G6_3_SCMm	POM	216	KY1604	Leg1	1G6	7-Oct-16	16:59	20.87	132.67	SCM	126	3											G6_3um_SCM		
86	1G9_3_10m	POM	182	KY1604	Leg1	1G9	6-Oct-16	17:00	19.87	132.31	10	10	3											G9_3um_10m		
87	1G9_3_50m	POM	183	KY1604	Leg1	1G9	6-Oct-16	17:00	19.87	132.31	50	50	3											G9_3um_50m		
88	1G9_3_100m	POM	185	KY1604	Leg1	1G9	6-Oct-16	17:00	19.87	132.31	100	100	3											G9_3um_100m		
89	1G9_3_200m	POM	187	KY1604	Leg1	1G9	6-Oct-16	17:00	19.87	132.31	200	200	3											G9_3um_200m		
90	1T1_3_10m	POM	14	KY1604	Leg1	1T1	28-Sep-16	15:58	14.00	138.00	10	10	3											T1_3um_10m		
91	1T1_3_50m	POM	15	KY1604	Leg1	1T1	28-Sep-16	15:58	14.00	138.00	50	50	3											T1_3um_50m		
92	1T1_3_100m	POM	17	KY1604	Leg1	1T1	28-Sep-16	15:58	14.00	138.00	100	100	3											T1_3um_100m		
93	1T1_3_200m	POM	19	KY1604	Leg1	1T1	28-Sep-16	15:58	14.00	138.00	200	200	3											T1_3um_200m		
94	1T1_3_SCMm	POM	24	KY1604	Leg1	1T1	28-Sep-16	15:58	14.00	138.00	SCM	113	3											T1_3um_SCM		
95	1T10_3_10m	POM	98	KY1604	Leg1	1T10	2-Oct-16	16:56	14.00	129.00	10	10	3											T10_3um_10m		
96	1T10_3_50m	POM	99	KY1604	Leg1	1T10	2-Oct-16	16:56	14.00	129.00	50	50	3											T10_3um_50m		
97	1T10_3_100m	POM	101	KY1604	Leg1	1T10	2-Oct-16	16:56	14.00	129.00	100	100	3											T10_3um_100m		
98	1T10_3_200m	POM	103	KY1604	Leg1	1T10	2-Oct-16	16:56	14.00	129.00	200	200	3											T10_3um_200m		
99	1T10_3_SCMm	POM	108	KY1604	Leg1	1T10	2-Oct-16	16:56	14.00	129.00	SCM	112	3											T10_3um_SCM		
100	1T5_3_10m	POM	62	KY1604	Leg1	1T5	30-Sep-16	15:55	14.00	134.00	10	10	3											T5_3um_10m		

Supplementary Table 2. Continued 1

No	Sample name	Sample type	ID	Cruise	Leg	Station	Date	Time	Latitude [°N]	Longitude [°E]	Layer	Depth [m]	Pore size [μm]	Net depth [m]	Kingdom	Phylum	Class	Order	Family	Genus	Species	Scientific name	Abb name	Species ID	Taxinomic rank	Total length [mm]
101	1T5_3_50m	POM	63	KY1604	Leg1	1T5	30-Sep-16	15:55	14.00	134.00	50	50	3											T5_3um_50m		
102	1T5_3_100m	POM	65	KY1604	Leg1	1T5	30-Sep-16	15:55	14.00	134.00	100	100	3											T5_3um_100m		
103	1T5_3_200m	POM	67	KY1604	Leg1	1T5	30-Sep-16	15:55	14.00	134.00	200	200	3											T5_3um_200m		
104	2C1_3_10m	POM	662	KY1604	Leg2	2C1	27-Oct-16	17:27	16.00	128.00	10	10	3											C1_3um_10m		
105	2C1_3_50m	POM	663	KY1604	Leg2	2C1	27-Oct-16	17:27	16.00	128.00	50	50	3											C1_3um_50m		
106	2C1_3_100m	POM	665	KY1604	Leg2	2C1	27-Oct-16	17:27	16.00	128.00	100	100	3											C1_3um_100m		
107	2C1_3_200m	POM	667	KY1604	Leg2	2C1	27-Oct-16	17:27	16.00	128.00	200	200	3											C1_3um_200m		
108	2C1_3_SCMm	POM	672	KY1604	Leg2	2C1	27-Oct-16	17:27	16.00	128.00	SCM	112	3											C1_3um_SCM		
109	2C5_3_10m	POM	698	KY1604	Leg2	2C5	28-Oct-16	17:23	18.00	128.00	10	10	3											C5_3um_10m		
110	2C5_3_50m	POM	699	KY1604	Leg2	2C5	28-Oct-16	17:23	18.00	128.00	50	50	3											C5_3um_50m		
111	2C5_3_100m	POM	701	KY1604	Leg2	2C5	28-Oct-16	17:23	18.00	128.00	100	100	3											C5_3um_100m		
112	2C5_3_200m	POM	703	KY1604	Leg2	2C5	28-Oct-16	17:23	18.00	128.00	200	200	3											C5_3um_200m		
113	2C5_3_SCMm	POM	708	KY1604	Leg2	2C5	28-Oct-16	17:23	18.00	128.00	SCM	113	3											C5_3um_SCM		
114	2D7_3_10m	POM	794	KY1604	Leg2	2D7	31-Oct-16	17:00	23.50	130.00	10	10	3											D7_3um_10m		
115	2D7_3_50m	POM	795	KY1604	Leg2	2D7	31-Oct-16	17:00	23.50	130.00	50	50	3											D7_3um_50m		
116	2D7_3_100m	POM	797	KY1604	Leg2	2D7	31-Oct-16	17:00	23.50	130.00	100	100	3											D7_3um_100m		
117	2D7_3_200m	POM	799	KY1604	Leg2	2D7	31-Oct-16	17:00	23.50	130.00	200	200	3											D7_3um_200m		
118	2D7_3_SCMm	POM	804	KY1604	Leg2	2D7	31-Oct-16	17:00	23.50	130.00	SCM	100	3											D7_3um_SCM		
119	1G1_10_100m_S61	POM	137	KY1604	Leg1	1G1	5-Oct-16	16:59	22.50	133.25	100	100	10											G1_10um_100m		
120	1G1_10_10m_S59	POM	134	KY1604	Leg1	1G1	5-Oct-16	16:59	22.50	133.25	10	10	10											G1_10um_10m		
121	1G1_10_200m_S62	POM	139	KY1604	Leg1	1G1	5-Oct-16	16:59	22.50	133.25	200	200	10											G1_10um_200m		
122	1G1_10_50m_S60	POM	135	KY1604	Leg1	1G1	5-Oct-16	16:59	22.50	133.25	50	50	10											G1_10um_50m		
123	1G1_10_SCMm_S63	POM	144	KY1604	Leg1	1G1	5-Oct-16	16:59	22.50	133.25	SCM	130	10											G1_10um_SCM		
124	1G6_10_100m_S66	POM	209	KY1604	Leg1	1G6	7-Oct-16	16:59	20.87	132.67	100	100	10											G6_10um_100m		
125	1G6_10_10m_S64	POM	206	KY1604	Leg1	1G6	7-Oct-16	16:59	20.87	132.67	10	10	10											G6_10um_10m		
126	1G6_10_200m_S67	POM	211	KY1604	Leg1	1G6	7-Oct-16	16:59	20.87	132.67	200	200	10											G6_10um_200m		
127	1G6_10_50m_S65	POM	207	KY1604	Leg1	1G6	7-Oct-16	16:59	20.87	132.67	50	50	10											G6_10um_50m		
128	1G6_10_SCMm_S68	POM	216	KY1604	Leg1	1G6	7-Oct-16	16:59	20.87	132.67	SCM	126	10											G6_10um_SCM		
129	1G9_10_100m_S71	POM	185	KY1604	Leg1	1G9	6-Oct-16	17:00	19.87	132.31	100	100	10											G9_10um_100m		
130	1G9_10_10m_S69	POM	182	KY1604	Leg1	1G9	6-Oct-16	17:00	19.87	132.31	10	10	10											G9_10um_10m		
131	1G9_10_200m_S72	POM	187	KY1604	Leg1	1G9	6-Oct-16	17:00	19.87	132.31	200	200	10											G9_10um_200m		
132	1G9_10_50m_S70	POM	183	KY1604	Leg1	1G9	6-Oct-16	17:00	19.87	132.31	50	50	10											G9_10um_50m		
133	1T1_10_100m_S47	POM	17	KY1604	Leg1	1T1	28-Sep-16	15:58	14.00	138.00	100	100	10											T1_10um_100m		
134	1T1_10_10m_S45	POM	14	KY1604	Leg1	1T1	28-Sep-16	15:58	14.00	138.00	10	10	10											T1_10um_10m		
135	1T1_10_200m_S48	POM	19	KY1604	Leg1	1T1	28-Sep-16	15:58	14.00	138.00	200	200	10											T1_10um_200m		
136	1T1_10_50m_S46	POM	15	KY1604	Leg1	1T1	28-Sep-16	15:58	14.00	138.00	50	50	10											T1_10um_50m		
137	1T1_10_SCMm_S49	POM	24	KY1604	Leg1	1T1	28-Sep-16	15:58	14.00	138.00	SCM	113	10											T1_10um_SCM		
138	1T10_10_100m_S56	POM	101	KY1604	Leg1	1T10	2-Oct-16	16:56	14.00	129.00	100	100	10											T10_10um_100m		
139	1T10_10_10m_S54	POM	98	KY1604	Leg1	1T10	2-Oct-16	16:56	14.00	129.00	10	10	10											T10_10um_10m		
140	1T10_10_200m_S57	POM	103	KY1604	Leg1	1T10	2-Oct-16	16:56	14.00	129.00	200	200	10											T10_10um_200m		
141	1T10_10_50m_S55	POM	99	KY1604	Leg1	1T10	2-Oct-16	16:56	14.00	129.00	50	50	10											T10_10um_50m		
142	1T10_10_SCMm_S58	POM	108	KY1604	Leg1	1T10	2-Oct-16	16:56	14.00	129.00	SCM	112	10											T10_10um_SCM		
143	1T5_10_100m_S52	POM	65	KY1604	Leg1	1T5	30-Sep-16	15:55	14.00	134.00	100	100	10											T5_10um_100m		
144	1T5_10_10m_S50	POM	62	KY1604	Leg1	1T5	30-Sep-16	15:55	14.00	134.00	10	10	10											T5_10um_10m		
145	1T5_10_200m_S53	POM	67	KY1604	Leg1	1T5	30-Sep-16	15:55	14.00	134.00	200	200	10											T5_10um_200m		
146	1T5_10_50m_S51	POM	63	KY1604	Leg1	1T5	30-Sep-16	15:55	14.00	134.00	50	50	10											T5_10um_50m		
147	2C1_10_100m_S75	POM	665	KY1604	Leg2	2C1	27-Oct-16	17:27	16.00	128.00	100	100	10											C1_10um_100m		
148	2C1_10_10m_S73	POM	662	KY1604	Leg2	2C1	27-Oct-16	17:27	16.00	128.00	10	10	10											C1_10um_10m		
149	2C1_10_200m_S76	POM	667	KY1604	Leg2	2C1	27-Oct-16	17:27	16.00	128.00	200	200	10											C1_10um_200m		
150	2C1_10_50m_S74	POM	663	KY1604	Leg2	2C1	27-Oct-16	17:27	16.00	128.00	50	50	10											C1_10um_50m		
151	2C1_10_SCMm_S77	POM	672	KY1604	Leg2	2C1	27-Oct-16	17:27	16.00	128.00	SCM	112	10											C1_10um_SCM		
152	2C5_10_100m_S80	POM	701	KY1604	Leg2	2C5	28-Oct-16	17:23	18.00	128.00	100	100	10											C5_10um_100m		
153	2C5_10_10m_S78	POM	698	KY1604	Leg2	2C5	28-Oct-16	17:23	18.00	128.00	10	10	10											C5_10um_10m		
154	2C5_10_200m_S81	POM	703	KY1604	Leg2	2C5	28-Oct-16	17:23	18.00	128.00	200	200	10											C5_10um_200m		
155	2C5_10_50m_S79	POM	699	KY1604	Leg2	2C5	28-Oct-16	17:23	18.00	128.00	50	50	10											C5_10um_50m		
156	2C5_10_SCMm_S82	POM	708	KY1604	Leg2	2C5	28-Oct-16	17:23	18.00	128.00	SCM	113	10											C5_10um_SCM		
157	2D7_10_100m_S85	POM	797	KY1604	Leg2	2D7	31-Oct-16	17:00	23.50	130.00	100	100	10											D7_10um_100m		
158	2D7_10_10m_S83	POM	794	KY1604	Leg2	2D7	31-Oct-16	17:00	23.50	130.00	10	10	10											D7_10um_10m		
159	2D7_10_200m_S86	POM	799	KY1604	Leg2	2D7	31-Oct-16	17:00	23.50	130.00	200	200	10											D7_10um_200m		
160	2D7_10_50m_S84	POM	795	KY1604	Leg2	2D7	31-Oct-16	17:00	23.50	130.00	50	50	10											D7_10um_50m		
161	2D7_10_SCMm_S87	POM	804	KY1604	Leg2	2D7	31-Oct-16	17:00	23.50	130.00	SCM	100	10											D7_10um_SCM		
